# Electrochemical deoxygenative trifluoromethylallenylation of propargylic alcohols *via* sodium-mediated pre-association

**DOI:** 10.1039/d6sc02203k

**Published:** 2026-04-28

**Authors:** Jihoon Jang, Hyunwoo Kim, Eun Jin Cho

**Affiliations:** a Department of Chemistry, Chung-Ang University 84 Heukseok-ro, Dongjak-gu Seoul 06974 Republic of Korea ejcho@cau.ac.kr; b Department of Chemistry, Pohang University of Science and Technology (POSETECH) Pohang 37673 Republic of Korea

## Abstract

We report an electrochemical protocol that enables the direct conversion of free propargylic alcohols into trifluoromethylated allenes through sodium-mediated radical C–O bond activation. The transformation proceeds *via* a pre-association mechanism between the propargylic alcohol and a trifluoromethyl sulfinate reagent, which organizes the reactive complex through Na^+^ coordination. This interaction lowers the energetic barrier of the intrinsically endothermic C–O bond cleavage, allowing a concerted radical addition pathway under mild electrochemical conditions. Combined experimental and computational studies, including NMR titration, kinetic analysis, and DFT calculations, reveal that sodium acts as an ion bridge, enabling selective CF_3_ incorporation. The reaction exhibits broad substrate scope and high chemoselectivity, tolerating halogens, heteroarenes, and sensitive functional groups, and proving effective for the late-stage trifluoromethylation of natural products and pharmaceuticals.

## Abstract 2

Electrochemical conversion of free propargylic alcohols to trifluoromethylated allenes is enabled by sodium-mediated radical C–O bond activation through Na^+^-organized pre-association with a trifluoromethyl sulfinate reagent.

## Graphical abstract

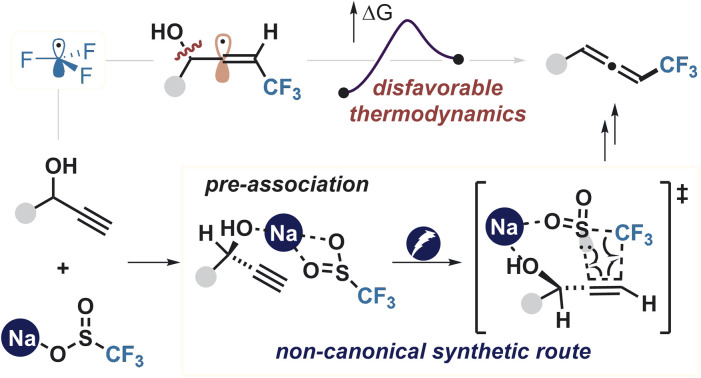

## Introduction

Allenes, cumulated dienes with a central sp-hybridized carbon and two orthogonal π-bonds,^1,2^ exhibit unique electronic structures that enable unusual reactivity.^3^ These features impart a polarized π-system and exceptional control of regio- and stereochemistry in various transformations.^4–8^ Consequently, allenes are not only common motifs in natural products but also versatile intermediates in the synthesis of complex pharmaceuticals and functional materials.^9–11^ Incorporating a trifluoromethyl (CF_3_) group into an allene framework further enhances these attributes. The CF_3_ group is a strongly electron-withdrawing, privileged motif in medicinal chemistry, known to improve pharmacophysical properties including metabolic stability and binding selectivity of drug candidates.^12–15^ The synergy between the allene core and a CF_3_ substituent creates a polarized, highly reactive π-system, offering a broadly useful platform for selective bond-forming reactions.

**Fig. 1:**
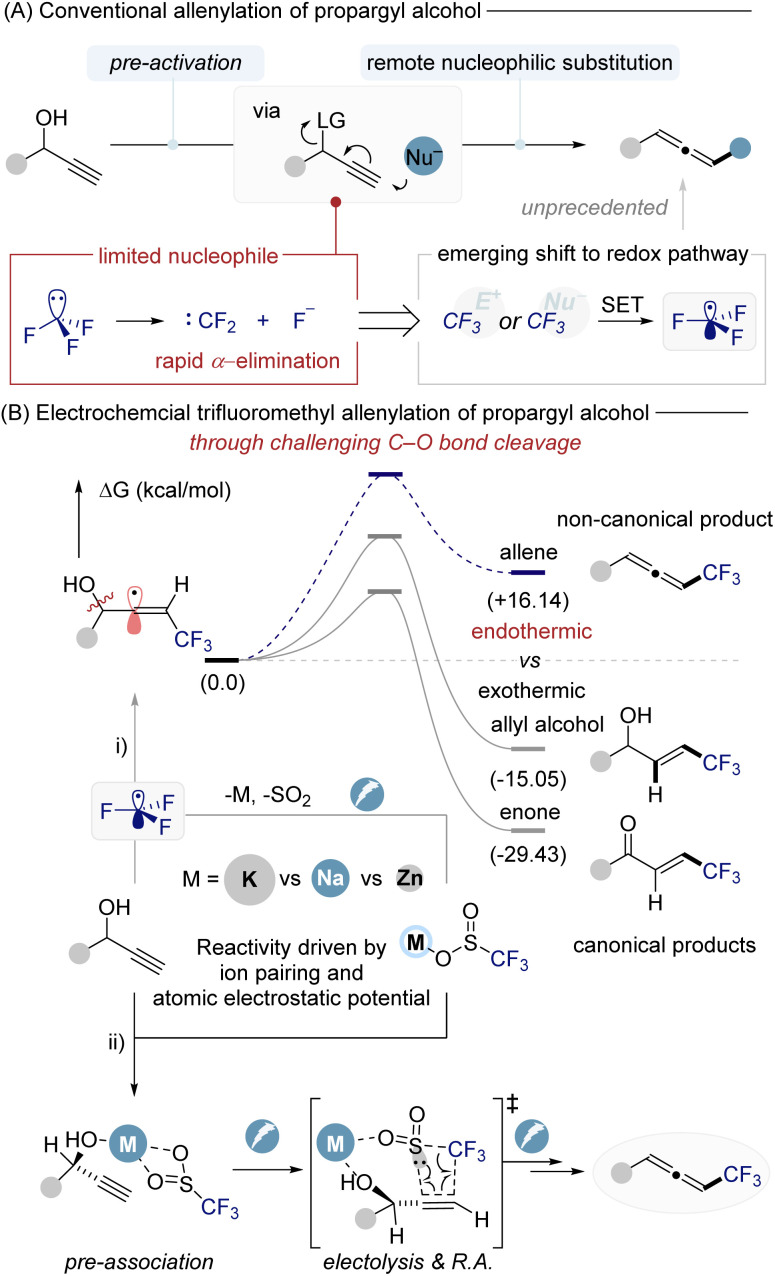
Allenylation of propargyl alcohol.

Propargylic alcohols with both an alcohol and an alkyne are valuable building blocks, enabling diverse bond constructions. A well-established two-electron approach is to convert propargylic alcohols into allenes *via* remote nucleophilic substitution after activating the hydroxyl into a leaving group.^16–40^ This strategy has furnished a variety of functionalized allenes (Fig. 1A).^41–52^ However, the scope of nucleophiles in these transformations remains limited. In particular, a trifluoromethyl anion (CF_3_^−^) cannot be employed in such substitutions because it is intrinsically unstable, undergoing rapid α-elimination to form difluorocarbene and fluoride.^53,54^

This decomposition precludes direct nucleophilic trifluoromethylation of propargylic alcohols.

As a solution, chemists have turned to trifluoromethyl radicals as surrogates for CF_3_^−^, taking advantage of their greater stability and the abundance of reagents available to generate ·CF_3_.^55–57^ Indeed, radical trifluoromethylation has been extensively explored in many contexts, from aromatic C–H functionalization to alkene difunctionalizations, using photochemical, electrochemical, and metal-catalyzed protocols.^58–69^ Surprisingly, however, radical trifluoromethylation of propargylic alcohols to form allenes has not been reported to date. The lack of precedent for this transformation stems from fundamental challenges in radical C–O bond activation (Fig. 1B-i). Selective cleavage of a propargylic C–O bond by radical means is thermodynamically disfavored (endothermic) and must compete against more favorable pathways. For example, addition of a CF_3_ radical to a propargylic alkyne would generate a highly reactive vinyl radical intermediate. Vinyl radicals preferentially follow divergent pathways such as β-scission or hydrogen atom transfer, instead of the productive allene-forming route.^70–75^ Consequently, the intrinsic instability of vinyl radicals, combined with the endothermic nature of C–O bond scission, constitutes a formidable mechanistic barrier to achieving trifluoromethylative allenylation under radical conditions. Herein, we report an electrochemical radical trifluoromethylation of free propargylic alcohols that overcomes these challenges, enabling the first direct synthesis of CF_3_-substituted allenes (Fig. 1B-ii). Central to our design is the *in situ* pre-activation of the alcohol through coordination with a CF_3_ radical source. We envisioned that a reagent such as MSO_2_CF_3_ (metal trifluoromethylsulfinate) could engage in hydrogen bonding or Lewis acid–base coordination with the –OH group, thereby weakening the C–O bond prior to cleavage. Upon electrochemical anodic oxidation, the CF_3_ source generates CF_3_ radicals that attack the alkyne and, crucially, induce C–O bond dissociation to form the allene.

Notably, the coordinated CF_3_–alcohol complex lowers the energy barrier enough to drive what is an endothermic bond cleavage into a productive pathway. The reaction proceeds with high regio- and chemoselectivity, affording trifluoromethylated allenes in good yield. Combined experimental and DFT studies support the formation of the alcohol–CF_3_ complex and its role in guiding the reaction toward allene formation, a transformation that was previously consided infeasible.

## Results and discussion

**Scheme 1:**
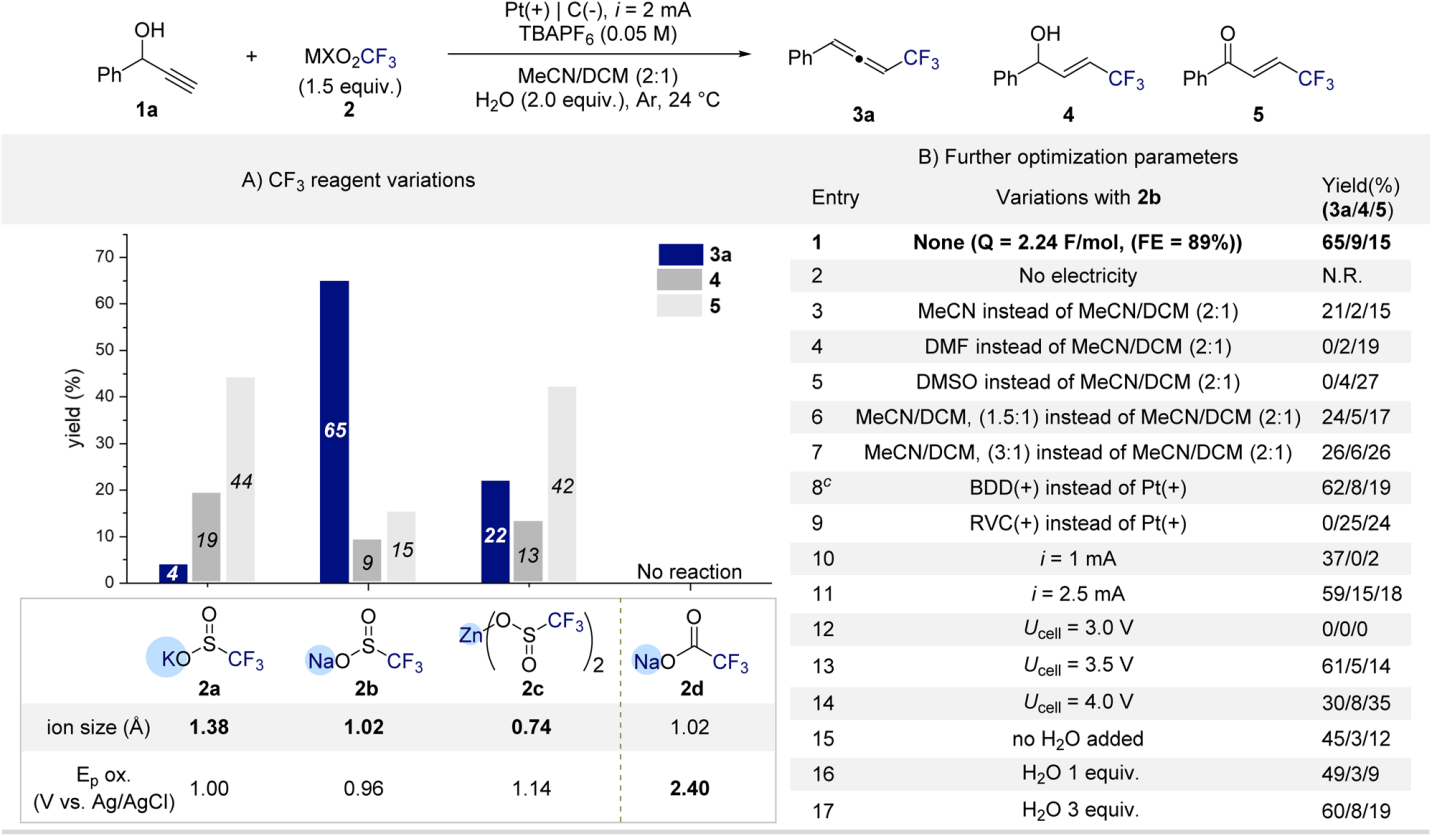
Optimization of reaction parameters.^*a,b a*^Reactions were conducted on 0.2 mmol scale. ^*b*^Yields were determined by ^19^F NMR spectroscopy using 2,2,2-trifluoroethanol as an internal standard.

To prove the critical role of substrate–reagent pre-association in the formation of CF_3_-substituted allenes, we began our investigation by screening various MXO_2_CF_3_ reagents using 1-phenylprop-2-yn-1-ol (1a) as a model substrate (Scheme 1A). Electrolysis was conducted under constant current (2 mA) conditions with *n*Bu_4_NPF_6_ as the electrolyte, a platinum anode, and a graphite cathode in MeCN/DCM (2 : 1) containing H_2_O (2.0 equiv.) as an additive. Gratifyingly, trifluorosulfinate reagents (MSO_2_CF_3_) showed the desired reactivity, affording the CF_3_-allene (3a). The nature of the counter cation proved crucial.

Among Na^+^, K^+^, and Zn^2+^ salts, NaSO_2_CF_3_ (2b) delivered 3a in 65% yield, whereas KSO_2_CF_3_ (2a) and Zn(SO_2_CF_3_)_2_ (2c) primarily generated the competing allylic alcohol (4) or enone (5) byproducts. These results indicate that cation size significantly influences the geometry and strength of the pre-association between the CF_3_ reagent and the alcohol. Notably, the oxidation potentials of 2a–2c differ only slightly, suggesting that the distinct product selectivities arise not from redox differences but from differences in their coordination environments. Taken together, these findings strongly support the formation of a substrate–reagent pre-association complex that governs the selective allene formation. In contrast, the carboxylate analogue NaCO_2_CF_3_ (2d) was completely unreactive under identical conditions, with no detectable conversion of 1a. This inactivity is attributed to its considerably higher oxidation potential, which precludes electrochemical generation of CF_3_ radicals. The divergent reactivity between sulfinates and carboxylates highlights the delicate interplay between reagent redox properties and pre-association effects in enabling endothermic C–O bond activation under electrochemical radical conditions. With NaSO_2_CF_3_ (2b) identified as the optimal CF_3_ source, we next examined the influence of reaction parameters (Scheme 1B). Control experiments confirmed that no product formation occurred in the absence of electrical input, highlighting the essential role of electrolysis in the transformation (entry 2). Solvent effects were pronounced that the use of MeCN, DMF, or DMSO as single solvents or deviation from the 2 : 1 MeCN/DCM ratio led to markedly reduced yields or complete loss of reactivity (entries 3–7). The nature of the anode also proved critical. Replacement of the platinum anode with boron-doped diamond (BDD) maintained comparable efficiency, affording 3a in 61% yield (entry 8). In contrast, the use of a graphite anode completely suppressed allene formation, producing the allylic alcohol (4) and enone (5) byproducts (entry 9). Current intensity exerted a notable influence. Lowering the current to 1 mA resulted in incomplete conversion of 1a (≈60%), while higher currents caused slight decreases in yield, likely due to overoxidation of intermediates (entries 10–11). Constant-potential electrolysis further clarified the relationship between oxidation potential and efficiency. At 3.0 V, no conversion of 1a was observed, consistent with an insufficient anodic potential to initiate oxidation (entry 12). Increasing the potential to 3.5 V restored productive reactivity comparable to that under constant-current conditions (entry 13), whereas potentials above 3.5 V led to significant yield erosion, attributed to oxidative degradation of 3a into overoxidized byproducts such as 5 (entry 14).^76^ Finally, varying the water loading between 1.0 and 3.0 equiv. resulted in only marginal changes in yield (entries 15–17), indicating a subtle yet reproducible influence of water on the overall efficiency.

**Fig. 2:**
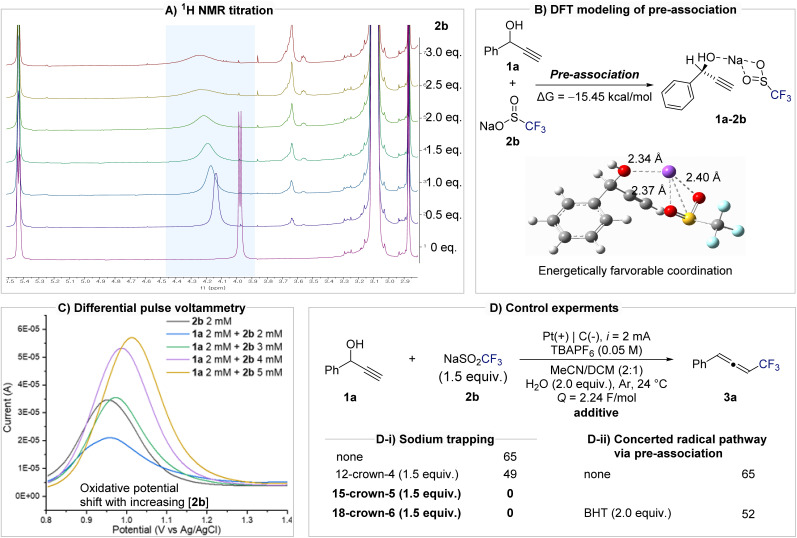
Evidence and implication of sodium cation assisted pre-association in electrochemical allenylation. (A) ^1^H NMR titration experiment: 1a (1.0 equiv.), 2b (*x* equiv.) TBAPF_6_ (1.0 equiv.) H_2_O (2.0 equiv.) in CD_3_CN solvent. (B) DFT calculations were performed at the M062X/def2TZVP/PCM in MeCN level of theory. Free energy given in kcal mol^−1^. (C) Differential pulse voltammetry of 2b, and mixture of 1a with 2b. (D) Results of control experiments.

To elucidate the mechanism and validate the formation of a pre-association complex between 1a and 2b, a series of spectroscopic, electrochemical, and kinetic experiments were conducted (Fig. 2). NMR titration experiments revealed clear evidence of substrate–reagent interaction. Upon incremental addition of 2b to 1a, the O–H peak of 1a shifted progressively downfield and broadened, consistent with coordination between the hydroxyl oxygen and the sodium cation of 2b (Fig. 2A). DFT calculations indicated that the pre-association of 1a and 2b*via* Na^+^ coordination is energetically favorable (Δ*G* = −15.45 kcal mol^−1^; Fig. 2B). Differential pulse voltammetry (DPV) further supported complex formation. The oxidation potential of 2b increased systematically with [2b], suggesting that the redox behavior of 2b is modulated by its associationwith 1a (Fig. 2C). Cyclic voltammetry provided further evidence while individual scans of 1a or 2b showed no significant oxidation waves, their mixture exhibited new redox features. In particular, the oxidation signal of 1a diminished and disappeared, indicating that pre-association between 1a and 2b is mechanistically relevant (see Fig. S2 in the SI). The importance of sodium in mediating this pre-association was confirmed by crown ether experiments (Fig. 2D-i). In the presence of the weak sodium chelator 12-crown-4, the yield of 3a decreased slightly (49%), whereas stronger complexants such as 15-crown-5 and 18-crown-6 completely inhibited product formation. These results clearly demonstrate that sodium acts as an ion-bridge, organizing the propargyl alcohol and CF_3_ source into a reactive complex. To investigate the reaction pathway during the radical addition step, we conducted control experiments using radical inhibitors BHT (butylated hydroxytoluene) and TEMPO (2,2,6,6-tetramethylpiperidine 1-oxyl). Notably, the addition of BHT did not significantly suppress product formation (52% yield), suggesting that free CF_3_ radicals are not involved as discrete intermediates and that the transformation proceeds *via* a concerted radical addition pathway (Fig. 2D-ii). In contrast, in the presence of TEMPO, the reaction predominantly resulted in oxidation of 1a to the corresponding ketone (see Scheme S1 in the SI). This outcome is attributed to the well-established ability of TEMPO to undergo electrochemical oxidation to generate oxyl radical species, which can mediate alcohol oxidation.^77^

**Fig. 3:**
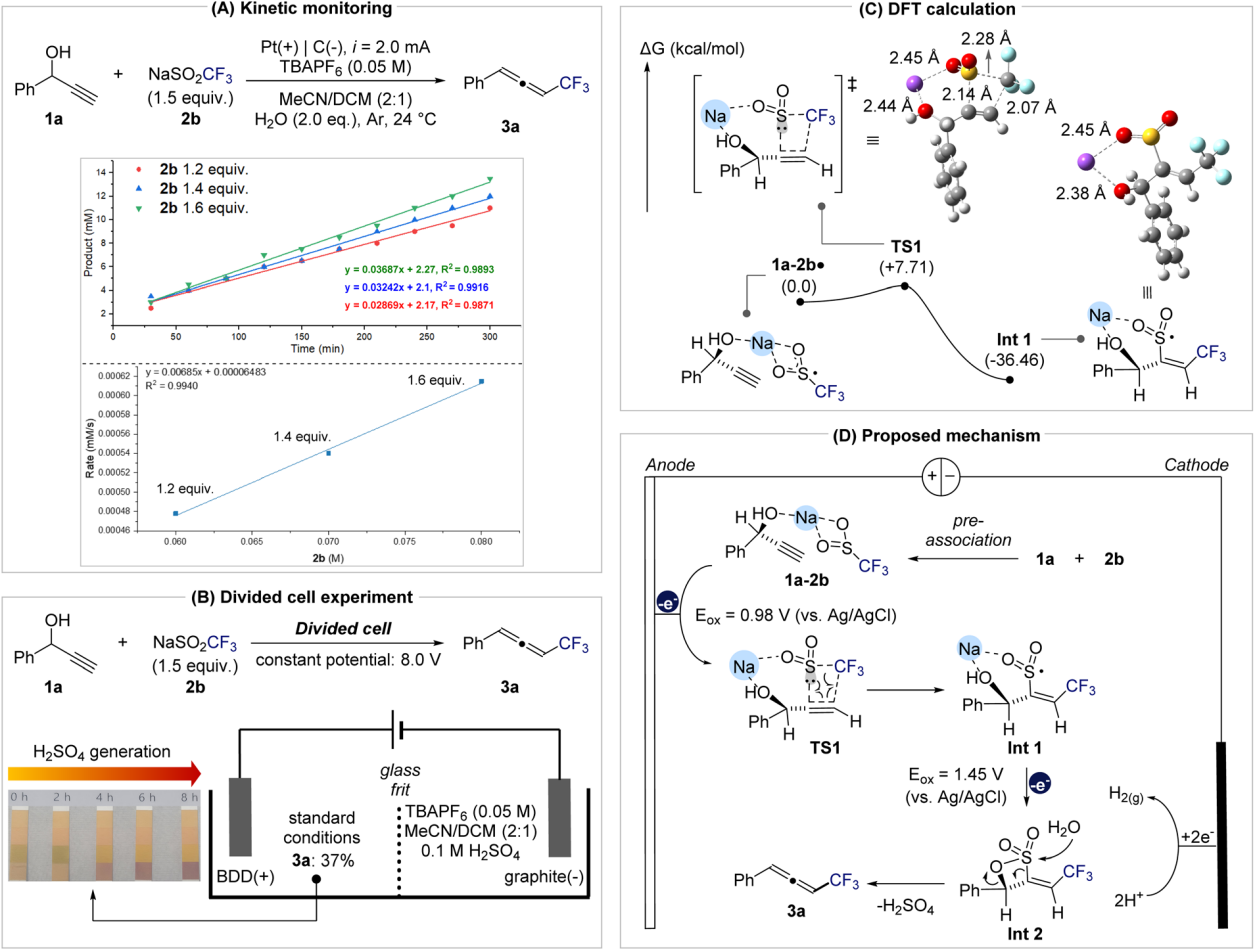
Mechanistic investigations. (A) Variations of 2b's equivalent with standard condition. Reaction monitoring was proceeded by ^19^F NMR using 2,2,2-trfluoroethanol as an internal standard. (B) Result of the divided cell experiment. (C) Computed relative free energies of transition state for concerted radical addition step. DFT calculations were performed at the M062X/def2TZVP/PCM in MeCN level of theory. Free energy given in kcal mol^−1^. (D) Proposed mechanism based on mechanistic investigations.

Notably, the addition of the radical scavenger BHT did not suppress product formation (52% yield), indicating that free CF_3_ radicals are not really involved and that the transformation proceeds *via* a concerted radical addition pathway. Kinetic analysis further clarified the mechanistic relevance of this pre-association (Fig. 3A). Monitoring product accumulation under varying concentrations of 2b while keeping [1a] constant revealed a linear relationship between initial rate and [2b] (*R*^2^ = 0.994), consistent with a first-order dependence on 2b. This result indicates that 2b participates directly in the rate-determining step. A divided-cell experiment confirmed that this transformation originates from anodic processes, as product formation was observed exclusively in the anodic compartment, thereby ruling out cathodic reduction pathways such as vinyl radical formation followed by E1cB-type elimination (Fig. 3B and the SI for further details). In addition, monitoring the pH of the anodic solution revealed a gradual acidification over the course of the reaction, consistent with the generation of H_2_SO_4_. The generated acid does not appear to affect the reactivity of the system. When the isolated product 3a was subjected to H_2_SO_4_ conditions, no decomposition or transformation was observed (Fig. S5 in the SI). Furthermore, under the undivided cell conditions employed in this study, the protons generated at the anode are expected to be reduced at the cathode, thereby completing the redox cycle and preventing the accumulation of acidic species.

To gain deeper insight into the C–O activation step, DFT calculations were performed on the oxidation and radical addition sequence (Fig. 3C). After anodic oxidation of the pre-associated 1a–2b complex, radical addition of CF_3_ to the terminal carbon of the alkyne is facilitated by an ion-bridging interaction between the hydroxyl group and Na^+^, positioning the sulfonyl group for concerted CF_3_/SO_2_ coupling to form intermediate Int-1. Based on these results, a mechanistic model is proposed (Fig. 3D).

The propargylic alcohol (1a) and NaSO_2_CF_3_ (2b) first form a pre-associated complex that undergoes anodic oxidation at 0.98 V *vs.* Ag/AgCl to generate radical species (1a–2b·). This intermediate proceeds through transition state TS1 (Δ*G*^‡^ = +7.71 kcal mol^−1^) to yield Int-1 (Δ*G* = −36.46 kcal mol^−1^) *via* a concerted radical addition. A subsequent anodic oxidation at 1.45 V produces Int-2, which, upon nucleophilic attack of water at the sulfonyl group, releases H_2_SO_4_ and furnishes the trifluoromethylated allene 3a. The H_2_SO_4_ generated in this step provides protons that undergo reduction at the cathode, thereby maintaining charge balance in the electrochemical cell and completing the overall redox cycle.

**Scheme 2:**
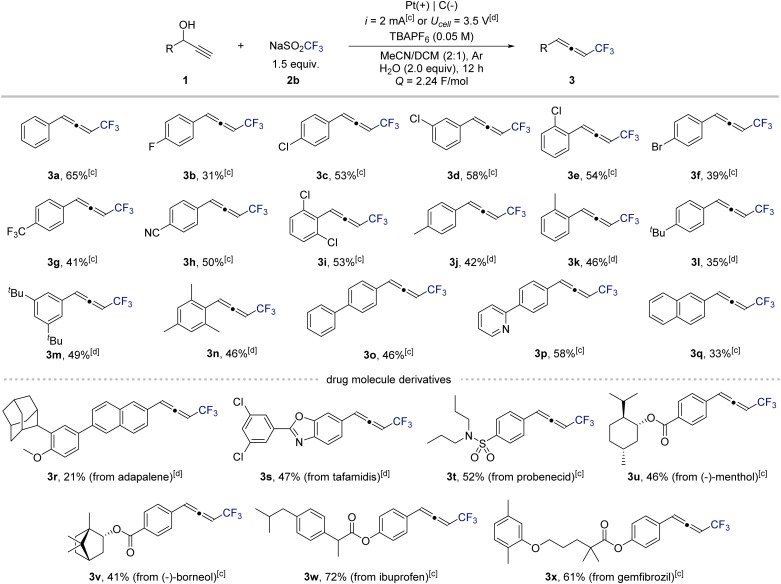
Substrate scope of electrochemical trifluoromethyl allenylation of propargyl alcohols.^*a,b a*^Reactions were conducted on 0.3 mmol scale. ^*b*^Yields were determined by ^19^F NMR using 2,2,2-trifluoroethanol as an internal standard. ^*c*^Reactions were carried out under constant current condition. ^*d*^Reactions were carried out under constant potential condition.

Next, to evaluate the generality of this electrochemical protocol, we examined a diverse range of propargylic alcohols (Scheme 2). Under standard conditions, substrates bearing electron-withdrawing or neutral substituents underwent smooth conversion to the corresponding CF_3_-substituted allenes. In contrast, electron-rich substrates were prone to overoxidation of the allene products under constant-current conditions. Switching to constant-potential electrolysis effectively suppressed this undesired oxidation, ensuring high selectivity (also see Scheme 1B, entry 13). The methodology exhibited broad functional-group tolerance. Halogenated substrates, including fluoro (3b), chloro (3c–3e, 3i), and bromo (3f) derivatives, afforded the desired products in excellent yields. Strongly electron-withdrawing substituents such as trifluoromethyl (3g) and cyano (3h) groups were also well tolerated. For electron-donating substituents (3j–3n), the corresponding allenes were obtained in high yields under the modified constant-potential conditions. Substrates containing extended π-systems or heteroaromatic motifs, including biphenyl (3o), pyridyl (3p), and naphthyl (3q), participated readily, highlighting the compatibility of this method with conjugated frameworks. To further demonstrate synthetic utility, late-stage trifluoromethylation of complex molecules was explored. Natural-product-derived and drug-like scaffolds such as adapalene (3r), tafamidis (3s), and a probenecid analogue (3t) underwent smooth transformation to their corresponding CF_3_-allenes. Similarly, benzoate- and acetoxy-functionalized natural products including menthol (3u) and (+)-borneol (3v) reacted efficiently. Widely used pharmaceuticals such as ibuprofen (3w) and gemfibrozil (3x) also underwent selective conversion.

**Scheme 3:**
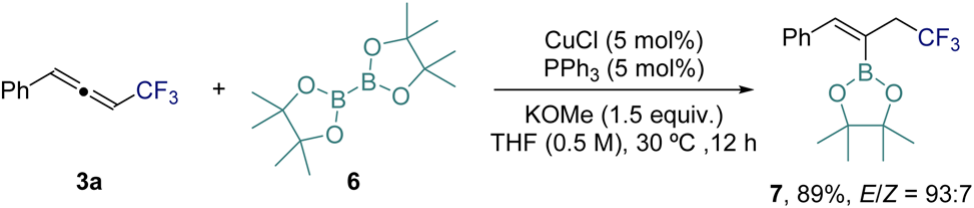
Application of CF_3_-allene 3a: Cu-catalyzed hydroboration.^78^

As highlighted in the introduction, CF_3_-allenes can serve as versatile building blocks for further transformations. To demonstrate this potential, we investigated the hydroboration of 3a using bis(pinacolato)diborane (6) under copper catalysis (Scheme 3). Notably, this transformation provides an alkenyl boronate bearing an allylic CF_3_ moiety (7), which differs from products reported under related copper-catalyzed hydroboration conditions.^78^ This result highlights the potentially unique reactivity of CF_3_-substituted allenes.

## Conclusions

In summary, we have developed an electrochemical strategy for the selective conversion of free propargylic alcohols into CF_3_- substituted allenes through sodium-mediated radical C–O bond activation. This transformation achieves a formally endothermic C–O bond cleavage under mild electrochemical conditions by exploiting a pre-association mechanism between the substrate and the CF_3_ source. Systematic mechanistic investigations, combining NMR, electrochemical, kinetic, and DFT analyses, revealed that the Na^+^ ion serves as an ion-bridge, preorganizing the propargyl alcohol and CF_3_ reagent into a reactive complex that undergoes concerted radical addition upon anodic oxidation. This method exhibits broad substrate scope, high chemoselectivity, and excellent functional-group tolerance, enabling late-stage trifluoromethylation of complex molecules. Beyond providing a synthetically practical route to CF_3_-substituted allenes, this work establishes a general strategy for activating strong C–O bonds *via* reagent–substrate preorganization, expanding the conceptual landscape of electrochemical radical chemistry.

## Author contributions

J. J. performed synthetic and mechanistic studies. H. K. and E. J. C. coordinated the experiments and analyses. J. J. and E. J. C. analyzed the experimental data and wrote the manuscript.

## Conflicts of interest

There are no conflicts to declare.

## Supplementary Material

SC-OLF-D6SC02203K-s001

## Data Availability

The data underlying this study are available in the published article and its supplementary information (SI). Supplementary information: experimental procedures, electroanalytical studies, computational studies, characterization of synthesized compounds, and spectroscopic data. See DOI: https://doi.org/10.1039/d6sc02203k.

## References

[cit1] Hendon C. H., Murray A. T., Carbery D. R., Walsh A. R. (2013). Chem. Sci..

[cit2] Palmer M. H. (2006). Chem. Phys..

[cit3] Soriano E., Fernández I. (2014). Chem. Soc. Rev..

[cit4] Han X.-L., Lin P.-P., Li Q. (2019). Chin. Chem. Lett..

[cit5] Yang B., Qiu Y., Bäckvall J.-E. (2018). Acc. Chem. Res..

[cit6] Qiu G., Zhang J., Zhou K., Wu J. (2018). Tetrahedron.

[cit7] Adams C. S., Weatherly C. D., Burke E. G., Schomaker J. M. (2014). Chem. Soc. Rev..

[cit8] Ma S. (2005). Chem. Rev..

[cit9] Singh J., Sharma A., Sharma A. (2021). Org. Front. Chem..

[cit10] Rivera-Fuentes P., Diederich F. (2012). Angew. Chem., Int. Ed..

[cit11] Hoffmann-Röder A., Krause N. (2004). Angew. Chem., Int. Ed..

[cit12] Meanwell N. A. (2018). J. Med. Chem..

[cit13] Wang J., Sánchez-Roselló M., Aceña J. L., Pozo C., Sorochinsky A. E., Fustero S., Soloshonok V. A., Liu H. (2014). Chem. Rev..

[cit14] Purser S., Moore P. R., Swallow S., Gouverner V. (2008). Chem. Soc. Rev..

[cit15] Müller L., Faeh C., Diederich F. (2007). Science.

[cit16] Chao J., Yang R., Huang J., Chen X., Song X.-R., Xiao Q. (2025). Org. Biomol. Chem..

[cit17] Negesh K., Reddy N. S., Kareem S., Vali S. R., Reddy B. V. S. (2025). Org. Biomol. Chem..

[cit18] Kim S., Lee D. S., Iqbal N., Bae J., Hwang H. S., Baek D., Hong S., Cho E. J. (2024). Chem Catal..

[cit19] Mishra S., Raikwar S., Baire B. (2023). Chem.–Asian J..

[cit20] Doraghi F., Mahdavian A. M., Karimian S., Larijiani B., Mahdavi M. (2023). Adv. Synth. Catal..

[cit21] Jiang S., Ma H., Yang R., Song X.-R., Xiao Q. (2022). Org. Chem. Front..

[cit22] Xiaozheng Z., Qinqin L., Guiyan C., Xiaolong Z., Yingpeng S. (2022). Chin. J. Org. Chem..

[cit23] Wu J., Guo Q., Hong H., Xie R., Zhu N. (2022). J. CO_2_ Util..

[cit24] Liu X.-Y., Liu Y.-L., Chen L. (2020). Adv. Synth. Catal..

[cit25] Wang Z., Lin X., Chen X., Li P., Li W. (2021). Org. Chem. Front..

[cit26] Zhu W.-R., Su Q., Diao H.-J., Wang E.-X., Wu F., Zhao Y.-L., Weng J., Lu G. (2020). Org. Lett..

[cit27] Kumar G. R., Rajesh M., Lin S., Liu S. (2020). Adv. Synth. Catal..

[cit28] Qian H., Huang D., Bi Y., Yan G. (2019). Adv. Synth. Catal..

[cit29] Khan T., Yaragorla S. (2019). Eur. J. Org Chem..

[cit30] Noël F., Vuković V. D., Yi J., Richmond E., Kravljanac P., Moran J. (2019). J. Org. Chem..

[cit31] Boreux A., Lonca G. H., Riant O., Gagosz F. (2016). Org. Lett..

[cit32] Ambler B. R., Peddi S., Altman R. A. (2015). Org. Lett..

[cit33] Ji Y.-L., Kong J.-J., Lin J.-H., Xiao J.-C., C Gu Y. (2014). Org. Biomol. Chem..

[cit34] Zhao T. S. N., Szabó K. J. (2012). Org. Lett..

[cit35] Yamazaki T., Watanabe Y., Yoshida N., Kawasaki-Takasaka T. (2012). Tetrahedron.

[cit36] Li P., Liu Z.-J., Liu J.-T. (2010). Tetrahedron.

[cit37] Watanabe Y., Yamazaki T. (2009). Synlett.

[cit38] Shimizu M., Higashi M., Takeda Y., Jiang G., Murai M., Hiyama T. (2007). Synlett.

[cit39] Yamazaki T., Yamamoto T., Ichihara R. (2006). J. Org. Chem..

[cit40] Sakamoto T., Takahashi K., Yamazaki T., Kitazume T. (1999). J. Org. Chem..

[cit41] Beluze C., Hu T., Bouyssi D., Monteiro N., Amgoune A. (2025). Synthesis.

[cit42] Xiao B.-Y., Huang W., Zhang F.-H. (2025). Eur. J. Org Chem..

[cit43] Gan L., Wan X., Pang Y., Zou Y., Deng Y.-H., Shao Z. (2025). Org. Chem. Front..

[cit44] Lin R., Zhang Y., Xiong T., Huang H., Peng X., Zhang Y., Huang N., Hu W., Jiang J. (2025). ACS Catal..

[cit45] Yaragorla S., Shaik A., Latha D. S. (2025). J. Org. Chem..

[cit46] Chen Z., Qian H. (2025). Org. Lett..

[cit47] Chang H., Wang R., Wang Y.-M. (2025). Chem.–Asian J..

[cit48] Xia Y., Liu M., Li W., Li P. (2024). Asian J. Org. Chem..

[cit49] Jarava-Barrera C., Parra A., Quesada S., Orgaz-Gordillo S., Pradilla R. F., Viso A., Teresa J., Alonso I., Tortosa M. (2024). Adv. Synth. Catal..

[cit50] Xiao W., Wu J. (2022). Org. Chem. Front..

[cit51] Du S., Zhou A.-X., Yang R., Song X.-R., Xiao Q. (2021). Org. Chem. Front..

[cit52] Wu S., Huang X., Fu C., Ma S. (2017). Org. Chem. Front..

[cit53] Fujihira Y., Liang Y., Ono M., Hirano K., Kagawa T., Shibata N. (2021). Beilstein J. Org. Chem..

[cit54] Saito T., Wang J., Tokunaga E., Tsuzuki S., Shibata N. (2018). Sci. Rep..

[cit55] Giri R., Fernandes A. J., Katayev D. (2025). Acc. Chem. Res..

[cit56] Duan M., Shao Q., Zhou Q., Baran P. S., Houk K. N. (2024). Nat. Commun..

[cit57] Xiao H., Zhang Z., Fang Y., Zhu L., Li C. (2021). Chem. Soc. Rev..

[cit58] Kim S., Kim H. (2025). ACS Catal..

[cit59] Shaw R., Sihag N., Bhartiya H., Yadav M. R. (2024). Org. Chem. Front..

[cit60] Kim S., Kim H. (2024). J. Am. Chem. Soc..

[cit61] Ouyang Y., Qing F.-L. (2024). J. Org. Chem..

[cit62] Ka C. H., Kim S., Cho E. J. (2023). Chem. Rec..

[cit63] Zou Z., Zhang W., Wang Y., Pan Y. (2021). Org. Chem. Front..

[cit64] Bhaskaran R. P., Babu B. P. (2020). Adv. Synth. Catal..

[cit65] Ye F., Berger F., Jia H., Ford J., Wortman A., Börgel J., Genicot C., Ritter T. (2019). Angew. Chem., Int. Ed..

[cit66] Oh E. H., Kim H. J., Han S. B. (2018). Synthesis.

[cit67] Chatterjee T., Iqbal N., You Y., Cho E. J. (2016). Acc. Chem. Res..

[cit68] Cho E. J. (2016). Chem. Rec..

[cit69] Ni C., Hu M., Hu J. (2015). Chem. Rev..

[cit70] Tang N., Chen S., Li C., Du P., Yang W., Qiu R. (2025). ACS Catal..

[cit71] Liang M., Ma H., Song X.-R., Xiao Q. (2024). Adv. Synth. Catal..

[cit72] Bovonsombat P., Sophanpanichkul P., Losuwanakul S. (2022). RSC Adv..

[cit73] Zhang B., Wang T. (2018). Asian J. Org. Chem..

[cit74] Kumar R. K., Bi X. (2016). Chem. Commun..

[cit75] Park S., Joo J. M., Cho E. J. (2015). Eur. J. Org Chem..

[cit76] Lee K. S., Barbieri F., Casali E., Marris E. T., Zanoni G., Schomaker J. M. (2025). J. Am. Chem. Soc..

[cit77] Kawajiri T., Hosoya M., Goda S., Sato E., Suga S. (2025). Org. Lett..

[cit78] Akiyama S., Nomura S., Kubota K., Ito H. (2020). J. Org. Chem..

