# Supplementary material for: Electrochemical deoxygenative trifluoromethylallenylation of propargylic alcohols via sodium-mediated pre-association
Source: Chem Sci. 2026 Apr 28;17(23):11488–95. doi: 10.1039/d6sc02203k (PMC13142707; doi:10.1039/d6sc02203k)

Supplementary Information (SI) for Chemical Science.
This journal is © The Royal Society of Chemistry 2026

Electrochemical Deoxygenative Trifluoromethylallenylation of Propargylic Alcohols via Sodium-Mediated Pre-association

Jihoon Jang[1], Hyunwoo Kim[2] and Eun Jin Cho[1,*]

[1]Department of Chemistry, Chung-Ang University, 84 Heukseok-ro, Dongjak-gu, Seoul 06974, Republic of Korea

[2]Department of Chemistry, Pohang University of Science and Technology (POSETECH), Pohang 37673, Republic of Korea

E-mail: ejcho@cau.ac.kr (E. J. Cho)

Supporting Information

General Considerations S-1

Experimental Details S-2

Mechanistic Experiments S-6

Details of DFT Studies S-11

Analytic Data for Synthesized Compounds S-14

References S-23

NMR Spectra (^{1}H NMR, ^{13}C NMR, and ^{19}F NMR) S-24

General Considerations

General Reagent Information

All reagents required for the synthesis of CF_3-allenes **3** were purchased from Sigma-Aldrich, Alfa Aesar, Acros Organic, or TCI chemical companies. Sodium trifluoromethanesulfinate ($NaSO_2CF_3$) was purchased from BLD pharm. Flash column chromatography was performed using ZEOCHEM ZEOprep silica gel 60 (60-200 mesh). Electrochemical reactions were performed using mkc3405 power supply from MK power company or ElectraSyn 2.0 with accessories including vials and electrodes from IKA. Graphite electrode (10 x 20 mm, thickness 2 mm) was purchased from Qingdao-Baofeng graphite company and platinum electrode (10 x 20 mm, thickness 0.05 mm) was purchased from Thermo Fisher Scientific. After each reaction, the graphite electrode surface was washed with acetone/ethanol three times and sonicated using acetone/distilled water for 10 minutes.

General Analytical Information

The synthesized CF_3-allenes **3** were characterized by ^{1}H NMR, ^{13}C NMR, ^{19}F NMR, and mass spectrometry. NMR spectra were recorded on a Bruker 400 MHz Avance-Core instrument (400 MHz for ^{1}H NMR, 101 MHz for ^{13}C NMR and 376 MHz for ^{19}F NMR). ^{1}H NMR experiments are reported in units, parts per million (ppm), and were measured relative to residual chloroform (7.26 ppm) in the deuterated solvent. ^{13}C NMR spectra are reported in ppm relative to chloroform-D (77.23 ppm), and all were obtained with ^{1}H decoupling. The coupling constants were reported in hertz (Hz) and the multiplicities were denoted using abbreviations, such as: s for singlet, d for doublet, t for triplet, q for quartet, dd for doublet of doublets, dt for doublet of triplets, dq for doublet of quartet, qq for quartet of quartet, and m for multiplet. Reactions were monitored by thin layer chromatography (TLC) or GC-MS of the crude reaction mixture using *n*-dodecane as the internal standard and products were detected by GC-MS using the Agilent GC 7890B/5977A inert MSD with Triple-Axis Detector. Mass spectral data of all unknown compounds were acquired at the Korea Basic Science Institute (Daegu) on a Jeol JMS 700 high-resolution mass spectrometer. A quadrupole mass analyzer was used for HRMS measurements. For cyclic voltammetry measurement, CHI620E potentiostat (CH Instruments) was used.

Experimental Details

Procedure for preparation of 1b – 1q[S-1]

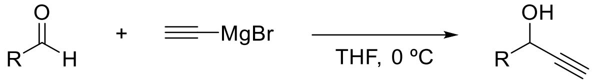

An oven-dried 30 mL reaction tube equipped with a magnetic stir bar was charged with the aldehyde (5 mmol, 1 eq.) and THF (10 mL). Cool the solution to 0 °C. Then, add ethynylmagnesium bromide (0.5 M in THF, 1.2 eq.) to the solution. After 3 h, saturated aqueous NH_4Cl was added to the reaction mixture, and the mixture was diluted with EtOAc and washed with brine. The organic layer was dried over anhydrous $MgSO_4$, filtered, and concentrated in vacuo. The crude product was purified by flash column chromatography using hexanes/EtOAc as the eluent to afford the corresponding products.

Procedure for preparation of 1r – 1t[S-2]

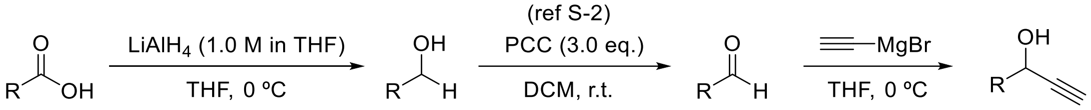

Step 1: An oven-dried 100 mL round bottom flask equipped with a magnetic stir bar was charged with the acid (5 mmol, 1 eq.) and THF (10 mL) under argon condition. Cool the solution to 0 °C. Then, dropwise lithium aluminum hydride (1.0 M in THF, 2.4 eq.) to the solution. After 1 h, saturated aqueous NH_4Cl was added to the reaction mixture, and the mixture was diluted with EtOAc and washed with distilled water. The organic layer was dried over anhydrous Na_2SO_4, filtered, and concentrated in vacuo. The crude product was purified by flash column chromatography using hexanes/EtOAc as the eluent to afford the corresponding products.

Step 2: An oven-dried 30 mL reaction tube equipped with a magnetic stir bar was charged with pyridinium chlorochromate (6 mmol, 1.2 eq.), alcohol (5 mmol, 1.0 eq.) and dry DCM (10 mL) under argon condition. Then, stir the reaction at room temperature for 3 h. After 3 h, filter the suspension and wash the resulting residue with ethyl acetate. Concentrate the filtrate in vacuo. The crude product was purified by flash column chromatography using hexanes/EtOAc as the eluent to afford the corresponding products.

Step 3: An oven-dried 30 mL reaction tube equipped with a magnetic stir bar was charged with the aldehyde (5 mmol, 1 eq.) and THF (10 mL). Cool the solution to 0 °C. Then, add ethynylmagnesium bromide (0.5 M in THF, 1.2 eq.) to the solution. After 3 h, saturated

aqueous NH_4Cl was added to the reaction mixture, and the mixture was diluted with EtOAc and washed with brine. The organic layer was dried over anhydrous $MgSO_4$, filtered, and concentrated in vacuo. The crude product was purified by flash column chromatography using hexanes/EtOAc as the eluent to afford the corresponding products.

Procedure for preparation of 1u – 1x[S-3]

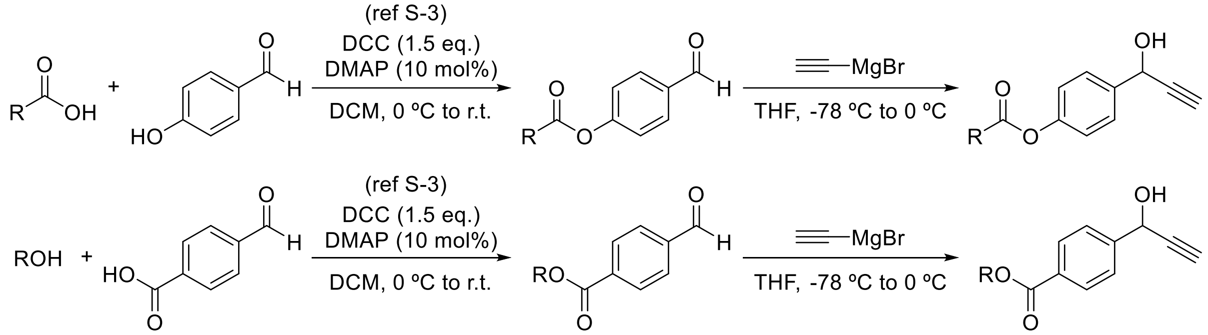

Step 1: An oven-dried 100 mL round bottom flask equipped with a magnetic stir bar was charged with the 4-dimethylaminopyridine (0.5 mmol, 10 mol%), acid (5.0 mmol, 1.0 eq.), alcohol (5.0 mmol, 1.0 eq.), dicyclohexylcarbodiimide (7.5 mmol, 1.5 eq.) and DCM (20 mL) under 0 °C, argon condition. After 12 h, filter the mixture and concentrate the filtrate in vacuo. The crude product was purified by flash column chromatography using hexanes/EtOAc as the eluent to afford the corresponding products.

Step 2: An oven-dried 30 mL reaction tube equipped with a magnetic stir bar was charged with the aldehyde (5 mmol, 1 eq.) and THF (10 mL). Cool the solution to -78 °C. Then, add ethynylmagnesium bromide (0.5 M in THF, 1.2 eq.) to the solution over an hour. After all the solution added, the mixture is stirred at 0 °C for 12 hours. After 12 h, saturated aqueous NH_4Cl was added to the reaction mixture, and the mixture was diluted with EtOAc and washed with brine. The organic layer was dried over anhydrous Na_2SO_4, filtered, and concentrated in vacuo. The crude product was purified by flash column chromatography using hexanes/EtOAc as the eluent to afford the corresponding products.

Method 1. Procedure for Electrochemical Synthesis of CF_3-allenes (Constant Current)

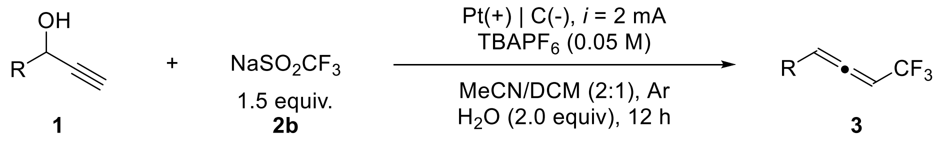

A reaction tube equipped with stirring bar was charged with propargyl alcohol derivative **1** (0.3 mmol), sodium trifluoromethanesulfinate **2b** (70 mg, 0.45 mmol), $TBAPF_6$ (117 mg, 0.3 mmol), distilled water (11 μL, 0.6 mmol) in MeCN/DCM (4 mL/2 mL, total 6 mL). The reaction tube was sealed with a silicon septum screw cap and then purged with argon gas using a balloon for 10 minutes. A two-electrode setup with platinum foil anode (surface area = 1 × 1 cm^2) and graphite plate (surface area = 1 × 1 × 0.2 cm^3) were used. The constant current electrolysis was performed at 2 mA. The reaction progress was monitored using TLC and/or fluorine NMR (^{19}F NMR). After completion, the mixture was diluted with dichloromethane (20 mL) and washed with brine. The combined organic layer was dried over $MgSO_4$, filtered, and the filtrate was concentrated under reduced pressure. The resultant residue was purified by flash column chromatography to yield the corresponding CF_3-allenes **3**.

Method 2. Procedure for Electrochemical Synthesis of CF_3-allenes (Constant Potential)

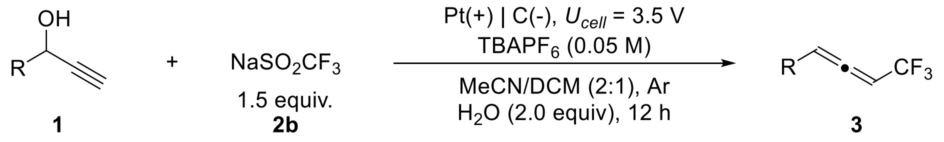

A reaction tube equipped with stirring bar was charged with propargyl alcohol derivative **1** (0.3 mmol), sodium trifluoromethanesulfinate **2b** (70 mg, 0.45 mmol), $TBAPF_6$ (117 mg, 0.3 mmol), distilled water (11 μL, 0.6 mmol) in MeCN/DCM (4 mL/2 mL, total 6 mL). The reaction tube was sealed with a silicon septum screw cap and then purged with argon gas using a balloon for 10 minutes. A two-electrode setup with platinum foil anode (surface area = 1 × 1 cm^2) and graphite plate (surface area = 1 × 1 × 0.2 cm^3) were used. The constant potentail electrolysis was performed at 3.5 V. The reaction progress was monitored using TLC and/or fluorine NMR (^{19}F NMR). After completion, the mixture was diluted with dichloromethane (20 mL) and washed with brine. The combined organic layer was dried over MgSO4, filtered, and the filtrate was concentrated under reduced pressure. The resultant residue was purified by flash column chromatography to yield the corresponding CF_3-allenes **3**.

Photographs of Experiment Setup for Electrochemical Reaction (Power Supply)

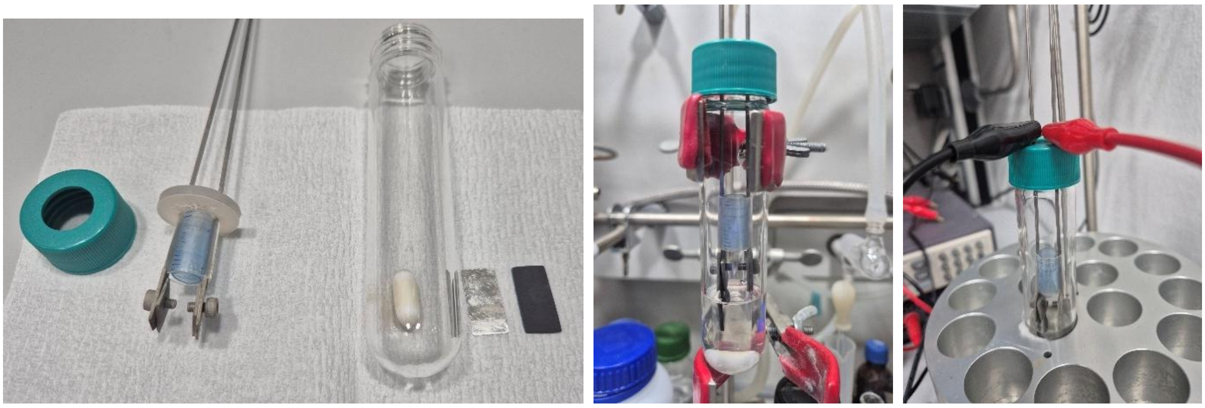

Photographs of Experiment Setup for Electrochemical Reaction (ElectraSyn)

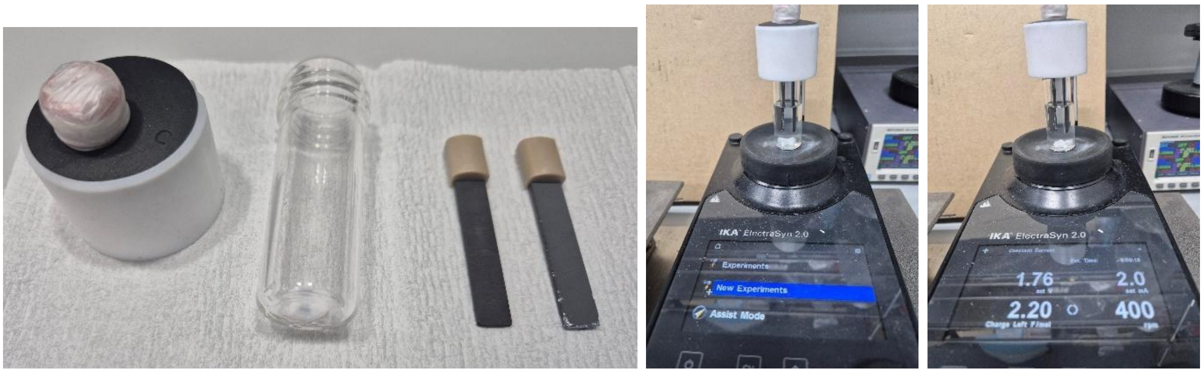

Mechanistic Experiments

Kinetic Experiments[a,b,c]

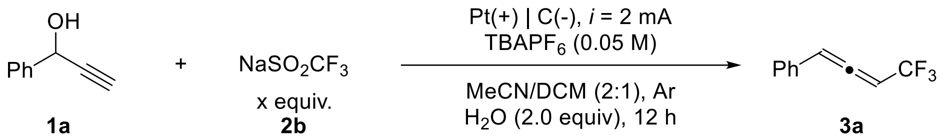

time (min)	1.2 equiv.	1.4 equiv.	1.5 equiv.	1.6 equiv.
30	5	7	6	7
60	8	8	9	10
90	10	10	10	11
120	12	12	14	13
150	13	13	15	15
180	15	15	17	16
210	16	18	19	18
240	18	20	22	23
270	19	22	24	23
300	22	24	27	27

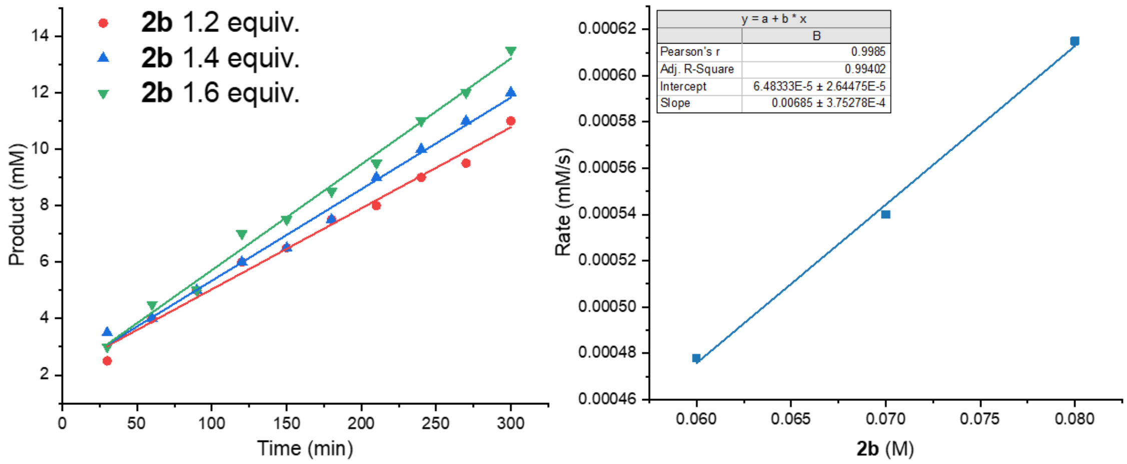

Figure S1. [a]0.3 mmol scale; [b]The reactions followed by **method 1**; [c]Yields were determined by ^{19}F NMR using 2,2,2-trifluoroethanol as an internal standard.

Cyclic Voltammetry Experiments

The ground-state oxidation potential (E_{ox}) of 1-phenylprop-2-yn-1-ol **1a**, sodium trifluoromathanesulfinate **2b** ($NaSO_2CF_3$) and mixtures were determined by cyclic voltammetry. Samples were dissolved at a concentration of 5 mM in 5 mL of degassed acetonitrile (MeCN) containing 0.10 M tetrabutylammonium hexafluorophosphate ($TBAPF_6$). A three-elecrode cell assembly consisting of a glassy carbon (GC) working electrode, a Pt coiled counter electrode, and Ag/AgCl pseudo reference electrode was employed for the voltammetric measurements. Voltammograms were measured at a scan rate of 50 mV s^{-1}. Oxidations were measured by scanning potentials in positive direction. Data was analyzed by using Origin pro software. All resulted redox potentials were calibrated using ferrocene redox couple (0.40 V vs. Ag/AgCl).

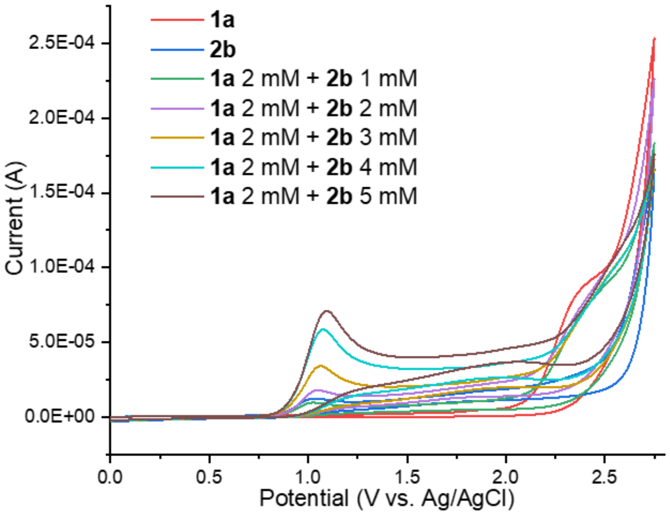

Figure S2. Pre-association Supported by CV experiments

Differential Pulse Voltammetry Experiments

The ground-state oxidation potential (E_{ox}) of sodium trifluoromathanesulfinate **2b** ($NaSO_2CF_3$) and mixtures were determined by differential pulse voltammetry experiments. Samples were dissolved at a concentration of 5 mM in 5 mL of degassed acetonitrile (MeCN) containing 0.10 M tetrabutylammonium hexafluorophosphate ($TBAPF_6$). A three-elecrode cell assembly consisting of a glassy carbon (GC) working electrode, a Pt coiled counter electrode, and Ag/AgCl pseudo reference electrode was employed for the voltammetric measurements. Voltammograms were measured at a pulse width of 5 ms, sampling width 1.67 ms and pulse period 0.5 s. Oxidations were measured by scanning potentials in positive direction. Data was

analyzed by using Origin pro software. All resulted redox potentials were calibrated using ferrocene redox couple (0.40 V vs. Ag/AgCl).

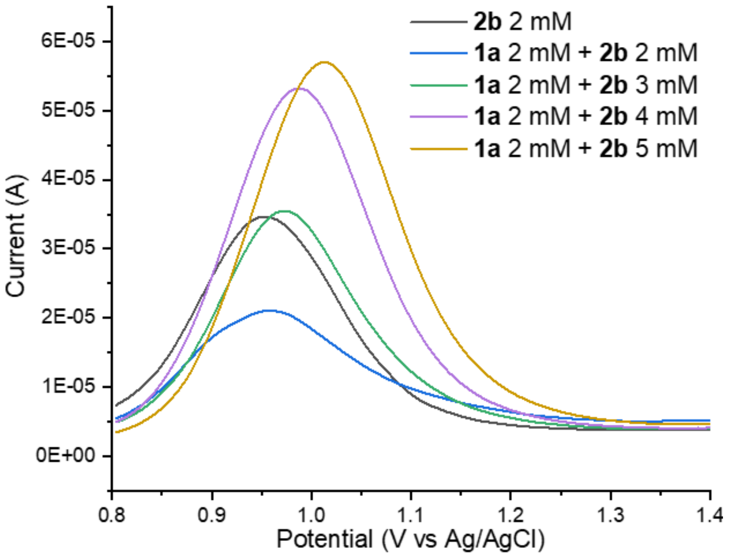

Figure S3. Pre-association Supported by DPV experiments

Reaction in the presence of TEMPO

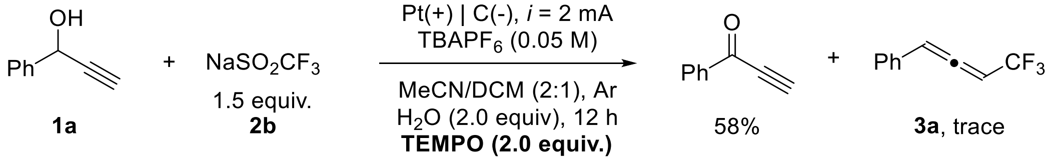

Scheme S1. Reaction in the presence of TEMPO

A reaction tube equipped with stirring bar was charged with propargyl alcohol derivative **1** (0.3 mmol), sodium trifluoromethanesulfinate **2b** (70 mg, 0.45 mmol), $TBAPF_6$ (117 mg, 0.3 mmol), distilled water (11 µL, 0.6 mmol) and TEMPO (93.8 mg, 0.6 mmol) in MeCN/DCM (4 mL/2 mL, total 6 mL). The reaction tube was sealed with a silicon septum screw cap and then purged with argon gas using a balloon for 10 minutes. A two-electrode setup with platinum foil anode (surface area = 1 × 1 cm^2) and graphite plate (surface area = 1 × 1 × 0.2 cm^3) were used. The constant current electrolysis was performed at 2 mA. The reaction progress was monitored using TLC, proton NMR (^1H NMR), fluorine NMR (^{19}F NMR) and GC-MS.

Electrochemical Synthesis of CF_3-allene Using Divided Cell

The electrolysis was carried out using IKA Electrasyn 2.0 in H-type divided cell (IKA Pro-Divide; purchased from IKA) equipped magnetic stirrer bars on anodic chamber and cathodic chamber. Boron-doped diamond electrode (purchased from IKA) was used as anode and

graphite plate electrode (purchased from IKA) cathode. The anodic chamber was charged 1-phenylprop-2-yn-1-ol **1a** (0.3 mmol), sodium trifluoromethanesulfinate **2b** (0.45 mmol), and $TBAPF_6$ (0.3 mmol) distilled water (11 µL, 0.6 mmol) in MeCN/DCM (4 mL/2 mL, total 6 mL). The cathodic chamber was charged sulfuric acid (0.1 M) and $TBAPF_6$ (0.3 mmol) in MeCN (6 mL). The electrolysis was performed constant potential at 8.0 V. The reaction progress was monitored using TLC and ^{19}F NMR. After completion, the working electrode cell reaction mixture was diluted with dichloromethane (20 mL) and washed with distilled water. The combined organic layer was dried with $MgSO_4$, filtered, and the filtrate was concentrated under reduced pressure. The resultant residue was purified by flash column chromatography to yield the corresponding CF_3-allene **3a**.

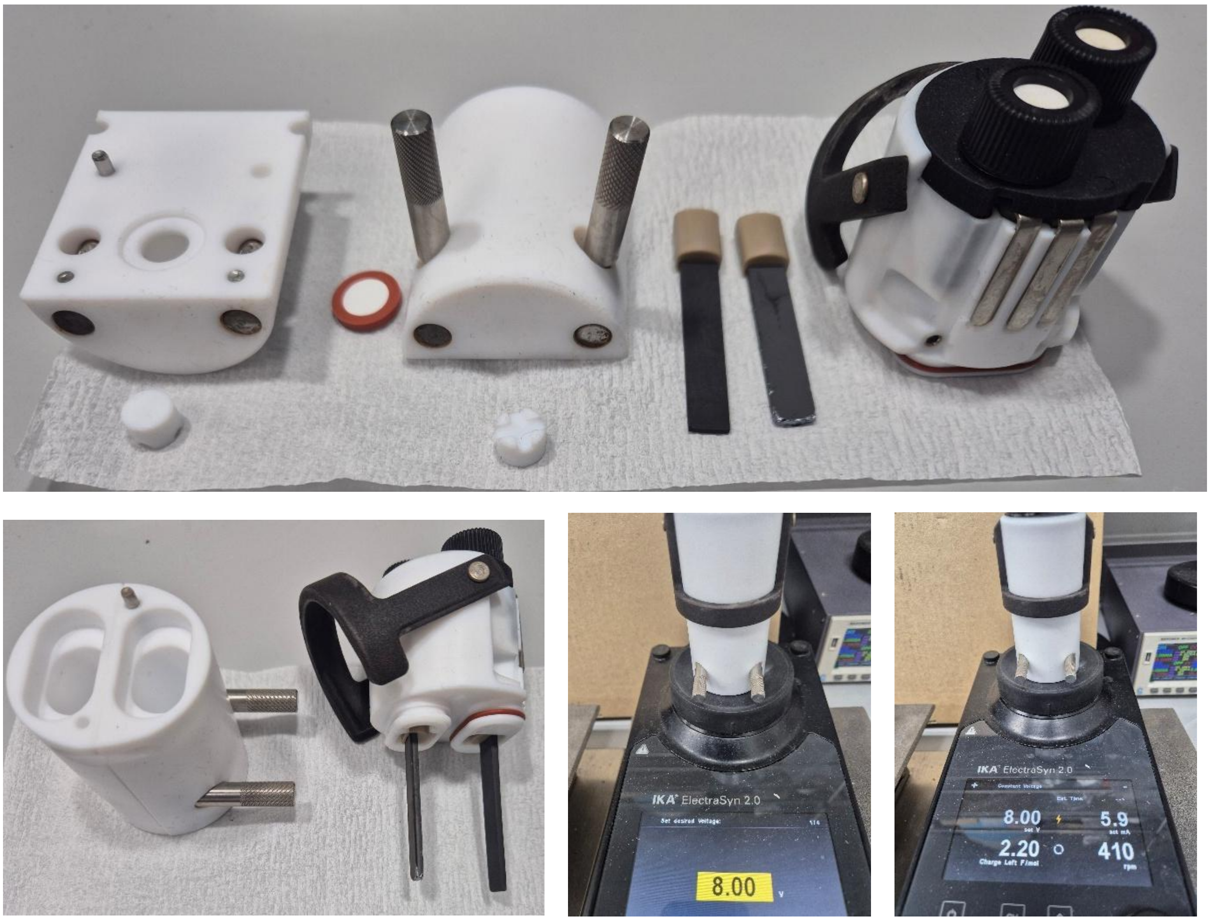

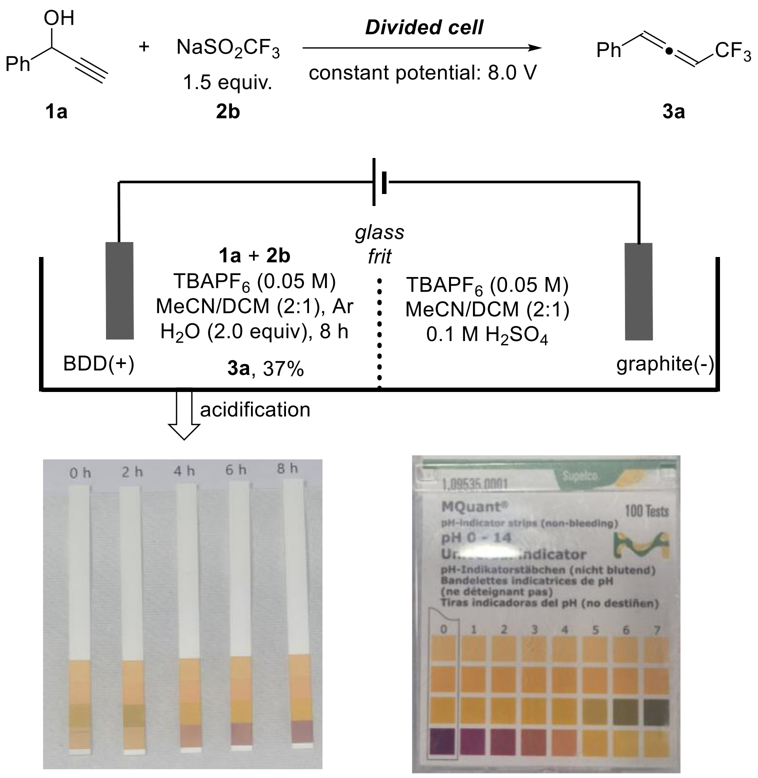

Figure S4. Result of divided cell experiment

Investigation of the Effect of H_2SO_4 on the 3a Transformation

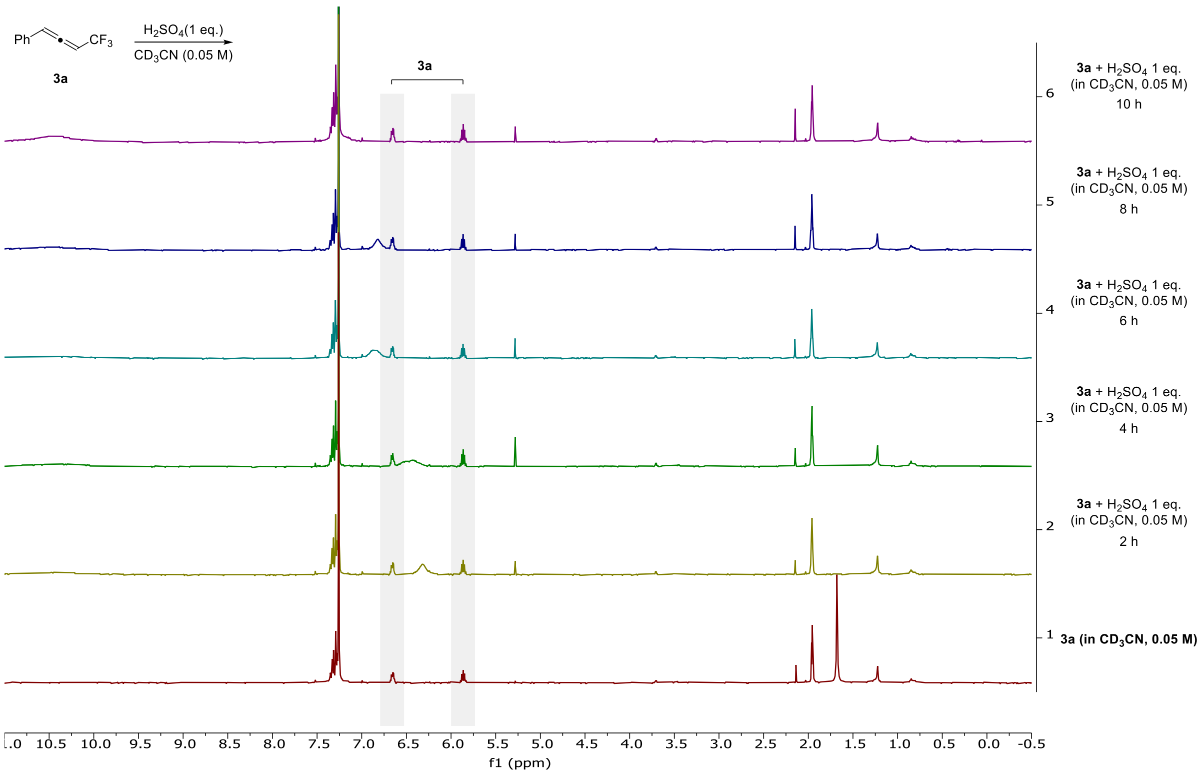

Figure S5. Stability of 3a in the presence of H_2SO_4

Details of DFT Studies

All calculations were performed by using the density functional theory (DFT) with the GAUSSIAN 16 program package[S-4]. For the initial calculation of geometry optimizations, (U)M06-2X[S-5] functional with TZVP[S-6] basis set were used. For the calculation of **1a**, **2b**, pre-association complex (**1a-2b**), pre-association complex radical intermediate (**1a-2b•**), **TS1**, **Int1**, and **Int2** stabilization, def2TZVP[S-7] basis set were used for the geometry optimization. Frequency calculation and transition state structures were performed for all stationary points to confirm the local minima, thermodynamic parameters including Gibbs free energies at 298 K. Solvent effects were considered at the polarizable continuum model (PCM) method[S-8] in acetonitrile solvent (ε = 46.8). All transition state structures were visualized by GaussView 6[S-9].

All calculated free energy differences (ΔG) are given by following equation (Eq 1.)

$$\Delta G = \Sigma G_{298}(\text{product}) - \Sigma G_{298}(\text{reactant}) \qquad (1)$$

The redox potential relative to the Ag/AgCl pseudo-reference electrode is given by following equation (Eq 2.)

$$\Delta E = -\frac{1}{F}(\Delta G - \Delta G_{SHE} - 0.197) \qquad (2)$$

ΔG_{SHE} = 4.44 eV (The absolute potential of standard hydrogen electrode)

Cartesian Coordinates and Energies of all Optimized Intermediate Geometries

1a

E(RM062X/def2TZVP): -422.906139 a. u.

G_{298}(RM062X/def2TZVP): -422.762671 a. u.

Charge = 0 / Multiplicity = 1

C	-1.31726753	-0.14608707	-0.44517344
H	-1.44947953	-1.15921807	-0.84374444
C	-2.16581853	0.75505993	-1.23253744
C	-2.83993453	1.48441393	-1.89965244
H	-3.44336953	2.13383393	-2.48731444
O	-1.76088153	-0.08866807	0.90098156
H	-1.12026453	-0.56612907	1.43868756
C	0.14681347	0.22974293	-0.57308744
C	1.04104747	-0.61543807	-1.21360444
C	0.60069347	1.42796493	-0.02829744
C	2.38339847	-0.26953107	-1.31398344
H	0.68751447	-1.54856707	-1.63726844
C	1.93896147	1.77057793	-0.12299144
H	-0.10624353	2.08288993	0.46704256
C	2.83288347	0.92223293	-0.76787944
H	3.07580447	-0.93355207	-1.81508044
H	2.28871447	2.70337293	0.30056856
H	3.87826047	1.19275793	-0.84225444

2b

E(RM062X/def2TZVP): -1048.829443 a. u.

G_{298}(RM062X/def2TZVP): -1048.700109 a. u.

Charge = 0 / Multiplicity = 1

S -0.28690008 -0.29798770 -0.00765693
O -0.98463608 -0.83425170 1.20633607
O -0.98454308 -0.83542770 -1.22137193
C 1.15971492 -1.50205270 -0.00732493
F 1.92291092 -1.37797770 -1.08454393
F 0.65941292 -2.76460370 -0.00724793
F 1.92337692 -1.37812170 1.06952407
Na -1.89123508 -2.46780370 -0.00717993

1a-2b

E(RM062X/def2TZVP): -1471.603599 a. u.

G_{298}(RM062X/def2TZVP): -1471.487399 a. u.

Charge = 0 / Multiplicity = 1

C -1.01545258 0.67328917 0.00000000
H -1.62293058 1.54159917 -0.26924200
C 0.05089842 0.50263017 -0.98895900
C 1.00623542 0.35482217 -1.69595900
H 1.88187542 0.21706517 -2.28979700
O -0.33558258 0.95464217 1.24233500
H -0.94926858 0.79353517 1.96788100

C -1.90922158 -0.53639983 0.14625500
C -3.26504758 -0.35469583 0.39216400
C -1.37689658 -1.82199783 0.11837100
C -4.08777658 -1.45117783 0.61348600
H -3.68079258 0.64684217 0.40277900
C -2.19866958 -2.91478383 0.34023900
H -0.32216058 -1.96388783 -0.08626100
C -3.55367658 -2.73065783 0.58910800
H -5.14354958 -1.30449683 0.79985900
H -1.78251858 -3.91327583 0.31538900
H -4.19355358 -3.58664283 0.75987700
S 4.42584342 -0.54150883 0.61696400
O 3.73679142 -0.31165583 1.92538400
O 3.75764242 0.30023717 -0.42783300
C 3.61237942 -2.17985783 0.17499000
F 3.91836642 -2.56968383 -1.06040100
F 2.27103642 -2.03597983 0.23224600
F 3.94770442 -3.15495383 1.01349500
Na 1.94365442 0.62470117 0.94010500

1a-2b•

E(UM062X/def2TZVP): -1471.382991 a. u.

G_{298}(UM062X/def2TZVP): -1471.265842 a. u.

Charge = 1 / Multiplicity = 2

C	-2.18029347	0.60796648	0.00000000
H	-2.68805247	-0.30189052	-0.32965700
C	-2.79921147	1.74060948	-0.69607800
C	-3.30203947	2.66041948	-1.27624400
H	-3.75468947	3.47698448	-1.78988400
O	-2.40969347	0.79891848	1.39917700
H	-1.93761047	0.10339448	1.87367600
C	-0.70069747	0.50623448	-0.30960700
C	-0.19710347	-0.61183852	-0.96005800
C	0.16200853	1.53210848	0.06945300
C	1.16276053	-0.70646152	-1.23591800
H	-0.86797047	-1.41089252	-1.25231500
C	1.51764853	1.43575548	-0.19970900
H	-0.23614147	2.40356948	0.57574200
C	2.01984353	0.31551648	-0.85569400
H	1.54964753	-1.57983852	-1.74444200
H	2.18506553	2.23447748	0.09672700
H	3.07850453	0.24247548	-1.06820400
S	-6.13554847	0.89714148	-0.39430500
O	-5.20528047	-0.12814552	0.17059900
O	-6.26355447	2.03743248	0.56426800
C	-7.76604247	0.03556948	-0.04251600
F	-8.79876947	0.80940748	-0.39162300
F	-7.89786447	-0.26083952	1.25075700
F	-7.86236747	-1.10401252	-0.73487700
Na	-4.64869947	1.20060048	2.08550900

TS1

E(UM062X/defTZVP): -1471.318010 a. u.

G_{298}(UM062X/defTZVP): -1471.253559 a. u.

Charge = 1 / Multiplicity = 2

C	-0.45454549	-0.58080807	0.00000000
C	-1.49579416	-1.37021727	0.56141325
C	-1.83828210	-2.44822056	1.16331681
H	-1.32304752	-3.32268498	1.53160736
O	-0.68728347	0.78869840	0.29690752
H	-0.04574377	1.32356170	-0.18685803
S	-3.49424693	-0.61075729	0.49432132
O	-3.50114134	0.38868099	1.43635847
O	-4.04939833	-0.51036555	-0.82303997
C	-3.86986378	-2.61243504	1.52581025
F	-3.91587145	-3.77273112	0.87530908
F	-5.14595025	-2.14700655	1.47696501
F	-3.64559275	-2.81850040	2.82236439
Na	-1.80802108	2.03567941	2.07835124
C	0.83136563	-1.11885872	0.58200147
C	1.20214520	-0.78325790	1.88122754
C	1.62700735	-1.97569048	-0.16748099
C	2.36800462	-1.30130631	2.42352459
H	0.58026963	-0.11212366	2.46145485
C	2.79177911	-2.49945040	0.37987751
H	1.33859898	-2.23178032	-1.18008016
C	3.16262538	-2.16215341	1.67387511
H	2.65725899	-1.03642770	3.43198074
H	3.41026400	-3.16515533	-0.20739900
H	4.07147341	-2.56691920	2.09948853
H	-0.45789341	-0.72895626	-1.08405367

Int 1

E(UM062X/defTZVP): -1471.449207 a. u.

G_{298}(UM062X/defTZVP): -1471.323948 a. u.

Charge = 1 / Multiplicity = 2

C	-1.52317886	0.23178808	0.00000000
C	-0.50223986	2.54091008	-0.19599700
O	-1.33798986	-0.61733292	-1.11688600
H	-1.33552486	-0.10212492	-1.93335500
C	-0.38460186	1.24495808	0.03596400
S	1.24422214	0.49181208	0.34850300
O	1.01767614	-0.92992792	0.54793500
O	1.90365914	1.25388908	1.38580100
Na	-0.04543886	-2.60695492	-0.89363700
H	-1.49280586	2.96160908	-0.33263900
C	0.60260714	3.56026908	-0.29550600
F	1.72907514	3.06481208	-0.81425200
F	0.89699614	4.08472408	0.89423200
F	0.21181514	4.56022408	-1.08784600
H	-1.42304386	-0.41388092	0.87309800
C	-2.87611986	0.90356108	0.05216600
C	-3.36069786	1.32227908	1.28865700
C	-3.62588486	1.13682608	-1.09341300
C	-4.58235186	1.97061908	1.37856800
H	-2.77778986	1.13720108	2.18366200
C	-4.85269486	1.78414008	-1.00210400
H	-3.27567286	0.81052508	-2.06434100
C	-5.33114686	2.20325408	0.23070500
H	-4.95277686	2.28879908	2.34404700
H	-5.43387386	1.95717108	-1.89810300
H	-6.28695786	2.70565408	0.29931900

Int 2

E(RM062X/defTZVP): -1,471.27403 a. u.

G_{298}(UM062X/defTZVP): -1471.224827 a. u.

Charge = 2 / Multiplicity = 1

C	-0.98484856	-0.50505050	0.00000000
C	-3.28501056	-1.71105550	0.05130500
O	-0.93710556	0.77448250	-0.83344400
H	-0.25337256	0.76121950	-1.53660600
C	-2.43810956	-0.77429450	-0.30520700
S	-2.67486756	0.62329850	-1.37357600
O	-3.32953856	1.72458350	-0.80020600
O	-2.63213556	0.34395250	-2.75125900
Na	0.84353144	3.80965750	-0.35263700
H	-2.97272956	-2.51607350	0.70761300
C	-4.71611656	-1.75988050	-0.41122500
F	-4.95524256	-2.88909850	-1.06410200
F	-4.99651056	-0.74035750	-1.23452700
F	-5.54425756	-1.68986350	0.62194000
H	-0.83291656	-0.16388850	1.02042600
C	0.02262744	-1.50538850	-0.44252600
C	1.18916744	-1.64000750	0.30651800
C	-0.17556156	-2.27571550	-1.58841800
C	2.15947744	-2.54574350	-0.09199000
H	1.33110344	-1.04212750	1.19789800
C	0.80113544	-3.17390050	-1.98387400
H	-1.08626556	-2.18296050	-2.16850500
C	1.96503044	-3.30911550	-1.23553400
H	3.06341644	-2.65712650	0.49085200
H	0.65141044	-3.77411450	-2.87064400
H	2.72209644	-4.01777850	-1.54418000

Analytic Data for Synthesized Compounds

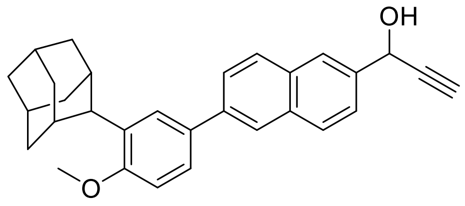

1-(6-(3-((1R,2r,3S,5r)-adamantan-2-yl)-4-methoxyphenyl)naphthalen-2-yl)prop-2-yn-1-ol, **1r**: orange solid, **^{1}H NMR (400 MHz, CDCl$_3$)** δ 8.02 – 7.99 (m, 2H), 7.92 (d, *J* = 7.9 Hz, 1H), 7.90 (d, *J* = 7.9 Hz, 1H), 7.77 (dd, *J* = 8.6, 1.7 Hz, 1H), 7.67 (dd, *J* = 8.6, 1.7 Hz, 1H), 7.62 (d, *J* = 2.4 Hz, 1H), 7.54 (dd, *J* = 8.4, 2.4 Hz, 1H), 7.00 (d, *J* = 8.4 Hz, 1H), 5.64 (d, *J* = 2.2 Hz, 1H), 3.91 (s, 3H), 2.75 (d, *J* = 2.2 Hz, 1H), 2.23 – 2.20 (m, 6H), 2.15 – 2.10 (m, 3H), 1.84 – 1.80 (m, 6H); **^{13}C NMR (101 MHz, CDCl$_3$)** δ 158.83, 139.70, 139.07, 137.12, 133.86, 133.08, 132.08, 128.96, 128.74, 126.36, 126.04, 125.79, 125.46, 124.96, 124.94, 112.28, 83.68, 75.29, 64.77, 55.35, 40.79, 37.36, 37.32, 29.30; **HRMS** m/z (EI) calc. for $C_{30}H_{30}O_2$ [M$^+$] = 422.2246, Found 422.2244.

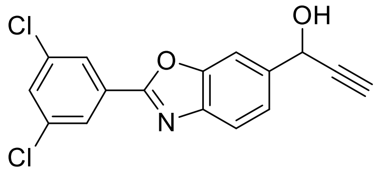

1-(2-(3,5-dichlorophenyl)benzo[*d*]oxazol-6-yl)prop-2-yn-1-ol, **1s**: yellow oil, **^{1}H NMR (400 MHz, CDCl$_3$)** δ 8.13 (d, *J* = 2.0 Hz, 2H), 7.83 (s, 1H), 7.77 (d, *J* = 8.2 Hz, 1H), 7.58 (d, *J* = 8.2 Hz, 1H), 7.52 (dd, *J* = 2.0, 2.6 Hz, 1H), 5.63 (d, *J* = 2.3 Hz, 1H), 2.73 (d, *J* = 2.3 Hz, 1H); **^{13}C NMR (101 MHz, CDCl$_3$)** δ 151.07, 142.08, 138.74, 136.07, 131.64, 129.88, 128.76, 126.10, 125.34, 120.60, 109.40, 83.37, 75.63, 64.42; **HRMS** m/z (EI) calc. for $C_{16}H_9C_{12}NO_2$ [M$^+$] = 317.0010, Found 317.0014.

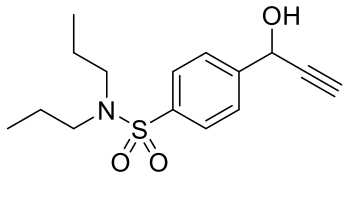

4-(1-hydroxyprop-2-yn-1-yl)-*N*,*N*-dipropylbenzenesulfonamide, **1t**: yellow oil, **^{1}H NMR (400 MHz, CDCl$_3$)** δ 7.80 (d, *J* = 8.4 Hz, 2H), 7.68 (d, *J* = 8.4 Hz, 2H), 5.53 (d, *J* = 2.2 Hz, 1H), 3.09 – 3.04 (m, 4H), 2.71 (d, *J* = 2.2 Hz, 1H), 1.58 – 1.53 (m, 4H), 0.86 (d, *J* = 7.4 Hz, 6H); **^{13}C NMR (101 MHz, CDCl$_3$)** δ 144.35, 140.27, 127.57, 127.31, 82.87, 75.81, 63.80, 50.33, 22.30, 11.39; **HRMS** m/z (EI) calc. for $C_{15}H_{21}NO_3S$ [M$^+$] = 295.1242, Found 295.1244.

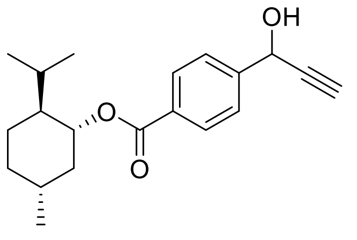

(1R,2S,5R)-2-isopropyl-5-methylcyclohexyl 4-(1-hydroxyprop-2-yn-1-yl)benzoate, **1u**: yellow oil, **^{1}H NMR (400 MHz, CDCl$_3$)** δ 8.05 (d, *J* = 8.2 Hz, 2H), 7.62 (d, *J* = 8.2 Hz, 2H), 5.52 (dd, *J* = 6.3, 2.4 Hz, 1H), 4.93 (td, *J* = 10.9, 4.4 Hz, 1H), 2.68 (d, *J* = 2.4 Hz, 1H), 2.55 (dd, *J* = 14.5, 6.3 Hz, 1H), 2.16 – 2.08 (m, 1H), 1.99 – 1.88 (m, 2H), 1.76 – 1.69 (m, 3H), 1.58 – 1.52 (m, 2H), 1.33 – 1.24 (m, 2H), 1.15 – 1.05 (m, 2H), 0.93 (d, *J* = 6.7 Hz, 6H), 0.78 (d, *J* = 6.7 Hz, 3H); **^{13}C NMR (101 MHz, CDCl$_3$)** δ 165.93, 144.79, 130.15, 126.63, 83.21,

75.45, 75.23, 64.13, 55.98, 47.47, 41.14, 35.11, 34.51, 31.64, 26.74, 25.64, 24.90, 23.86, 22.25, 20.95, 16.75; **HRMS** m/z (EI) calc. for $C_{20}H_{26}O_3$ [M^+] = 314.1882, Found 314.1888.

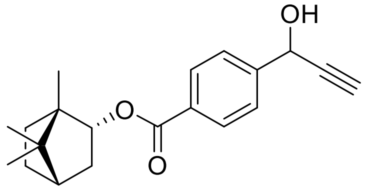

(1S,2R,4S)-1,7,7-trimethylbicyclo[2.2.1]heptan-2-yl 4-(1-hydroxyprop-2-yn-1-yl)benzoate, **1v**: brown oil, **^{1}H NMR (400 MHz, CDCl$_3$)** δ 8.08 (d, *J* = 8.4 Hz, 2H), 7.64 (d, *J* = 8.4 Hz, 2H), 5.53 (d, *J* = 2.4 Hz, 1H), 5.11 (dt, *J* = 10.0, 3.0 Hz, 1H), 2.69 (d, *J* = 2.4 Hz, 1H), 2.54 – 2.41 (m, 1H), 2.12 (ddd, *J* = 13.4, 9.4, 4.5 Hz, 1H), 1.85 – 1.76 (m, 1H), 1.74 (t, *J* = 4.5 Hz, 1H), 1.47 – 1.37 (m, 1H), 1.34 – 1.24 (m, 1H), 1.11 (dd, *J* = 13.4, 3.5 Hz, 1H), 0.97 (s, 3H), 0.91 (d, *J* = 3.5 Hz, 6H); **^{13}C NMR (101 MHz, CDCl$_3$)** δ 166.67, 144.82, 131.16, 130.10, 126.66, 83.19, 80.92, 75.50, 64.14, 49.32, 48.10, 45.19, 37.11, 28.30, 27.60, 19.93, 19.13, 13.82; **HRMS** m/z (EI) calc. for $C_{21}H_{23}F_3O_2$ [M^+] = 364.1650, Found 364.1656.

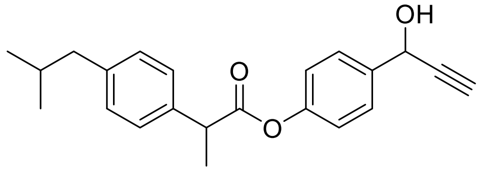

4-(1-hydroxyprop-2-yn-1-yl)phenyl 2-(4-isobutylphenyl)propanoate, **1w**: yellow oil, **^{1}H NMR (400 MHz, CDCl$_3$)** δ 7.52 (d, *J* = 8.5 Hz, 2H), 7.30 (d, *J* = 8.4 Hz, 2H), 7.14 (d, *J* = 8.5 Hz, 2H), 7.01 (d, *J* = 8.4 Hz, 2H), 5.44 (d, *J* = 2.3 Hz, 1H), 3.94 (q, *J* = 7.1 Hz, 1H), 2.65 (d, *J* = 2.3 Hz, 1H), 2.47 (d, *J* = 7.1 Hz, 2H), 2.33 (d, *J* = 6.3 Hz, 1H), 1.92 – 1.82 (m, 1H), 1.60 (d, *J* = 7.1 Hz, 4H), 0.91 (d, *J* = 6.5 Hz, 6H); **^{13}C NMR (101 MHz, CDCl$_3$)** δ 173.37, 151.12, 141.08, 137.71, 137.32, 129.73, 127.93, 127.40, 121.81, 83.45, 75.19, 64.05, 45.45, 45.25, 30.39, 22.60, 18.71; **HRMS** m/z (EI) calc. for $C_{22}H_{24}O_3$ [M^+] = 336.1725, Found 336.1731.

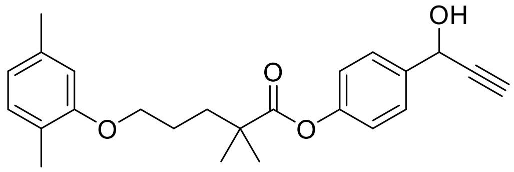

4-(1-hydroxyprop-2-yn-1-yl)phenyl 5-(2,5-dimethylphenoxy)-2,2-dimethylpentanoate, **1x**: yellow oil, **^{1}H NMR (400 MHz, CDCl$_3$)** δ 7.56 (d, *J* = 8.6 Hz, 2H), 7.05 (d, *J* = 8.6 Hz, 2H), 7.02 (s, 1H), 7.00 (s, 1H), 6.67 (d, *J* = 7.4 Hz, 1H), 6.66 – 6.59 (m, 2H), 5.47 (d, *J* = 2.3 Hz, 1H), 3.99 (t, *J* = 5.1 Hz, 2H), 2.68 (d, *J* = 2.3 Hz, 1H), 2.31 (s, 3H), 2.18 (s, 3H), 1.92 – 1.85 (m, 4H), 1.38 (s, 6H); **^{13}C NMR (101 MHz, CDCl$_3$)** δ 176.50, 157.05, 151.28, 137.66, 136.70, 130.56, 127.99, 123.81, 121.94, 120.97, 112.15, 83.48, 75.21, 67.94, 64.08, 42.67, 37.34, 25.46, 25.34, 21.61, 16.01; **HRMS** m/z (EI) calc. for $C_{24}H_{28}O_4$ [M^+] = 380.1988, Found 380.1994.

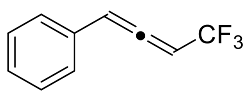

(4,4,4-trifluorobuta-1,2-dien-1-yl)benzene, **3a**: pale yellow oil; yield = 65%; **^{1}H NMR (400 MHz, CDCl$_3$)** δ 7.39 – 7.27 (m, 5H), 6.67 (qd, *J* = 7.8, 3.9 Hz, 1H), 5.92 – 5.84 (m, 1H); **^{13}C NMR (101 MHz, CDCl$_3$)** δ 207.16 (q, *J* = 5.9 Hz), 130.97 (q, *J* = 1.5 Hz), 129.17, 128.81, 127.73, 122.58 (q, *J* = 272.7 Hz), 101.53, 89.85 (q, *J* = 39.7 Hz); **^{19}F NMR (376 MHz, CDCl$_3$)** δ -60.21; **HRMS** m/z (EI) calc. fo $C_{10}H_7F_3$ [M^+] = 184.0500, found 184.0497; ***R_f*** = 0.8 (pentane).

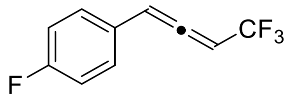

1-fluoro-4-(4,4,4-trifluorobuta-1,2-dien-1-yl)benzene **3b**: yellow oil; yield = 31%; **^{1}H NMR (400 MHz, CDCl$_3$)** δ 7.29 (dd, *J* = 8.8, 5.3 Hz, 2H), 7.05 (dd, *J* = 8.8, 8.7 Hz, 2H), 6.65 (qd, *J* = 7.3, 3.8 Hz, 1H), 5.93 – 5.85 (m, 1H); **^{13}C NMR (101 MHz, CDCl$_3$)** δ 206.96 (qd, *J* = 5.6, 2.8 Hz), 163.04 (d, *J* = 249.6 Hz), 129.37 (d, *J* = 8.4 Hz), 126.97 (q, *J* = 1.4 Hz), 122.50 (q, *J* = 272.1 Hz), 116.28 (d, *J* = 22.3 Hz), 116.28, 90.07 (q, *J* = 39.3 Hz); **^{19}F NMR (376 MHz, CDCl$_3$)** δ -60.29 (3F), -112.50 (1F); **HRMS** m/z (EI) calc. for $C_{10}H_6F_4$ [M^+] = 202.0406, Found 202.0403; ***R_f*** = 0.8 (pentane).

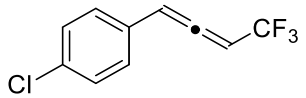

1-chloro-4-(4,4,4-trifluorobuta-1,2-dien-1-yl)benzene **3c**: pale yellow oil; yield = 53%; **^{1}H NMR (400 MHz, CDCl$_3$)** δ 7.33 (d, *J* = 8.5 Hz, 2H), 7.24 (d, *J* = 8.5 Hz, 2H), 6.64 (qd, *J* = 7.6, 3.9 Hz, 1H), 5.93 – 5.85 (m, 1H); **^{13}C NMR (101 MHz, CDCl$_3$)** δ 207.17 (q, *J* = 5.9 Hz), 134.66, 133.29, 129.42, 128.90, 122.43 (q, *J* = 272.1 Hz), 100.66, 90.28 (q, *J* = 39.4 Hz); **^{19}F NMR (376 MHz, CDCl$_3$)** δ -60.29 (3F); **HRMS** m/z (EI) calc. for $C_{10}H_6ClF_3$ [M^+] = 218.0110, Found 218.0107; ***R_f*** = 0.8 (pentane).

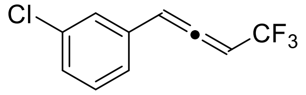

1-chloro-3-(4,4,4-trifluorobuta-1,2-dien-1-yl)benzene **3d**: yellow oil; yield = 58%; **^{1}H NMR (400 MHz, CDCl$_3$)** δ 7.32 – 7.26 (m, 3H), 7.21 – 7.17 (m, 1H), 6.62 (qd, *J* = 7.6, 3.8 Hz, 1H), 5.95 – 5.88 (m, 1H); **^{13}C NMR (101 MHz, CDCl$_3$)** δ 207.23 (q, *J* = 6.2 Hz), 135.16, 132.91, 130.37, 128.88, 127.61, 125.85, 122.41 (q, *J* = 273.2 Hz), 100.61, 90.41 (q, *J* = 39.0 Hz); **^{19}F NMR (376 MHz, CDCl$_3$)** δ -60.17; **HRMS** m/z (EI) calc. for $C_{10}H_6ClF_3$ [M^+] = 218.0110, Found 218.0107; ***R_f*** = 0.8 (pentane).

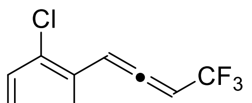

1-chloro-2-(4,4,4-trifluorobuta-1,2-dien-1-yl)benzene **3e**: yellow oil; yield = 54%; **^{1}H NMR (400 MHz, CDCl$_3$)** δ 7.43 – 7.37 (m, 2H), 7.28 – 7.20 (m, 2H), 7.14 (qd, *J* = 7.6, 3.8 Hz, 1H), 5.95 – 5.88 (m, 1H); **^{13}C NMR (101 MHz, CDCl$_3$)** δ 207.85 (q, *J* = 5.5 Hz), 138.56, 133.08, 130.20, 129.88, 129.10, 127.41, 122.51 (q, *J* = 272.3 Hz), 98.06, 90.09 (q, *J* = 39.8 Hz); **^{19}F NMR (376 MHz, CDCl$_3$)** δ -60.09; **HRMS** m/z (EI) calc. for $C_{10}H_6ClF_3$ [M^+] = 218.0110, Found 218.0108; ***R_f*** = 0.8 (pentane).

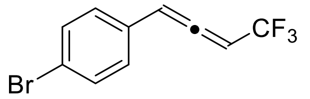

1-bromo-4-(4,4,4-trifluorobuta-1,2-dien-1-yl)benzene **3f**: yellow oil; yield = 39%; **^{1}H NMR (400 MHz, CDCl$_3$)** δ 7.48 (d, *J* = 8.5 Hz, 2H), 7.17 (d, *J* = 8.5 Hz, 2H), 6.62 (qd, *J* = 7.6, 3.8 Hz, 1H), 5.95 – 5.88 (m, 1H); **^{13}C NMR (101 MHz, CDCl$_3$)** δ 207.16 (q, *J* = 5.7 Hz), 132.37, 129.97, 129.17, 122.79, 122.37 (q, *J* = 272.2 Hz) 100.75 90.35 (q, *J* = 39.6 Hz); **^{19}F NMR (376 MHz, CDCl$_3$)** δ -60.20; **HRMS** m/z (EI) calc. for $C_{10}H_6BrF_3$ [M$^+$] = 261.9605, Found 261.9602; ***R$_f$*** = 0.8 (pentane).

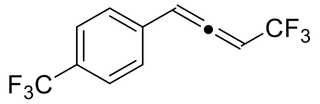

1-(4,4,4-trifluorobuta-1,2-dien-1-yl)-4-(trifluoromethyl)benzene **3g**: pale yellow oil; yield = 41%; **^{1}H NMR (400 MHz, CDCl$_3$)** δ 7.61 (d, *J* = 8.4 Hz, 2H), 7.42 (d, *J* = 8.4 Hz, 2H), 6.71 (qd, *J* = 7.4, 3.8 Hz, 1H), 5.90 – 5.82 (m, 1H); **^{13}C NMR (101 MHz, CDCl$_3$)** δ 207.65 (q, *J* = 6.2 Hz), 134.84, 130.75 (q, *J* = 32.4 Hz), 127.92, 126.15 (q, *J* = 4.0 Hz), 124.12 (q, *J* = 273.5 Hz), 122.37 (q, *J* = 271.8 Hz), 100.64, 90.57 (q, *J* = 39.7 Hz); **^{19}F NMR (376 MHz, CDCl$_3$)** δ -60.13 (3F), -62.76 (3F); **HRMS** m/z (EI) calc. for $C_{11}H_6F_6$ [M$^+$] = 252.0374, Found 252.0370; ***R$_f$*** = 0.8 (pentane).

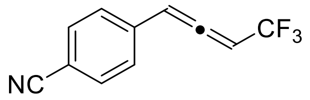

4-(4,4,4-trifluorobuta-1,2-dien-1-yl)benzonitrile **3h**: pale yellow solid; yield = 50%; **^{1}H NMR (400 MHz, CDCl$_3$)** δ 7.65 (d, *J* = 8.5 Hz, 2H), 7.41 (d, *J* = 8.5 Hz, 2H), 6.70 (qd, *J* = 7.3, 3.6 Hz, 1H), 6.02 – 5.95 (m, 1H); **^{13}C NMR (101 MHz, CDCl$_3$)** δ 207.71 (q, *J* = 5.9 Hz), 135.79, 132.74, 128.00, 122.03 (q, *J* = 272.4 Hz), 118.48, 112.10, 100.42 90.75 (q, *J* = 39.5 Hz); **^{19}F NMR (376 MHz, CDCl$_3$)** δ -60.04; **HRMS** m/z (EI) calc. for $C_{11}H_6F_3N$ [M$^+$] = 209.0452, Found 209.0450; ***R$_f$*** = 0.3 (ethyl acetate/pentane = 1/20).

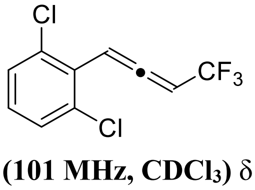

1,3-dichloro-2-(4,4,4-trifluorobuta-1,2-dien-1-yl)benzene **3i**: pale yellow oil; yield = 53%; **^{1}H NMR (400 MHz, CDCl$_3$)** δ 7.34 (d, *J* = 8.0 Hz, 2H), 7.17 (t, *J* = 8.0 Hz, 1H), 7.07 (qd, *J* = 8.5, 4.3 Hz, 1H), 5.82 – 5.75 (m, 1H); **^{13}C NMR (101 MHz, CDCl$_3$)** δ 208.74 (q, *J* = 5.9 Hz), 135.34, 129.53, 129.02, 127.84, 122.61 (q, *J* = 272.2 Hz), 94.94, 88.60 (q, *J* = 39.5 Hz); **^{19}F NMR (376 MHz, CDCl$_3$)** δ -60.31; **HRMS** m/z (EI) calc. for $C_{10}H_5Cl_2F_3$ [M$^+$] = 251.9720, Found 251.9716 ***R$_f$*** = 0.8 (pentane).

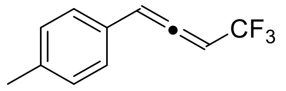

1-methyl-4-(4,4,4-trifluorobuta-1,2-dien-1-yl)benzene **3j**: pale yellow oil; yield = 42%; **^{1}H NMR (400 MHz, CDCl$_3$)** δ 7.21 (d, *J* = 8.3 Hz, 2H), 7.17 (d, *J* = 8.3 Hz, 2H), 6.65 (qd, *J* = 7.8, 3.9 Hz, 1H), 5.90 – 5.82 (m, 1H), 2.36 (s, 3H); **^{13}C NMR (101 MHz, CDCl$_3$)** δ 207.09 (q , *J* = 5.8 Hz), 138.89, 129.89, 127.95, 127.64, 122.64 (q, *J* = 272.0 Hz),

101.37, 89.71 (q, J = 39.2 Hz), 21.50; **^{19}F NMR (376 MHz, CDCl$_3$)** δ -60.31; **HRMS** m/z (EI) calc. for $C_{11}H_9F_3$ [M^+] = 198.0656, Found 198.0658; ***R$_f$*** = 0.8 (pentane).

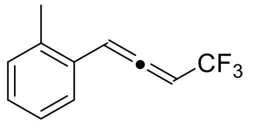

1-methyl-2-(4,4,4-trifluorobuta-1,2-dien-1-yl)benzene **3k**: pale yellow oil; yield = 46%; **^{1}H NMR (400 MHz, CDCl$_3$)** δ 7.41 – 7.36 (m, 1H), 7.29 – 7.21 (m, 3H), 6.92 (qd, J = 8.0, 4.0 Hz, 1H), 5.90 – 5.82 (m, 1H), 2.44 (s, 3H); **^{13}C NMR (101 MHz, CDCl$_3$)** δ 207.71 (q, J = 6.0 Hz), 136.25, 131.00, 129.45, 128.74, 128.36, 126.68, 122.75 (q, J = 272.9 Hz), 99.05, 88.94 (q, J = 39.2 Hz), 20.17; **^{19}F NMR (376 MHz, CDCl$_3$)** δ -60.23; **HRMS** m/z (EI) calc. for $C_{11}H_9F_3$ [M^+] = 198.0656, Found 198.0658; ***R$_f$*** = 0.8 (pentane).

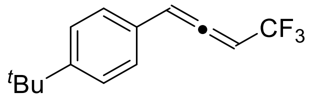

1-(tert-butyl)-4-(4,4,4-trifluorobuta-1,2-dien-1-yl)benzene **3l**: pale yellow oil; yield = 35%; **^{1}H NMR (400 MHz, CDCl$_3$)** δ 7.39 (d, J = 8.5 Hz, 2H), 7.26 (d, J = 8.5 Hz, 2H), 6.66 (qd, J = 7.8, 3.9 Hz, 1H), 5.82 – 5.75 (m, 1H), 1.32 (s, 9H); **^{13}C NMR (101 MHz, CDCl$_3$)** δ 207.21 (q, J = 6.0 Hz), 152.14, 127.98, 127.49, 126.15, 122.62 (q, J = 273.0 Hz), 101.22, 89.67 (q, J = 39.6 Hz), 34.92, 31.43; **^{19}F NMR (376 MHz, CDCl$_3$)** δ -60.25; **HRMS** m/z (EI) calc. for $C_{14}H_{15}F_3$ [M^+] = 240.1126, Found 240.1129; ***R$_f$*** = 0.8 (pentane).

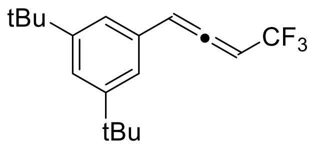

1-(tert-butyl)-4-(4,4,4-trifluorobuta-1,2-dien-1-yl)benzene **3m**: yellow oil; yield = 49%; **^{1}H NMR (400 MHz, CDCl$_3$)** δ 7.41 (t, J = 1.8 Hz, 1H), 7.18 (d, J = 1.8 Hz, 2H), 6.70 (qd, J = 8.0, 4.0 Hz, 1H), 5.82 – 5.75 (m, 1H), 1.35 (s, 18H); **^{13}C NMR (101 MHz, CDCl$_3$)** δ 207.26 (q, J = 5.7 Hz), 151.77, 123.22, 122.72 (q, J = 273.0 Hz), 122.04, 102.24, 89.65 (q, J = 38.8 Hz), 35.09, 31.55; **^{19}F NMR (376 MHz, CDCl$_3$)** δ -60.16; **HRMS** m/z (EI) calc. for $C_{18}H_{23}F_3$ [M^+] = 296.1752, Found 296.1756; ***R$_f$*** = 0.6 (pentane).

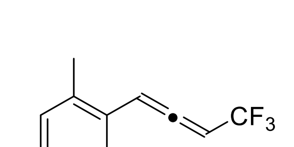

1,3,5-trimethyl-2-(4,4,4-trifluorobuta-1,2-dien-1-yl)benzene **3n**: pale yellow oil; yield = 46%; **^{1}H NMR (400 MHz, CDCl$_3$)** δ 6.89 (s, 2H), 6.85 – 6.78 (m, 1H), 5.67 – 5.60 (m, 1H), 2.33 (s, 6H), 2.28 (s, 3H); **^{13}C NMR (101 MHz, CDCl$_3$)** δ 207.28 (q, J = 5.9 Hz), 138.07, 137.26, 132.55, 129.48, 122.95 (q, J = 271.4 Hz), 96.71, 86.64 (q, J = 39.3 Hz), 21.37, 21.17; **^{19}F NMR (376 MHz, CDCl$_3$)** δ -60.30; **HRMS** m/z (EI) calc. for $C_{13}H_{13}F_3$ [M^+] = 226.0969, Found 226.0972; ***R$_f$*** = 0.6 (pentane).

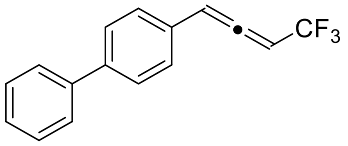

4-(4,4,4-trifluorobuta-1,2-dien-1-yl)-1,1'-biphenyl **3o**: white solid; yield = 46%; **^{1}H NMR (400 MHz, CDCl$_3$)** δ 7.61 – 7.58 (m, 4H), 7.45 (dd, *J* = 8.1, 7.8 Hz, 2H), 7.40 – 7.34 (m, 3H), 6.72 (qd, *J* = 7.6, 3.8 Hz, 1H), 5.95 – 5.88 (m, 1H); **^{13}C NMR (101 MHz, CDCl$_3$)** δ 207.38 (q, *J* = 5.9 Hz), 141.70, 140.58, 129.90, 129.89, 129.09, 128.16, 127.88, 127.84, 127.22, 122.57 (q, *J* = 272.5 Hz), 101.23 89.67 (q, *J* = 40.0 Hz); **^{19}F NMR (376 MHz, CDCl$_3$)** δ -60.17; **HRMS** m/z (EI) calc. for $C_{16}H_{11}F_3$ [M$^+$] = 260.0813, Found 260.0814; ***R$_f$*** = 0.2 (ethyl acetate/pentane = 1:50).

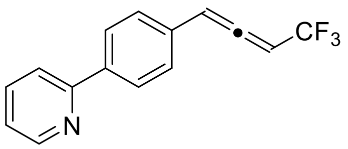

2-(4-(4,4,4-trifluorobuta-1,2-dien-1-yl)phenyl)pyridine **3p**: white solid; yield = 58%; **^{1}H NMR (400 MHz, CDCl$_3$)** δ 8.70 (dt, *J* =, 4.7, 1.3 Hz, 1H), 8.00 (d, *J* = 8.4 Hz, 2H), 7.81 – 7.70 (m, 2H), 7.42 (d, *J* = 8.4 Hz, 2H), 7.28 – 7.21 (m, 2H), 6.73 (qd, *J* = 7.6, 3.8 Hz, 1H), 5.97 – 5.89 (m, 1H); **^{13}C NMR (101 MHz, CDCl$_3$)** δ 207.53 (q, *J* = 5.7 Hz), 156.76, 149.93, 139.69, 137.13, 131.63, 131.62, 128.14, 127.66, 122.62, 122.55 (q, *J* = 272.5 Hz), 120.73, 101.24, 90.05 (q, *J* = 39.5 Hz); **^{19}F NMR (376 MHz, CDCl$_3$)** δ -60.12; **HRMS** m/z (EI) calc. for $C_{15}H_{10}F_3N$ [M$^+$] = 261.0765, Found 261.0763; ***R$_f$*** = 0.3 (ethyl acetate/pentane = 1:30).

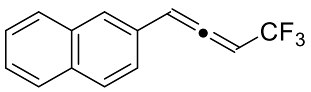

2-(4,4,4-trifluorobuta-1,2-dien-1-yl)naphthalene[S-11] **3q**: yellow solid; yield = 33%; **^{1}H NMR (400 MHz, CDCl$_3$)** δ 7.84 – 7.78 (m, 3H), 7.72 (s, 1H), 7.52 – 7.47 (m, 2H), 7.46 – 7.41 (m, 1H), 6.85 (qd, *J* = 7.6, 3.6 Hz, 1H), 5.99 – 5.92 (m, 1H); **^{13}C NMR (101 MHz, CDCl$_3$)** δ 207.06 (q, *J* = 5.9 Hz), 133.69, 133.50, 129.00, 128.41, 128.18, 128.03, 127.40, 126.85, 126.78, 124.69, 122.60 (q, *J* = 272.3 Hz), 101.88, 90.12 (q, *J* = 39.1 Hz); **^{19}F NMR (376 MHz, CDCl$_3$)** δ -60.17; **HRMS** m/z (EI) calc. for $C_{14}H_9F_3$ [M$^+$] = 234.0656, Found 234.0653; ***R$_f$*** = 0.3 (ethyl acetate/pentane = 1:15).

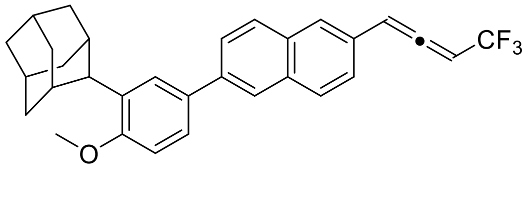

(1R,2r,3S,5r)-2-(2-methoxy-5-(6-(4,4,4-trifluorobuta-1,2-dien-1-yl)naphthalen-2-yl)phenyl)adamantane **3r**: brown oil; yield = 21%; **^{1}H NMR (400 MHz, CDCl$_3$)** δ 7.96 (s, 1H), 7.85 (dd, *J* = 8.4, 2.5 Hz, 2H), 7.77 – 7.71 (m, 2H), 7.59 (d, *J* = 2.5 Hz, 1H), 7.53 (dd, *J* = 8.4, 2.4 Hz, 1H), 7.44 (dd, *J* = 8.5, 2.5 Hz, 1H), 6.99 (d, *J* = 8.5 Hz, 1H), 6.85 (qd, *J* = 7.6, 3.8 Hz, 1H), 6.00 – 5.92 (m, 1H), 3.90 (s, 3H), 2.20 – 2.17 (m, 6H), 2.13 – 2.09 (m, 3H), 1.82 – 1.77 (m, 6H); **^{13}C NMR (101 MHz, CDCl$_3$)** δ 207.69 (q, *J* = 6.1 Hz), 158.93, 139.91, 139.16, 133.87, 133.01, 132.44, 129.12, 128.52, 127.93, 127.16, 126.63, 126.07, 125.80, 125.11, 125.00, 122.61 (q, *J* = 272.1 Hz), 112.31, 101.96, 90.08 (q, *J*

= 39.7 Hz), 55.39, 40.83, 37.35, 37.32, 29.92, 29.34; **19F NMR (376 MHz, CDCl3)** δ -59.75; **HRMS** m/z (EI) calc. for $C_{31}H_{29}F_3O$ [M^+] = 474.2171, Found 474.2168; ***Rf*** = 0.2 (ethyl acetate/pentane = 1:8).

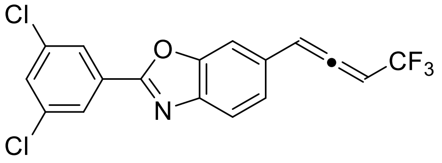

2-(3,5-dichlorophenyl)-6-(4,4,4-trifluorobuta-1,2-dien-1-yl)benzo[d]oxazole **3s**: yellow oil; yield = 47%; **1H NMR (400 MHz, CDCl3)** δ 8.13 (d, *J* = 1.9 Hz, 2H), 7.74 (d, *J* = 8.3 Hz, 1H), 7.54 (d, *J* = 2.2 Hz, 1H), 7.52 (dd, *J* = 2.2, 1.9 Hz, 1H), 7.35 (d, *J* = 8.3 Hz, 1H), 6.80 (qd, *J* = 7.6, 3.8 Hz, 1H), 6.01 – 5.94 (m, 1H); **13C NMR (101 MHz, CDCl3)** δ 207.42 (q, *J* = 6.8 Hz), 161.48, 151.51, 142.42, 136.11, 131.71, 129.80, 129.31, 126.11, 125.09, 122.45 (q, *J* = 272.7 Hz), 120.92, 109.74, 101.53, 90.49 (q, *J* = 39.9 Hz); **19F NMR (376 MHz, CDCl3)** δ -60.16; **HRMS** m/z (EI) calc. for $C_{17}H_8Cl_2F_3NO$ [M^+] = 368.9935, Found 368.9930; ***Rf*** = 0.3 (ethyl acetate/pentane = 1:10).

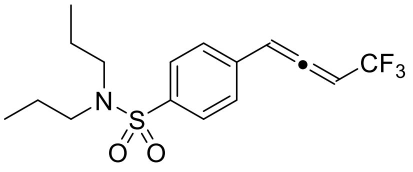

N,*N*-dipropyl-4-(4,4,4-trifluorobuta-1,2-dien-1-yl)benzenesulfonamide **3t**: white solid; yield = 47%; **1H NMR (400 MHz, CDCl3)** δ 7.79 (d, *J* = 8.5 Hz, 2H), 7.42 (d, *J* = 8.5 Hz, 2H), 6.71 (qd, *J* = 7.6, 3.8 Hz, 1H), 6.01 – 5.94 (m, 1H), 3.10 – 3.05 (m, 4H), 1.56 (qt, *J* = 8.9, 7.4 Hz 4H), 0.87 (t, *J* = 7.4 Hz, 6H); **13C NMR (101 MHz, CDCl3)** δ 207.82 (q, *J* = 6.0 Hz), 140.29, 129.07, 128.09, 127.93, 122.32 (q, *J* = 272.9 Hz), 100.57, 90.67 (q, *J* = 39.8 Hz), 50.25, 22.24, 11.39; **19F NMR (376 MHz, CDCl3)** δ -60.05; **HRMS** m/z (EI) calc. for $C_{17}H_8Cl_2F_3NO$ [M^+] = 368.9935, Found 368.9931; ***Rf*** = 0.4 (ethyl acetate/pentane = 1:10).

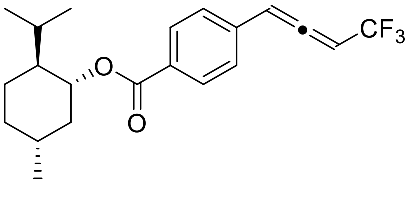

(1*R*,2*S*,5*R*)-2-isopropyl-5-methylcyclohexyl 4-(4,4,4-trifluorobuta-1,2-dien-1-yl)benzoate **3u**: pale yellow oil; yield = 47%; **1H NMR (400 MHz, CDCl3)** δ 8.02 (d, *J* = 8.4 Hz, 2H), 7.37 (d, *J* = 8.4 Hz, 2H), 6.75 – 6.67 (m, 1H), 5.98 – 5.90 (m, 1H), 4.93 (dt, *J* = 10.8, 4.4 Hz, 1H), 2.16 – 2.09 (m, 1H), 1.98 – 1.90 (m, 1H), 1.77 – 1.69 (m, 3H), 1.58 – 1.50 (m, 3H), 1.19 – 1.03 (m, 2H), 0.93 (dd, *J* = 7.4, 6.9 Hz, 6H), 0.79 (d, *J* = 6.9 Hz, 3H); **13C NMR (101 MHz, CDCl3)** δ 207.81 (q, *J* = 5.6 Hz), 165.75, 135.50, 131.06, 130.41, 127.56, 122.42 (q, *J* = 271.7 Hz), 101.04, 90.34 (q, *J* = 39.5 Hz), 75.30, 47.49, 41.17, 34.52, 31.67, 26.79, 23.91, 22.26, 20.96, 16.76; **19F NMR (376 MHz, CDCl3)** δ -60.10; **HRMS** m/z (EI) calc. for $C_{17}H_8Cl_2F_3NO$ [M^+] = 368.9935, Found 368.9941; ***Rf*** = 0.4 (ethyl acetate/pentane = 1:7).

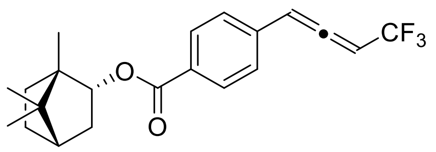

(1*S*,2*R*,4*S*)-1,7,7-trimethylbicyclo[2.2.1]heptan-2-yl 4-(4,4,4-trifluorobuta-1,2-dien-1-yl)benzoate **3v**: pale yellow oil; yield = 41%; **^{1}H NMR (400 MHz, CDCl$_3$)** δ 8.04 (d, *J* = 8.3 Hz, 2H), 7.38 (d, *J* = 8.3 Hz, 2H), 6.72 (qd, *J* = 7.2, 3.7 Hz, 1H), 5.99 – 5.90 (m, 1H), 5.18 – 5.07 (m, 1H), 2.50 – 2.43 (m, 1H), 2.11 (dt, *J* = 9.3, 4.8 Hz, 1H), 1.83 – 1.74 (m, 2H), 1.42 (t, *J* = 9.4 Hz, 1H), 1.31 (s, 1H), 1.14 – 1.10 (m, 1H), 0.97 (s, 3H), 0.92 (s, 3H), 0.91 (s, 3H); **^{13}C NMR (101 MHz, CDCl$_3$)** δ 207.83 (q, *J* = 5.6 Hz), 166.47, 135.56, 131.08, 130.36, 127.60, 122.42 (q, *J* = 271.7 Hz), 101.04, 90.36 (q, *J* = 39.6 Hz), 80.97, 49.34, 48.12, 45.21, 37.14, 28.31, 27.61, 19.94, 19.13, 13.83; **^{19}F NMR (376 MHz, CDCl$_3$)** δ -60.10; **HRMS** m/z (EI) calc. for $C_{17}H_8Cl_2F_3NO$ [M$^+$] = 368.9935, Found 368.9938; ***R$_f$*** = 0.4 (ethyl acetate/pentane = 1:8).

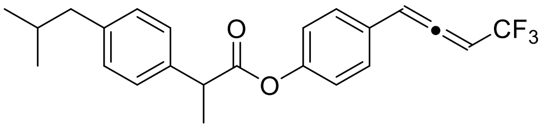

4-(4,4,4-trifluorobuta-1,2-dien-1-yl)phenyl 2-(4-isobutylphenyl)propanoate **3w**: pale yellow oil; yield = 72%; **^{1}H NMR (400 MHz, CDCl$_3$)** δ 7.29 (d, *J* = 7.8 Hz, 2H), 7.27 (d, *J* = 7.8 Hz, 2H), 7.14 (d, *J* = 8.4 Hz, 2H), 6.99 (d, *J* = 8.4 Hz, 2H), 6.64 (qd, *J* = 7.6, 3.9 Hz, 1H), 5.91 – 5.83 (m, 1H), 3.93 (q, J = 7.2 Hz, 1H), 2.47 (d, *J* = 7.2 Hz, 2H), 1.89 – 1.83 (m, 1H), 1.60 (d, *J* = 7.0 Hz, 3H), 0.91 (d, *J* = 7.0 Hz, 6H); **^{13}C NMR (101 MHz, CDCl$_3$)** δ 207.15 (q, *J* = 5.9 Hz), 173.30, 151.22, 141.13, 137.28, 129.76, 128.65, 127.41, 122.28, 122.42 (q, *J* = 272.5 Hz), 100.78, 90.03 (q, *J* = 39.3 Hz), 45.47, 45.26, 30.41, 22.61, 18.71; **^{19}F NMR (376 MHz, CDCl$_3$)** δ -60.10; **HRMS** m/z (EI) calc. for $C_{17}H_8Cl_2F_3NO$ [M$^+$] = 368.9935, Found 368.9938; ***R$_f$*** = 0.3 (ethyl acetate/pentane = 1:10).

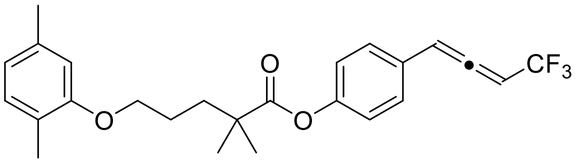

4-(4,4,4-trifluorobuta-1,2-dien-1-yl)phenyl 2-(4-isobutylphenyl)propanoate **3x**: pale yellow oil; yield = 61%; **^{1}H NMR (400 MHz, CDCl$_3$)** δ 7.30 (d, *J* = 8.6 Hz, 2H), 7.05 – 6.97 (m, 3H), 6.70 – 6.60 (m, 3H), 5.93 – 5.85 (m, 1H), 3.98 (t, *J* = 5.1 Hz, 2H), 2.30 (s, 3H), 2.17 (s, 3H), 1.92 – 1.84 (m, 4H), 1.37 (s, 6H); **^{13}C NMR (101 MHz, CDCl$_3$)** δ 207.16 (q, *J* = 5.7 Hz), 176.43, 157.05, 151.38, 136.72, 130.58, 128.71, 128.44, 123.83, 122.50 (q, *J* = 272.1 Hz), 122.41, 120.99, 112.16, 100.81, 90.05 (q, *J* = 39.8 Hz), 67.93, 42.71, 37.35, 25.48, 25.33, 21.61, 16.01; **^{19}F NMR (376 MHz, CDCl$_3$)** δ -60.10; **HRMS** m/z (EI) calc. for $C_{17}H_8Cl_2F_3NO$ [M$^+$] = 368.9935, Found 368.9941; ***R$_f$*** = 0.3 (ethyl acetate/pentane = 1:10).

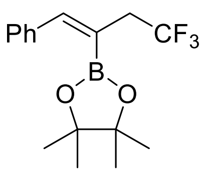

(*E*)-4,4,5,5-tetramethyl-2-(4,4,4-trifluoro-1-phenylbut-1-en-2-yl)-1,3,2-dioxaborolane **7**: colorless oil; yield = 89%; **^{1}H NMR (400 MHz, CDCl$_3$)** δ 7.53 (s, 1H), 7.42 – 7.27 (m, 5H), 3.16 (q, *J* = 10.8 Hz, 2H), 1.31 (s, 12H); **^{13}C NMR (101 MHz, CDCl$_3$)** δ 148.49, 136.74, 128.77, 128.63, 128.15, 126.61 (q, *J* = 279.0 Hz), 84.25, 33.86 (q, *J* = 28.8 Hz), 24.88; **^{19}F NMR (376 MHz, CDCl$_3$)** δ -63.79 (t, *J* = 10.8 Hz); **LRMS** m/z (EI) calc. for $C_{16}H_{20}BF_3O_2$ [M^+] = 312.1, Found 312.2; ***R$_f$*** = 0.3 (hexanes).

References

S-1 L. Eccleshare, L. Lozada-Rodríguez, P. Cooper, L. Burroughs, J. Ritchie, W. Lewis, S. Woodward, *Chem. Eur. J.* **2016**, *22*, 12542−12657.

S-2 J. E. Gillespie, N. Y. S. Lam, R. J. Phipps, *Chem. Sci.* **2023**, *14*, 10103−10111.

S-3 M.-S. Liu, W. Shu, *ACS Catal.* **2020**, *10*, 12960-12966.

S-4 M. J. Frisch, G. W. Trucks, H. B. Schlegel, G. E. Scuseria, M. A. Robb, J. R. Cheeseman, G. Scalmani, V. Barone, B. Mennucci, G. A. Petersson, H. Nakatsuji, M. Caricato, X. Li, H. P. Hratchian, A. F. Izmaylov, J. Bloino, G. Zheng, J. L. Sonnenberg, M. Hada, M. Ehara, K. Toyota, R. Fukuda, J. Hasegawa, M. Ishida, T. Nakajima, Y. Honda, O. Kitao, H. Nakai, T. Vreven, J. A. Montgomery Jr., J. E. Peralta, F. Ogliaro, M. Bearpark, J. J. Heyd, E. Brothers, K. N. Kudin, V. N. Staroverov, R. Kobayashi, J. Normand, K. Raghavachari, A. Rendell, J. C. Burant, S. S. Iyengar, J. Tomasi, M. Cossi, N. Rega, J. M. Millam, M. Klene, J. E. Knox, J. B. Cross, V. Bakken, C. Adamo, J. Jaramillo, R. Gomperts, R. E. Stratmann, O. Yazyev, A. J. Austin, R. Cammi, C. Pomelli, J. W. Ochterski, R. L. Martin, K. Morokuma, V. G. Zakrzewski, G. A. Voth, P. Salvador, J. J. Dannenberg, S. Dapprich, A. D. Daniels, O. Farkas, J. B. Foresman, J. V. Ortiz, J. Cioslowski, D. J. Fox, Gaussian 09, Revision D01; Gaussian, Inc., Wallingford, CT, **2016**

S-5 The M06 suite of density functionals for main group thermochemistry, thermochemical kinetics, noncovalent interactions, excited states, and transition elements: two new functionals and systematic testing of four M06-class functionals and 12 other functionals: Y. Zhao, D. G. Truhlar, *Theor. Chem. Acc.* **2008**, *120*, 215−241.

S-6 Fully optimized contracted Gaussian-basis sets of triple zeta valence quality for atoms Li to Kr: A. Schaefer, C. Huber, R. Ahlrichs, *J. Chem. Phys.*, **1994**, *100*, 5829−5835.

S-7 Balanced basis sets of split valence, triple zeta valence and quadruple zeta valence quality for H to Rn: Design and assessment of accuracy: F. Weigend, R. Ahlrichs, *Phys. Chem. Chem. Phys.* **2005**, *7*, 3297−3305.

S-8 Quantum Mechanical Continuum Solvation Models: J. Tomasi, B. Mennucci, R. Cammi, *Chem. Rev.* **2005**, *105*, 2999−3094.

S-9 GaussView, Version 6, Dennington, Roy; Keith, Todd; Millam, John. Semichem Inc., Shawnee Mission, KS, **2016**.

NMR Spectra (^{1}H NMR, ^{13}C NMR, and ^{19}F NMR)

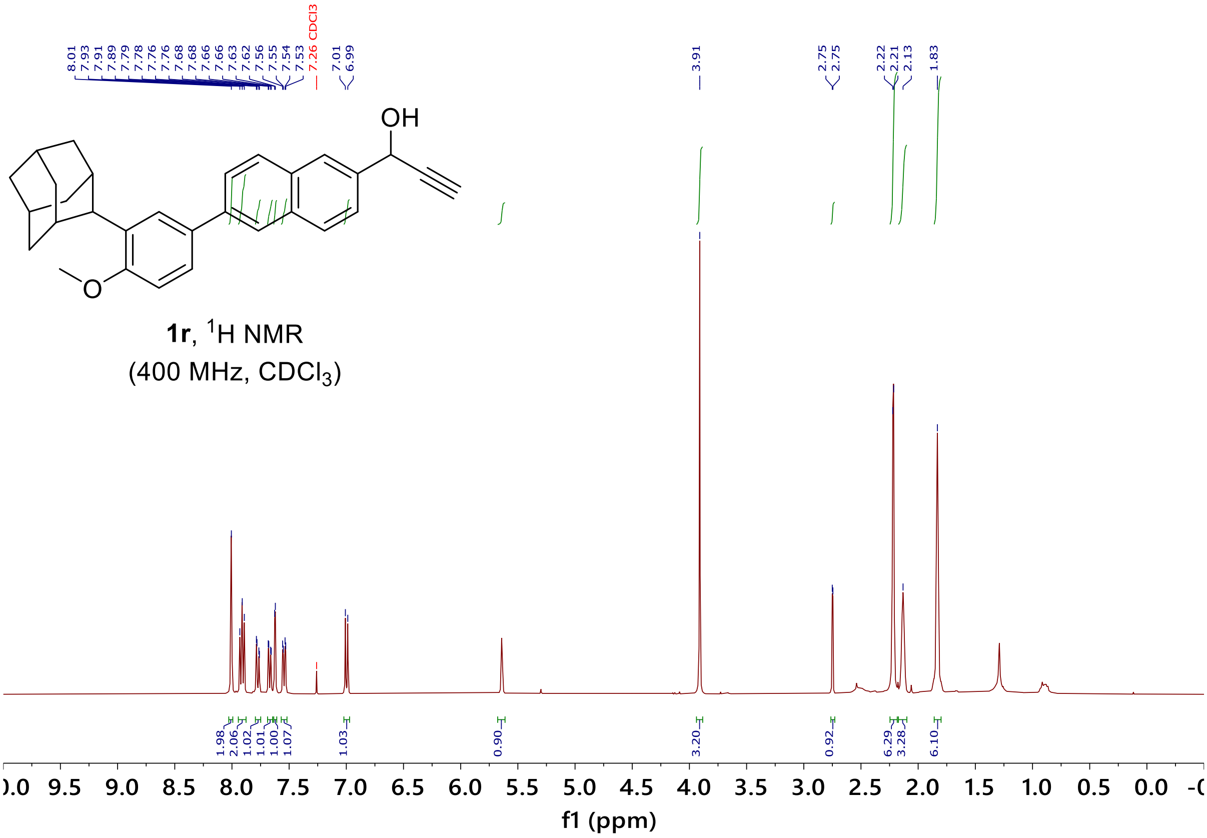

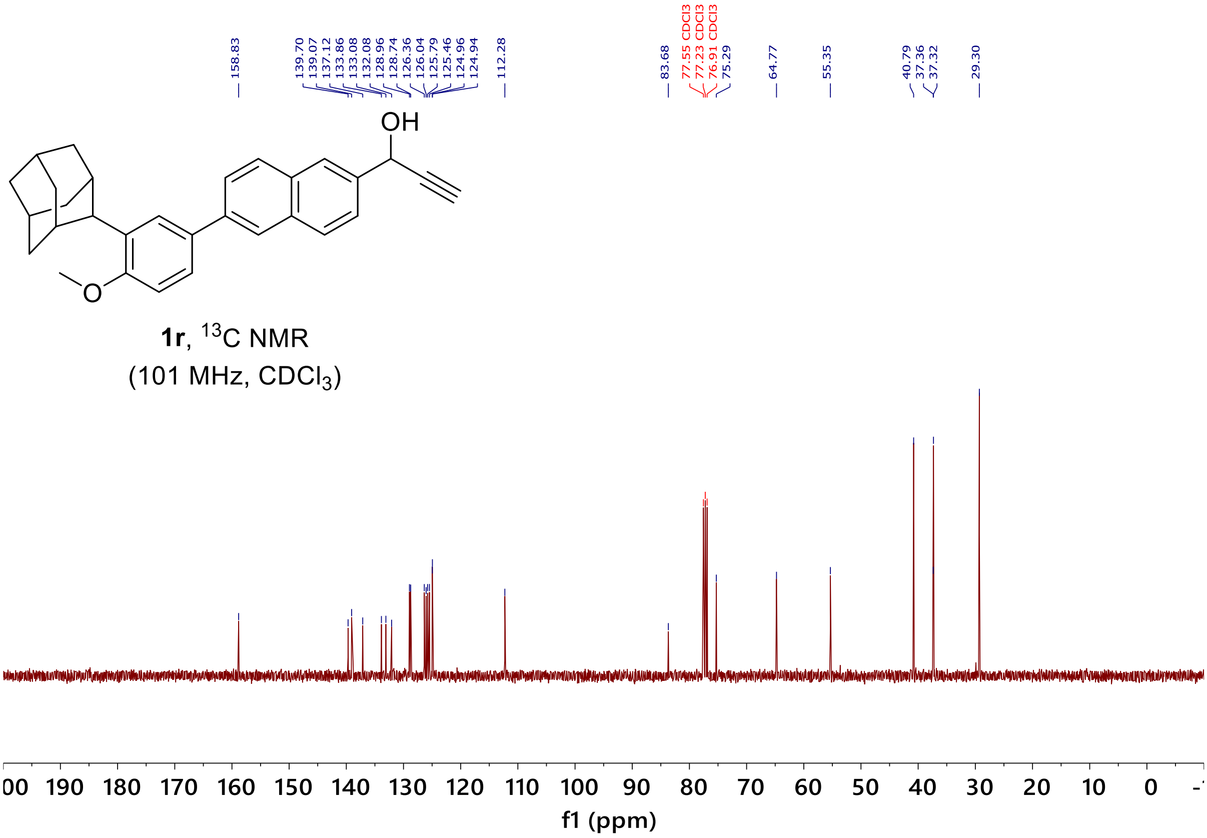

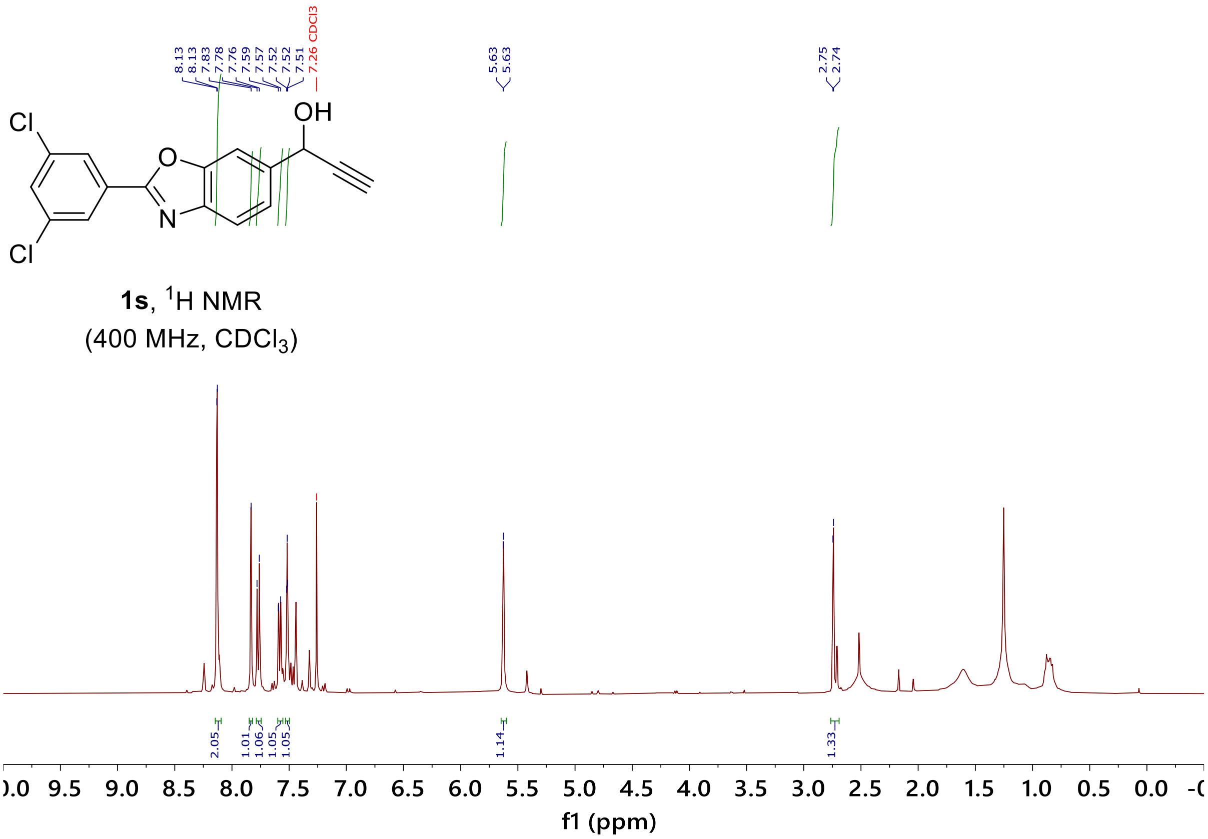

1s, ^{1}H NMR
(400 MHz, $CDCl_3$)

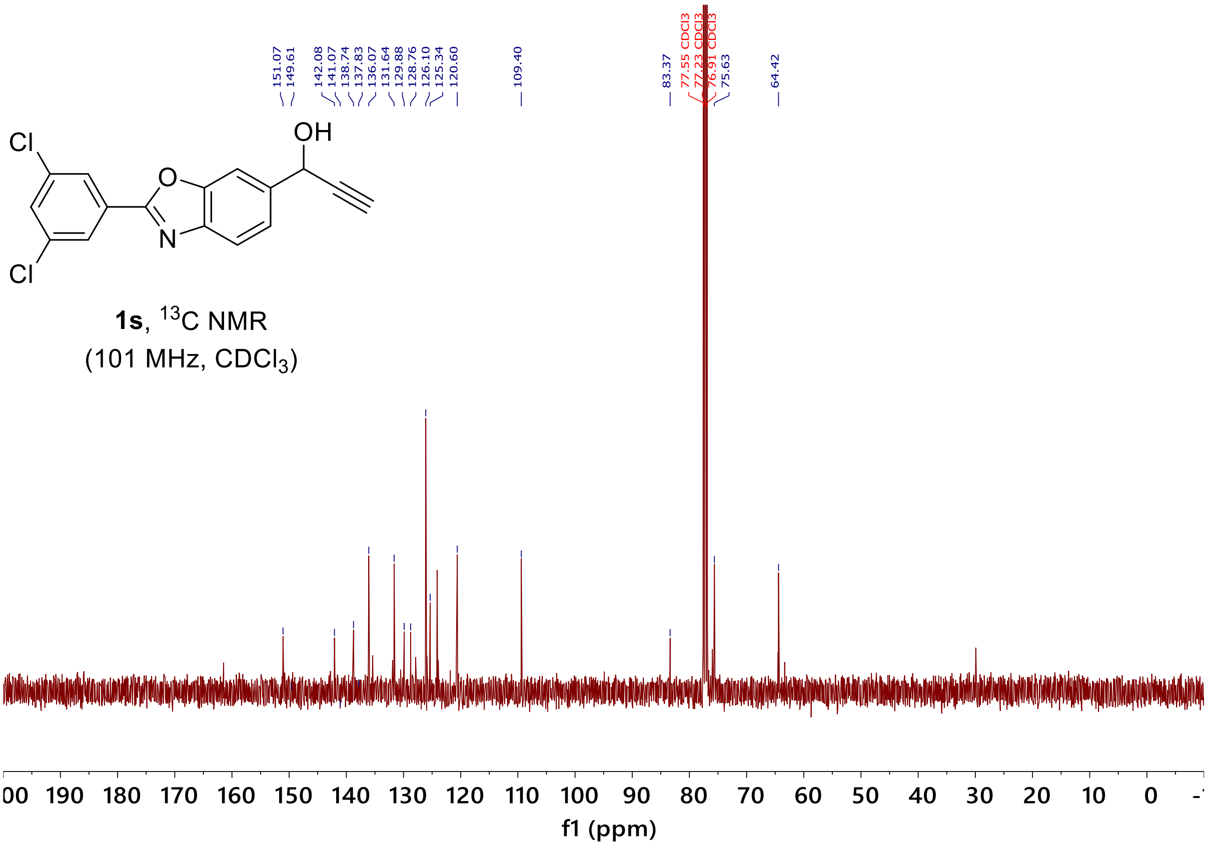

1s, ^{13}C NMR
(101 MHz, $CDCl_3$)

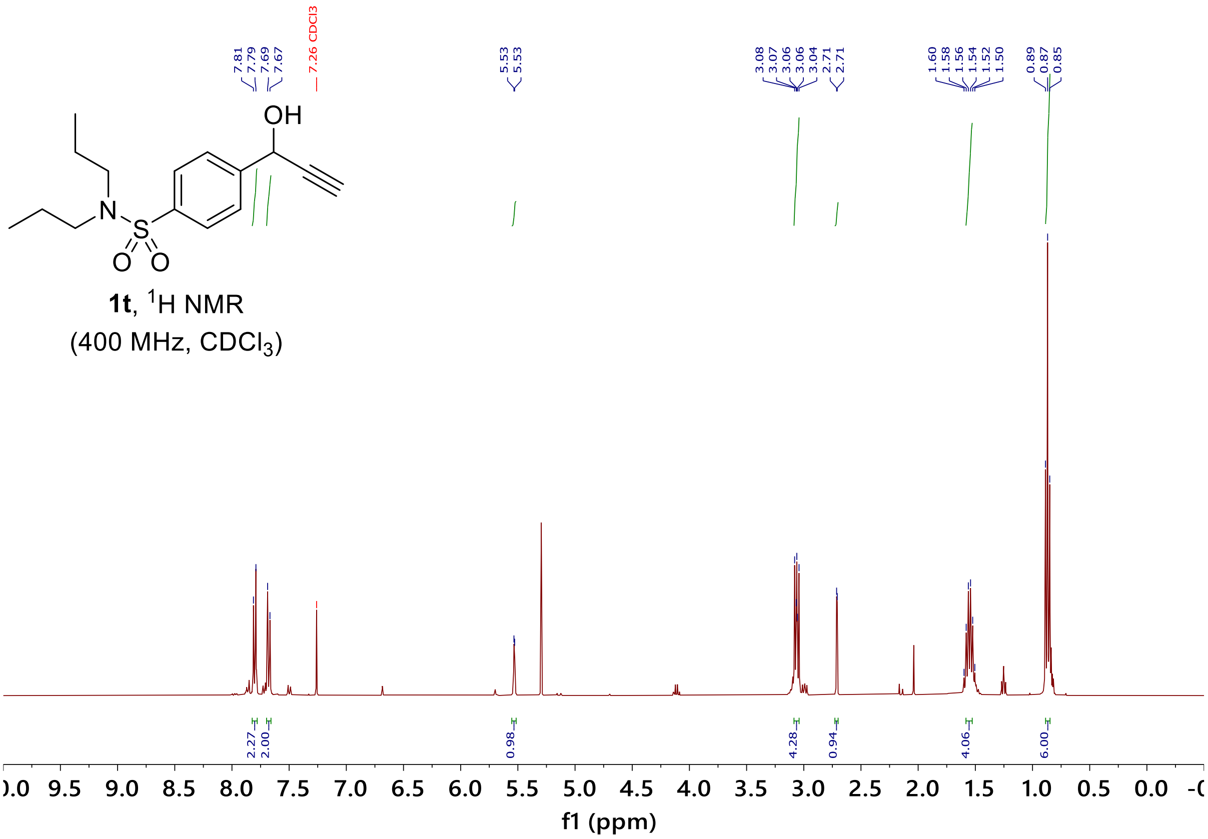

1t, ^{1}H NMR (400 MHz, $CDCl_3$)

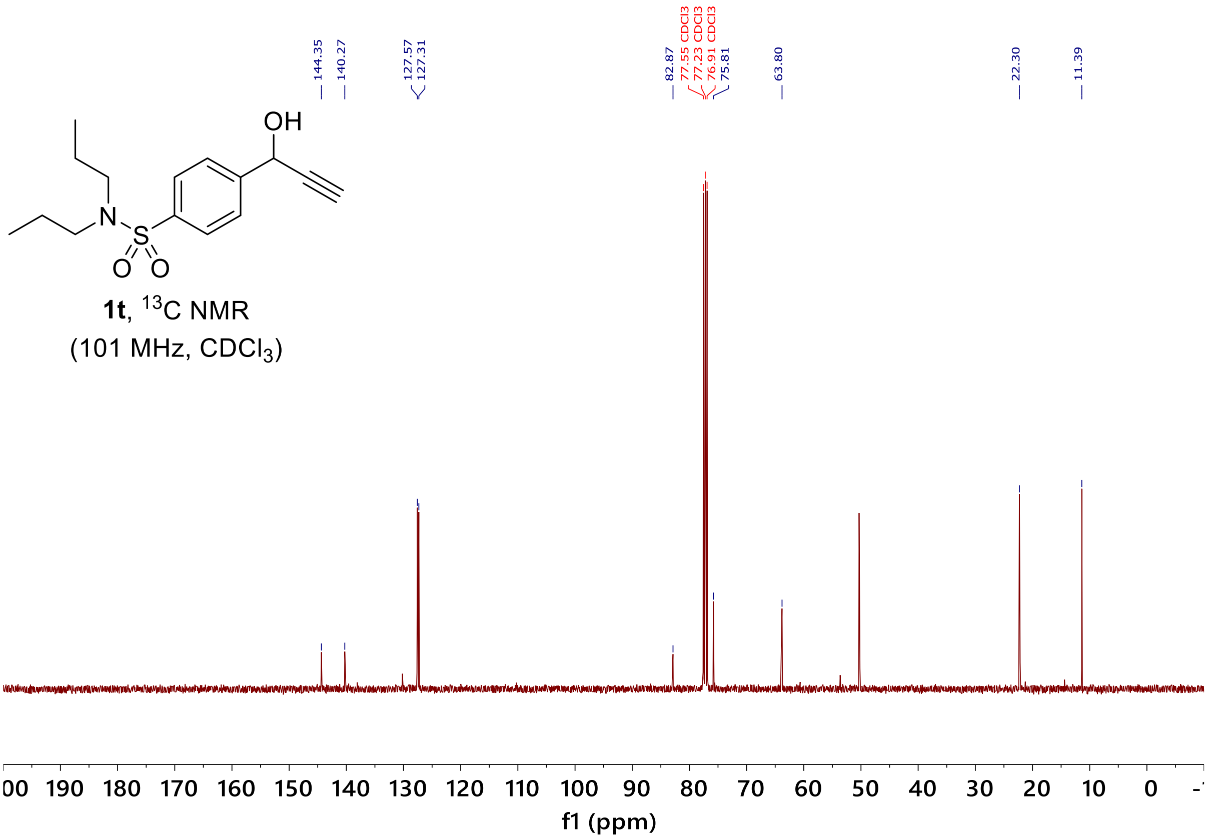

1t, ^{13}C NMR (101 MHz, $CDCl_3$)

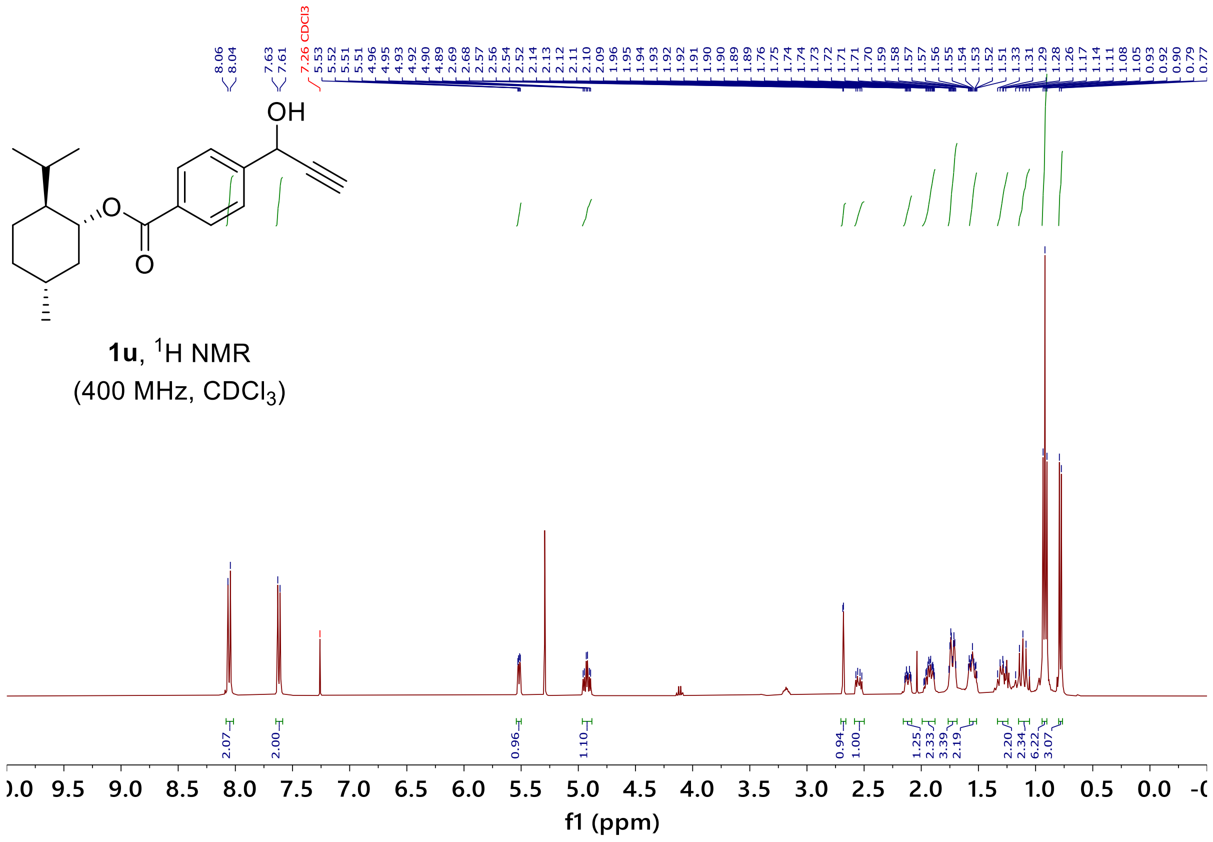
8.06
8.04
7.63
7.61
7.26 CDCl3
5.53
5.52
5.51
4.96
4.95
4.93
4.92
4.90
4.89
2.69
2.68
2.57
2.56
2.54
2.52
2.14
2.13
2.12
2.11
2.10
2.09
1.96
1.95
1.94
1.93
1.92
1.92
1.91
1.90
1.90
1.89
1.89
1.76
1.75
1.74
1.74
1.73
1.72
1.71
1.71
1.70
1.59
1.58
1.57
1.57
1.56
1.55
1.54
1.53
1.52
1.51
1.33
1.31
1.29
1.28
1.26
1.17
1.14
1.11
1.08
1.05
0.93
0.92
0.90
0.79
0.77
OH
O
O
1u, 1H NMR
(400 MHz, CDCl3)
2.07
2.00
0.96
1.10
0.94
1.00
1.25
2.33
3.39
2.19
2.20
2.34
6.22
3.07
f1 (ppm)

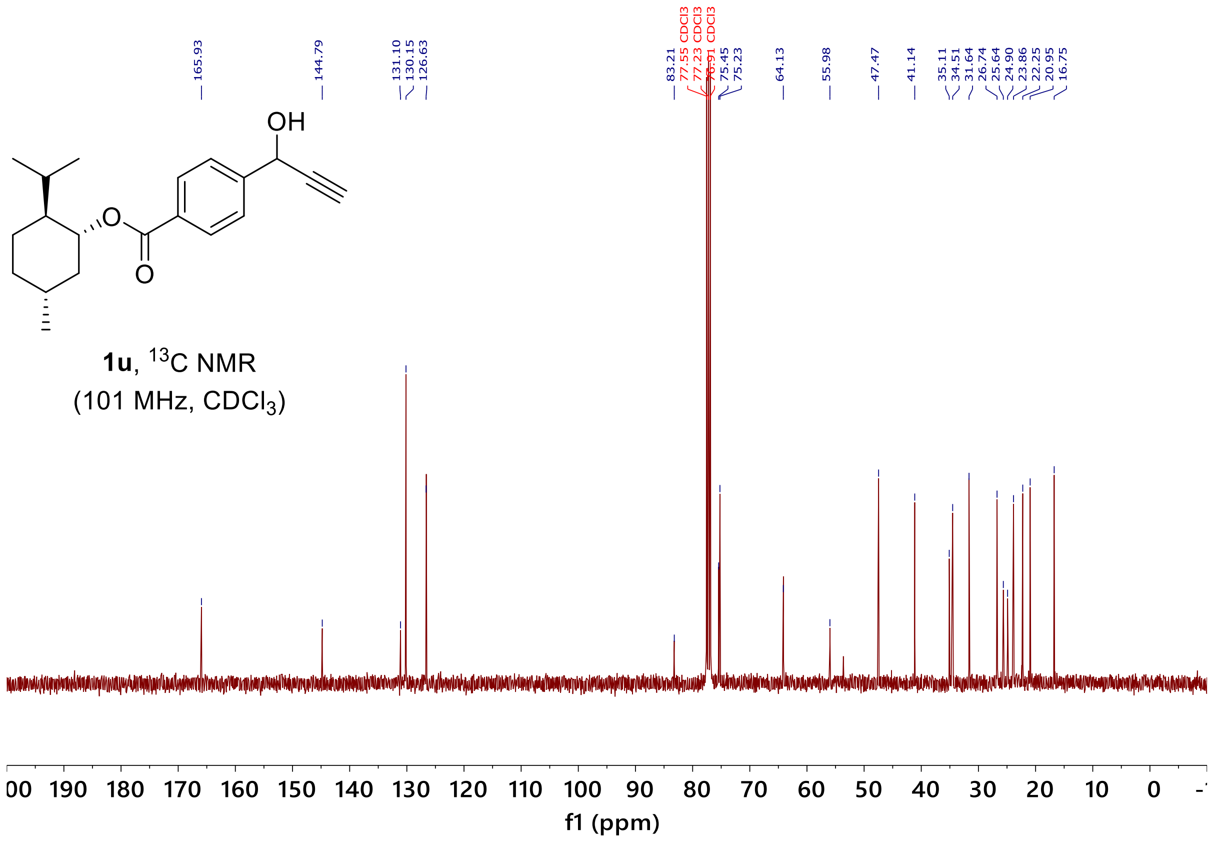
165.93
144.79
131.10
130.15
126.63
83.21
77.55 CDCl3
77.23 CDCl3
76.91 CDCl3
75.45
75.23
64.13
55.98
47.47
41.14
35.11
34.51
31.64
26.74
25.64
24.90
23.86
22.25
20.95
16.75
OH
O
O
1u, 13C NMR
(101 MHz, CDCl3)
f1 (ppm)

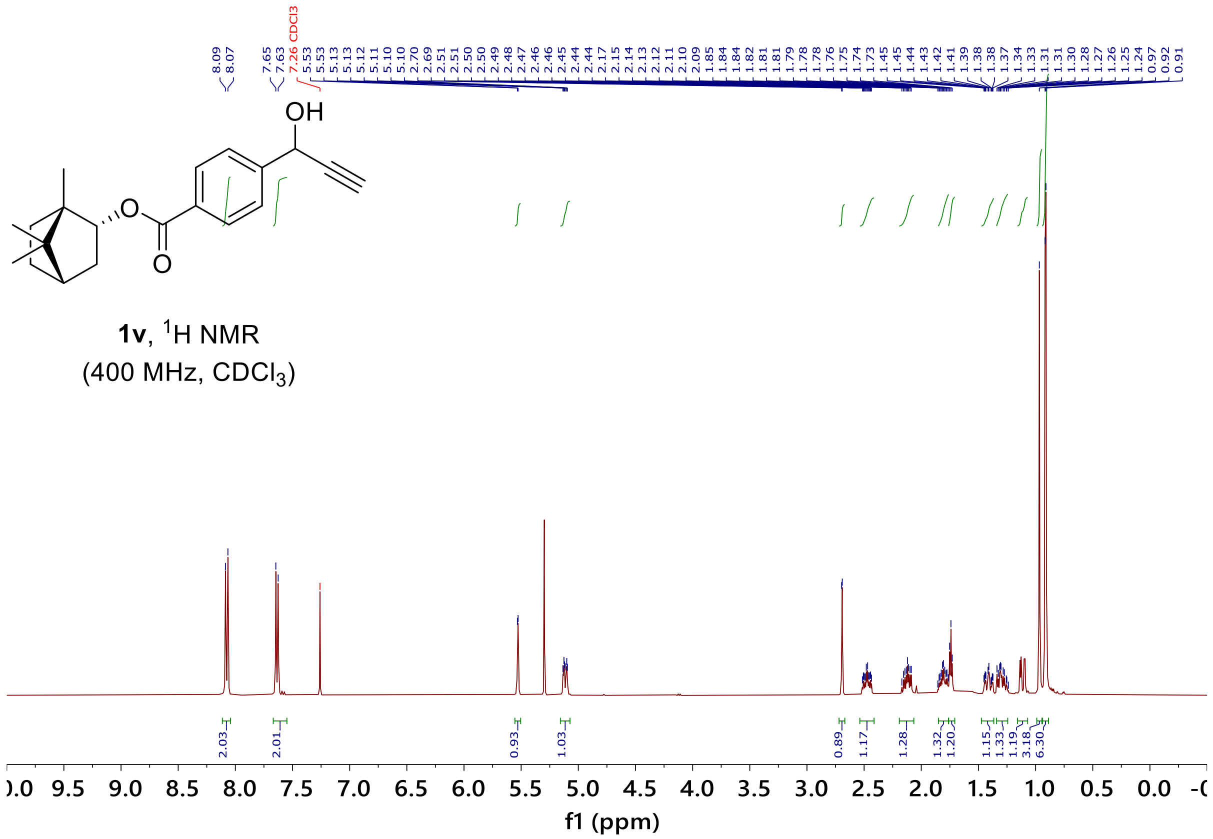

1v, ^{1}H NMR
(400 MHz, $CDCl_3$)

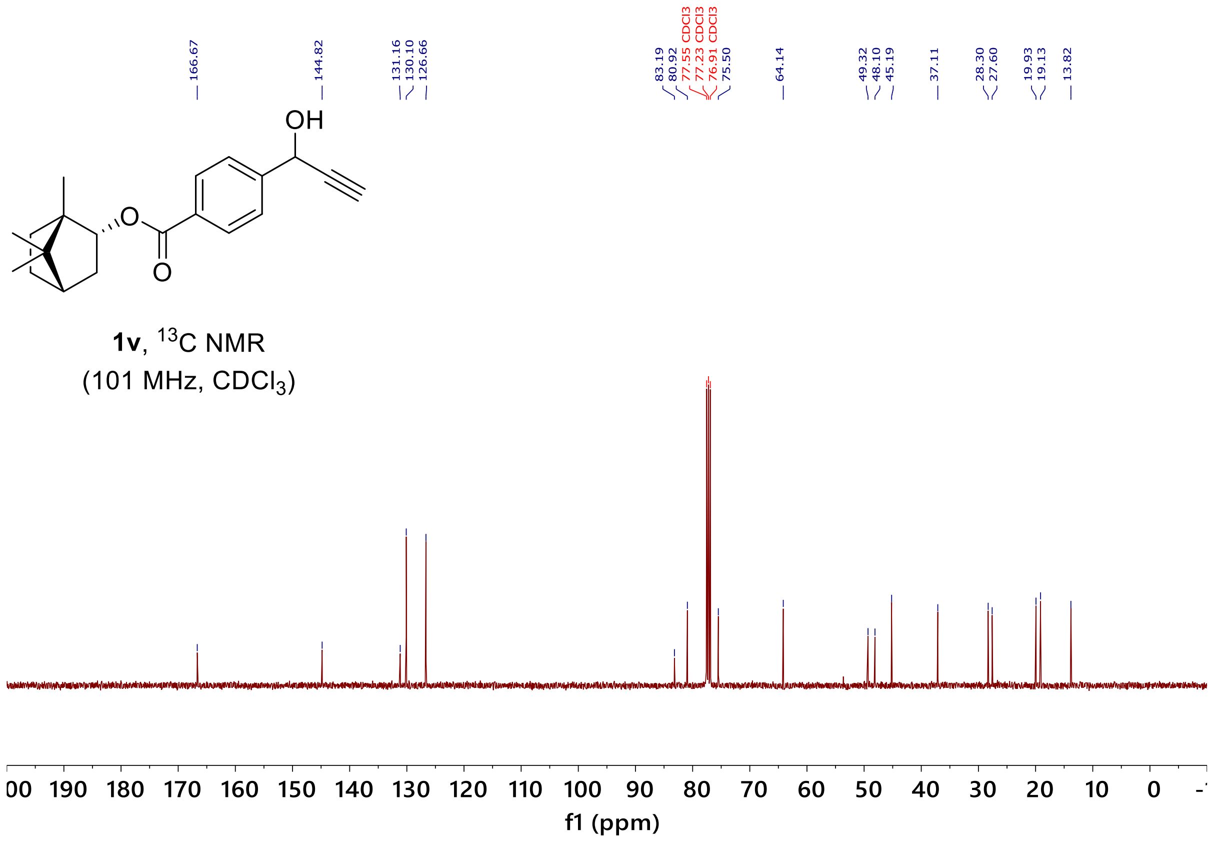

1v, ^{13}C NMR
(101 MHz, $CDCl_3$)

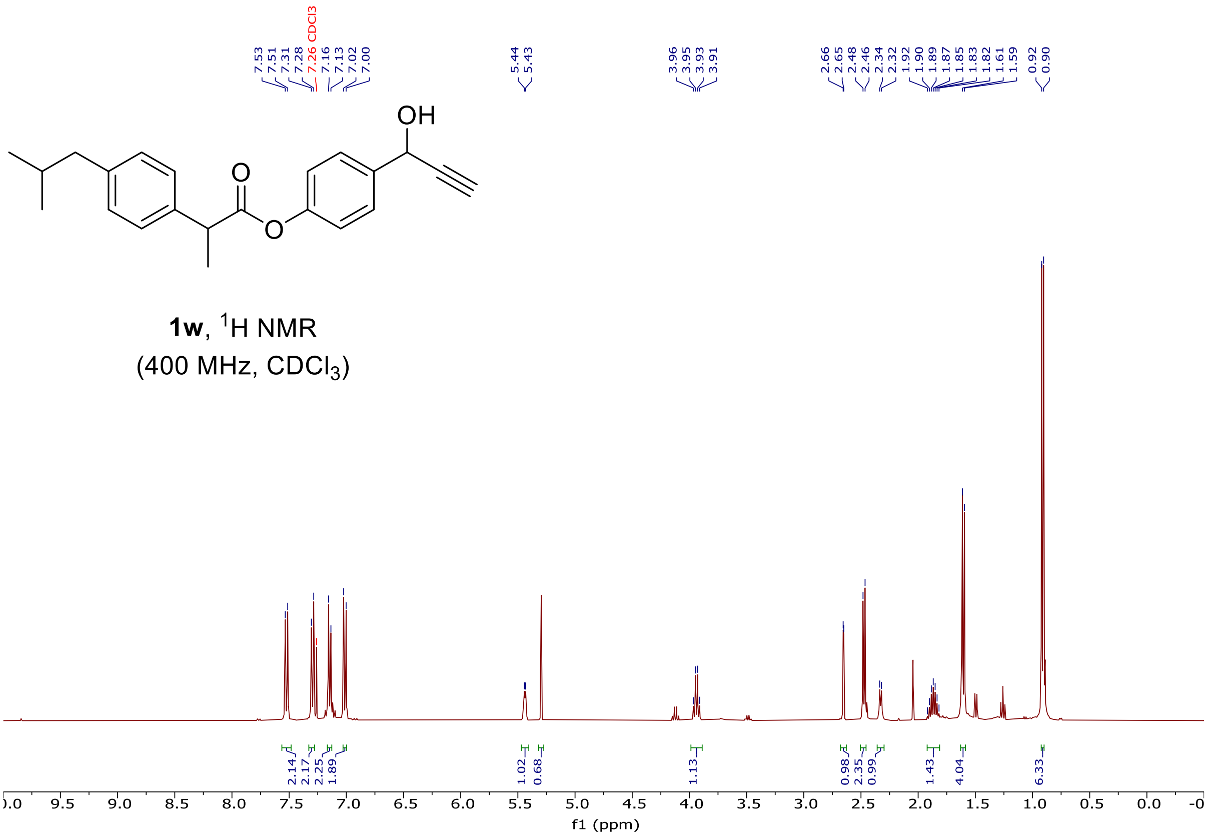
7.53
7.51
7.31
7.28
7.26 CDCl3
7.16
7.13
7.02
7.00
5.44
5.43
3.96
3.95
3.93
3.91
2.66
2.65
2.48
2.46
2.34
2.32
1.92
1.90
1.89
1.87
1.85
1.83
1.82
1.61
1.59
0.92
0.90
OH
O
O
1w, 1H NMR
(400 MHz, CDCl3)
2.14
2.17
2.25
1.89
1.02
0.68
1.13
0.98
2.35
0.99
1.43
4.04
6.33
f1 (ppm)

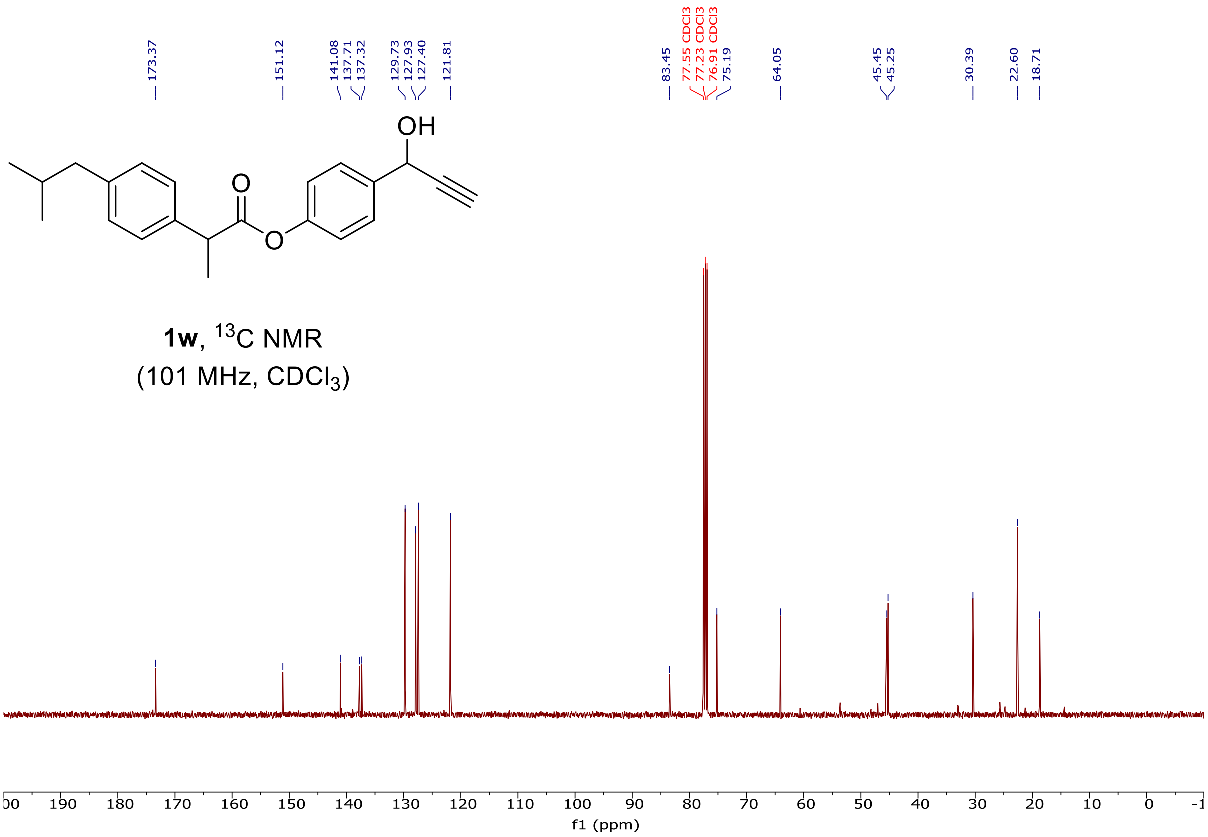
173.37
151.12
141.08
137.71
137.32
129.73
127.93
127.40
121.81
83.45
77.55 CDCl3
77.23 CDCl3
76.91 CDCl3
75.19
64.05
45.45
45.25
30.39
22.60
18.71
OH
O
O
1w, 13C NMR
(101 MHz, CDCl3)
f1 (ppm)

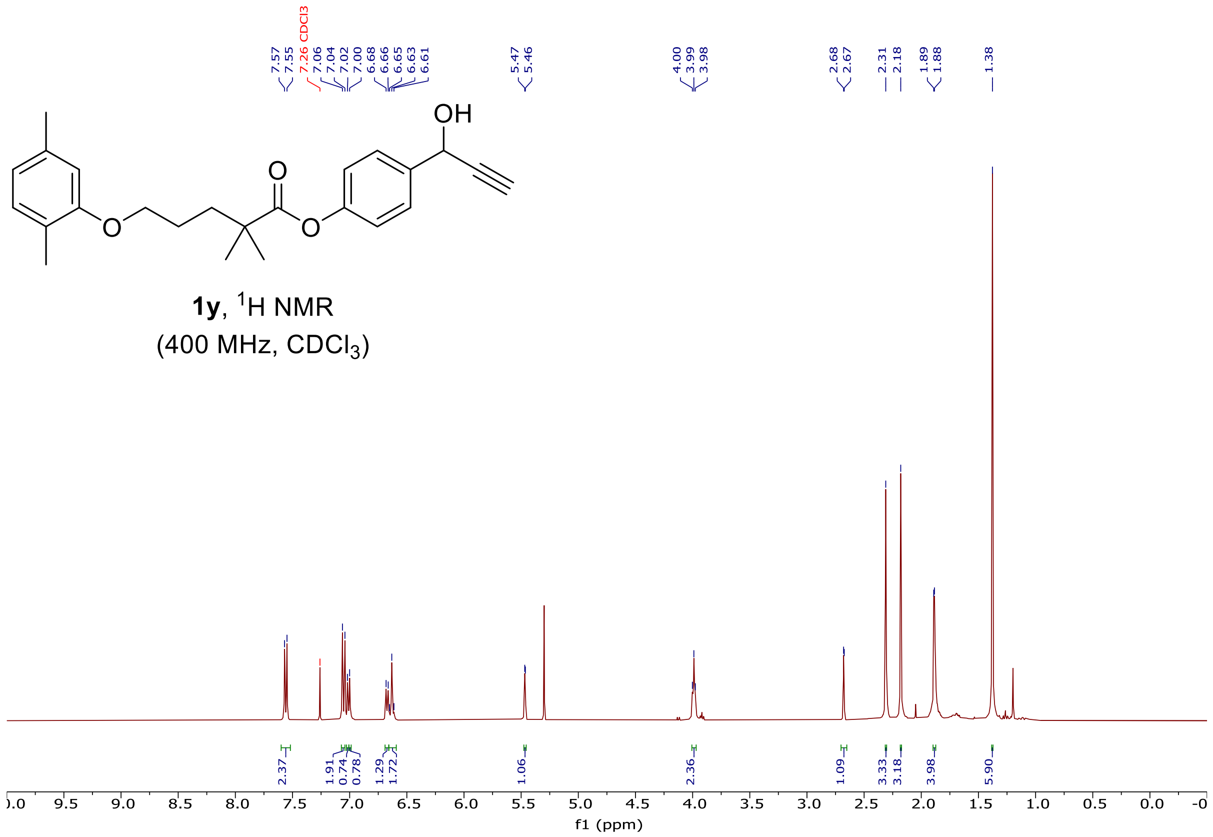

1y, ^{1}H NMR
(400 MHz, $CDCl_3$)

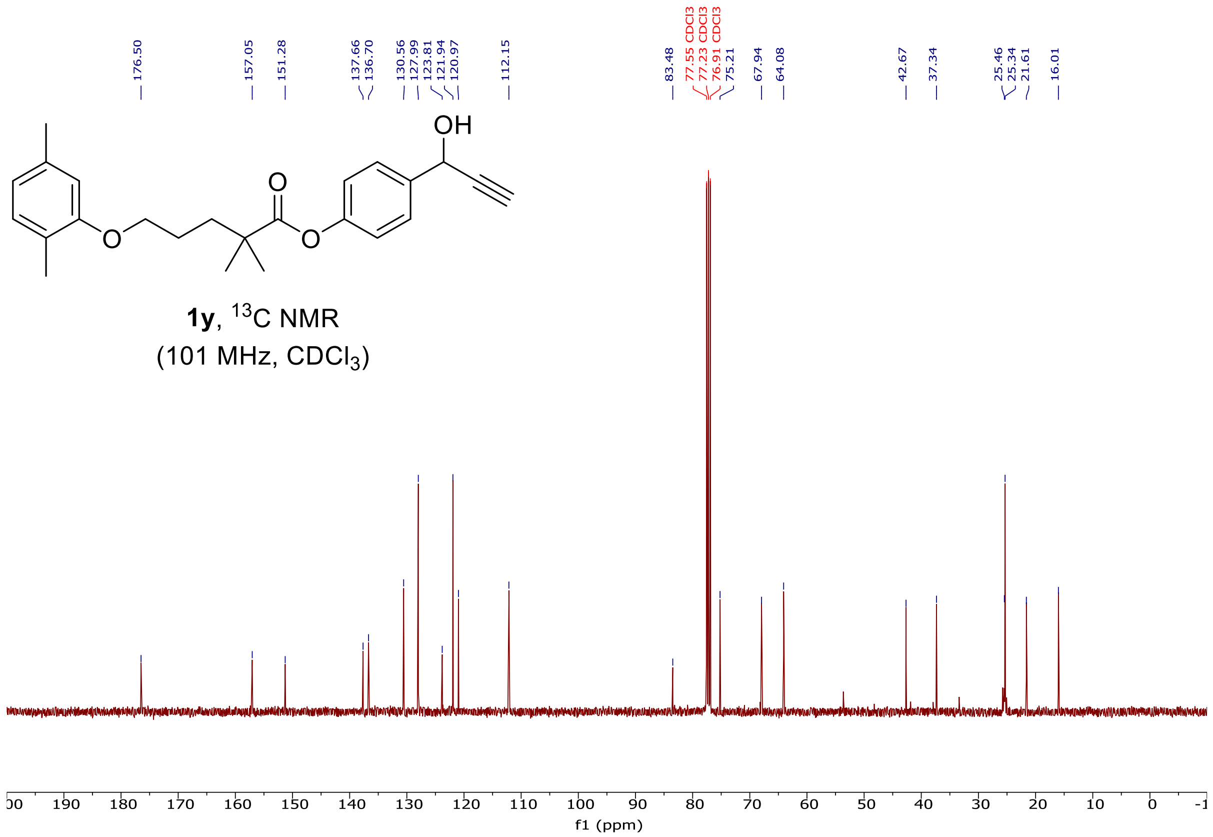

1y, ^{13}C NMR
(101 MHz, $CDCl_3$)

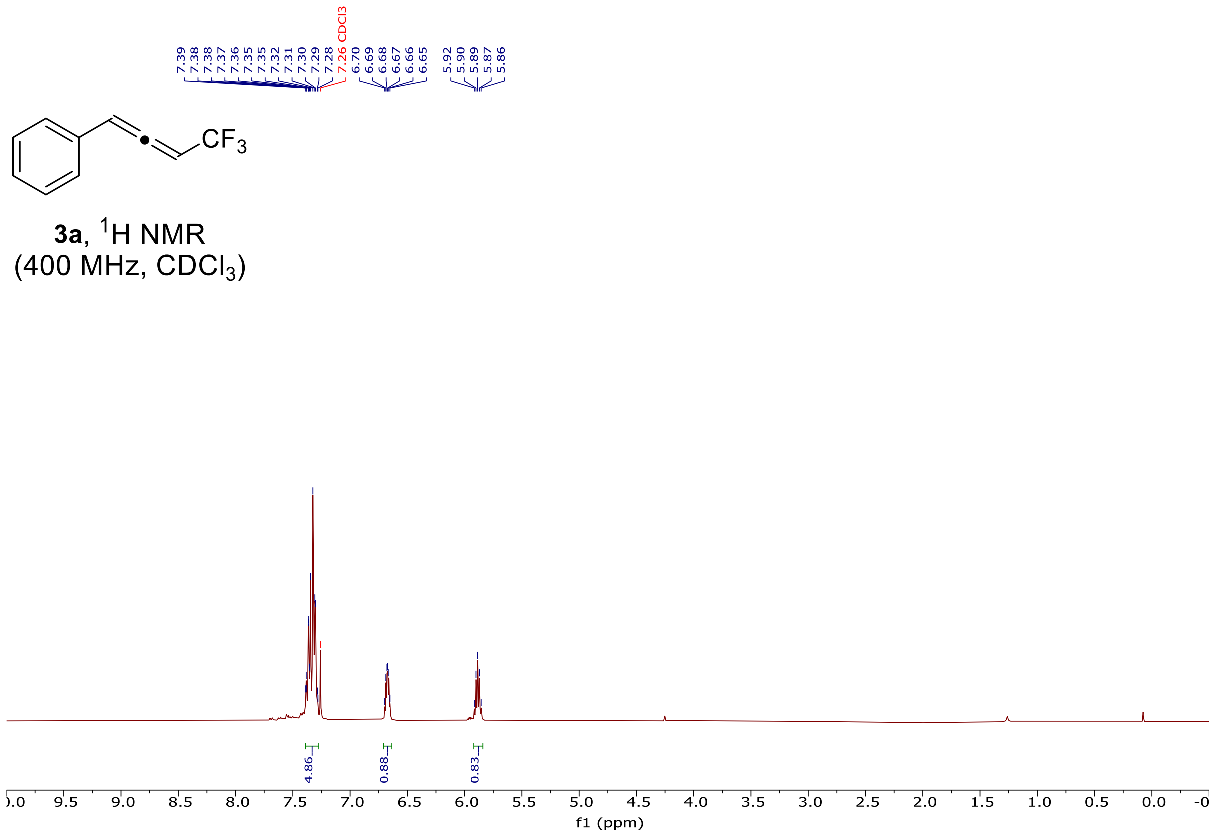
3a, ^{1}H NMR
(400 MHz, $CDCl_3$)
f1 (ppm)

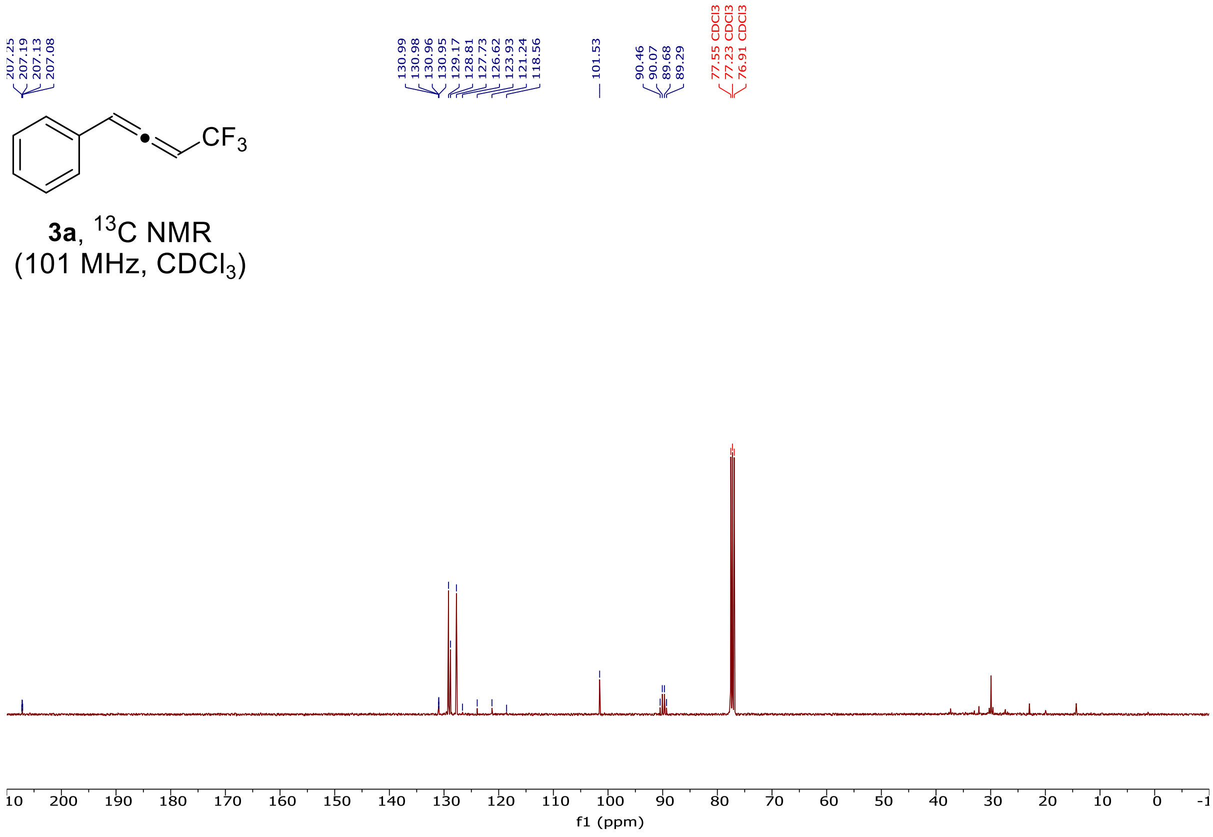
3a, ^{13}C NMR
(101 MHz, $CDCl_3$)
f1 (ppm)

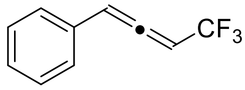

3a, ^{19}F NMR
(376 MHz, $CDCl_3$)

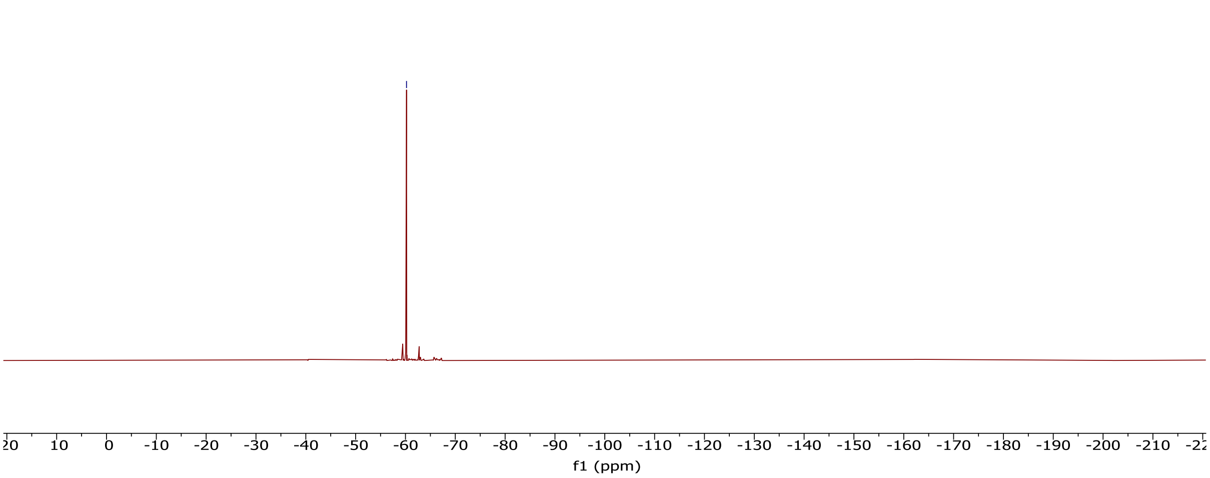

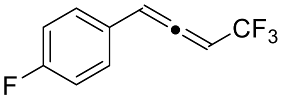

3b, ^{1}H NMR
(400 MHz, $CDCl_3$)
(in pentane residue)

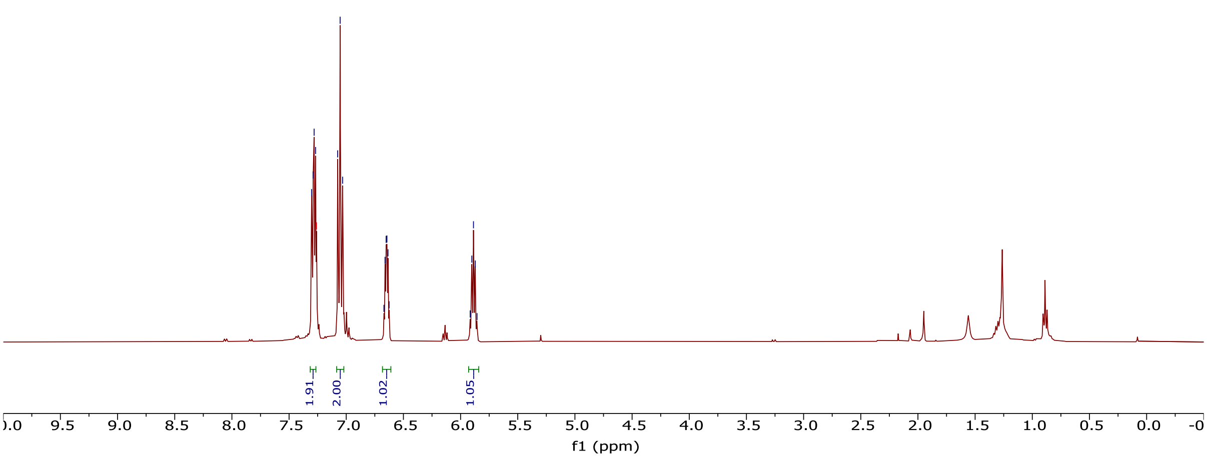

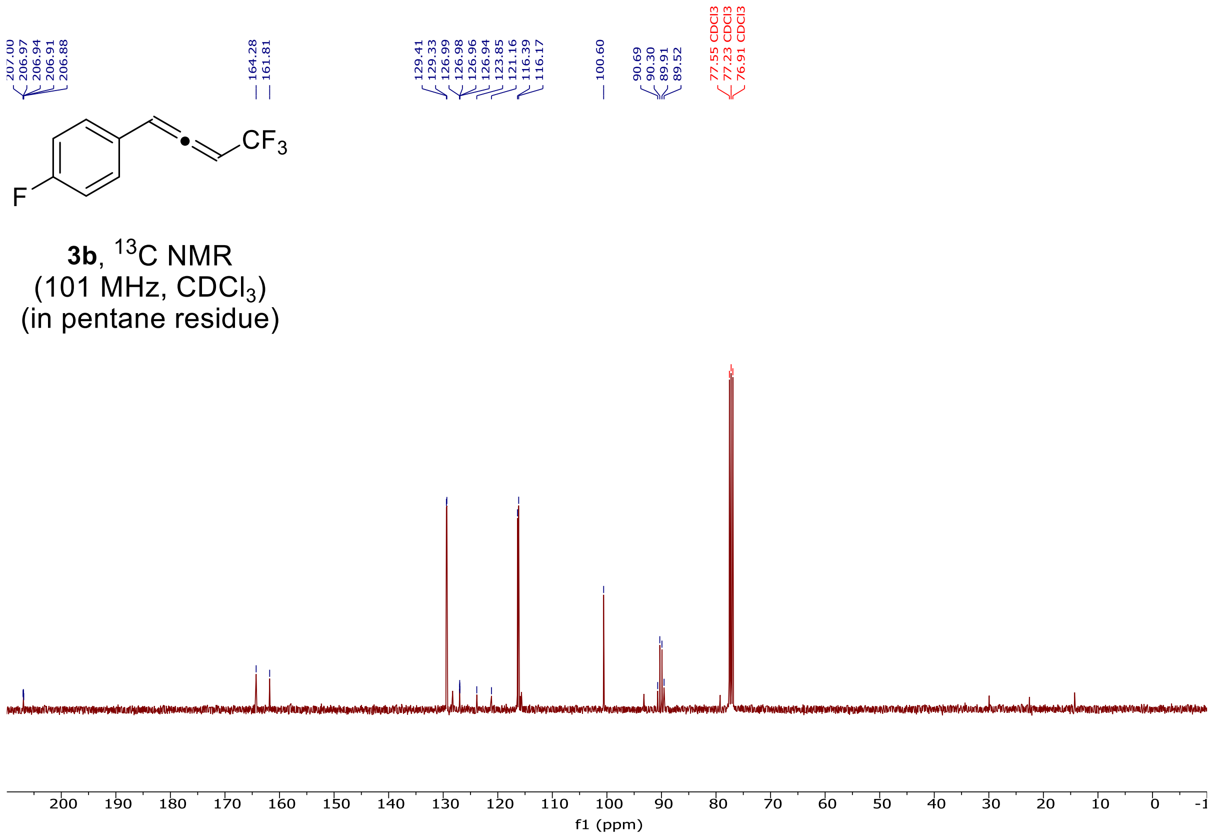

3b, ^{13}C NMR
(101 MHz, $CDCl_3$)
(in pentane residue)

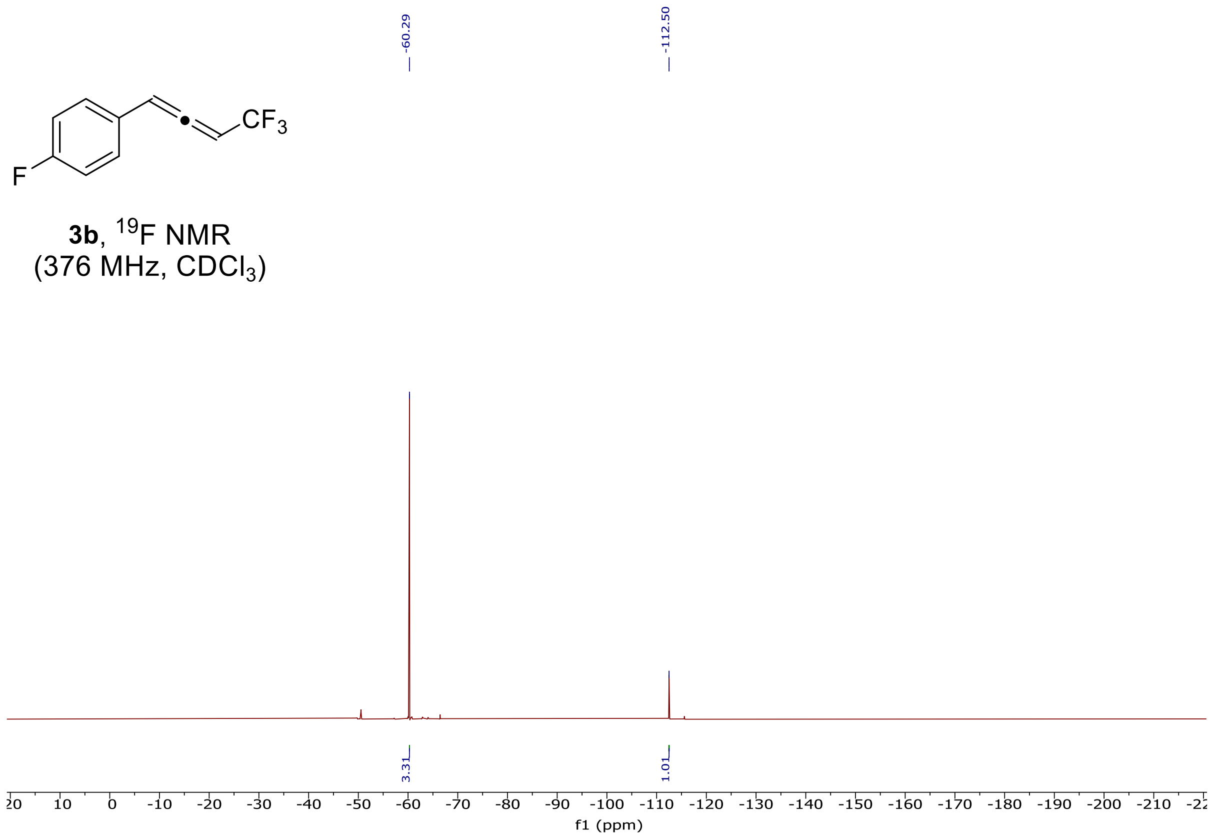

3b, ^{19}F NMR
(376 MHz, $CDCl_3$)

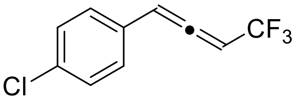

3c, ^{1}H NMR
(400 MHz, $CDCl_3$)
(in pentane residue)

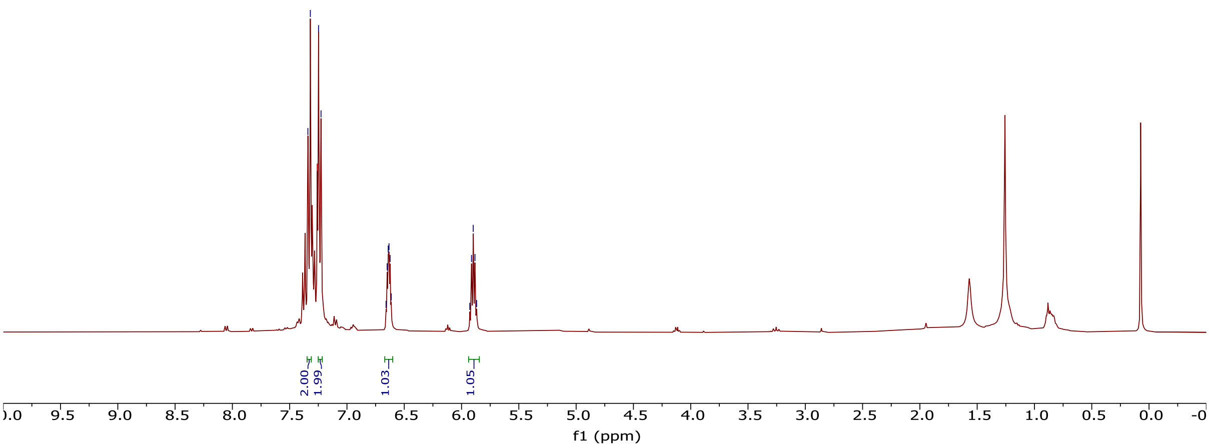

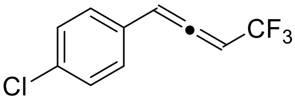

3c, ^{13}C NMR
(101 MHz, $CDCl_3$)
(in pentane residue)

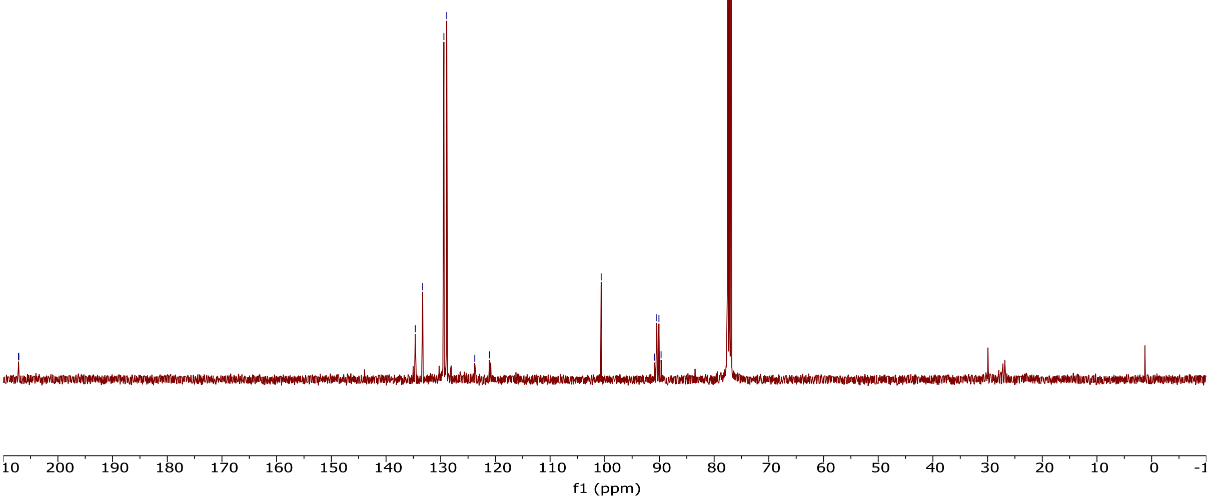

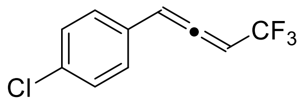

3c, ^{19}F NMR
(376 MHz, $CDCl_3$)

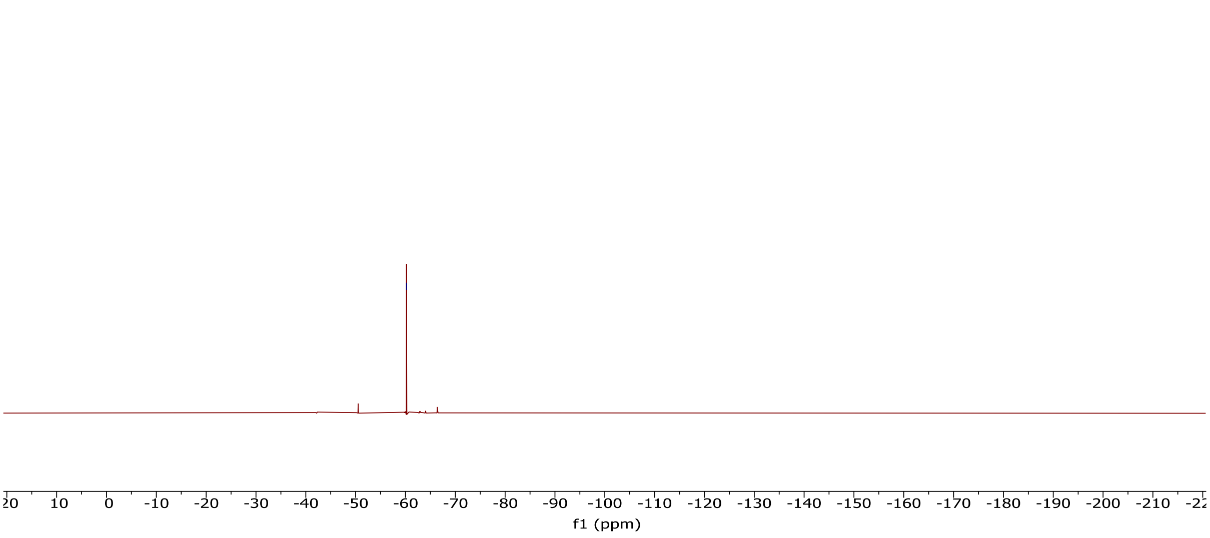

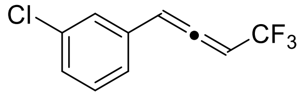

3d, ^{1}H NMR
(400 MHz, $CDCl_3$)
(in pentane residue)

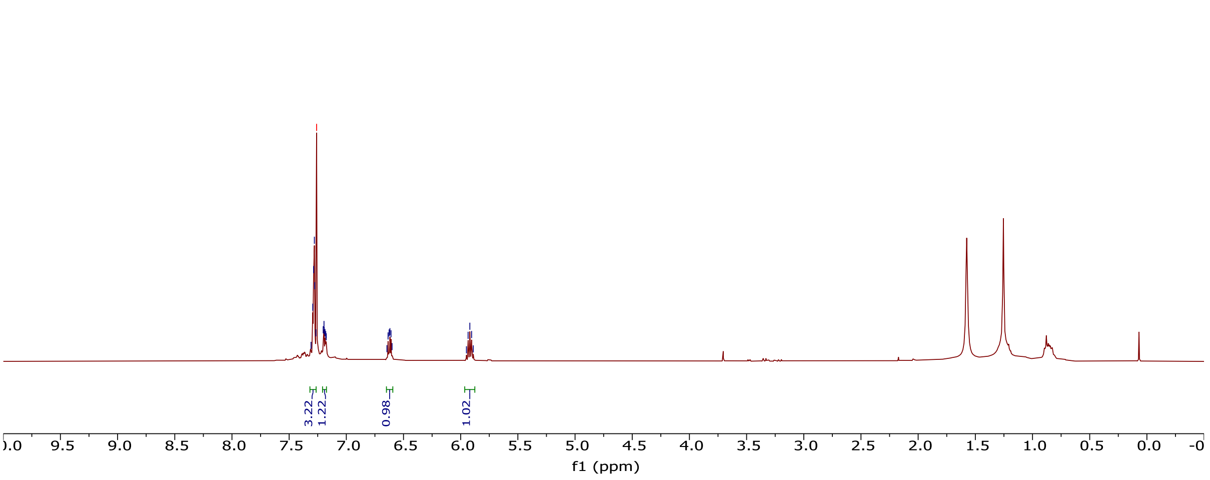

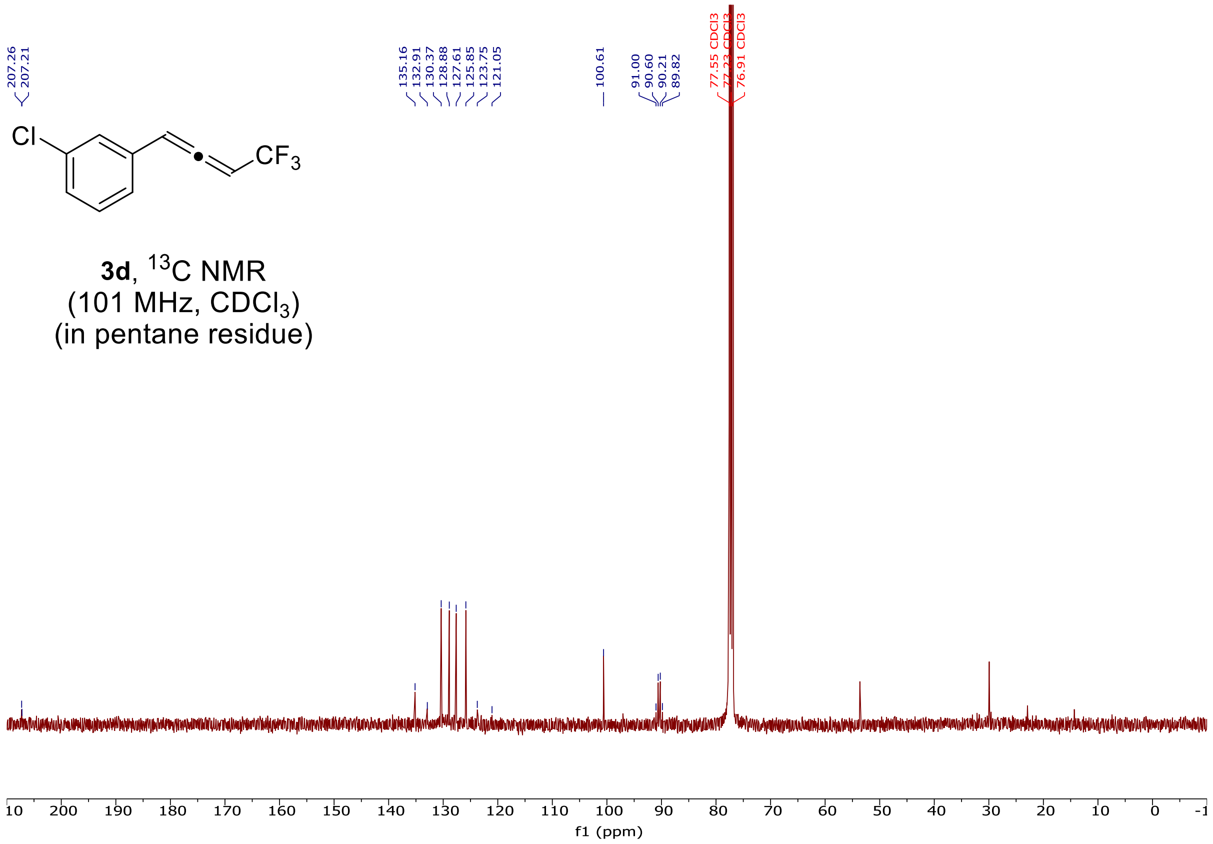
207.26
207.21
Cl
CF3
135.16
132.91
130.37
128.88
127.61
125.85
123.75
121.05
100.61
91.00
90.60
90.21
89.82
77.55 CDCl3
77.23 CDCl3
76.91 CDCl3
3d, 13C NMR
(101 MHz, CDCl3)
(in pentane residue)
f1 (ppm)

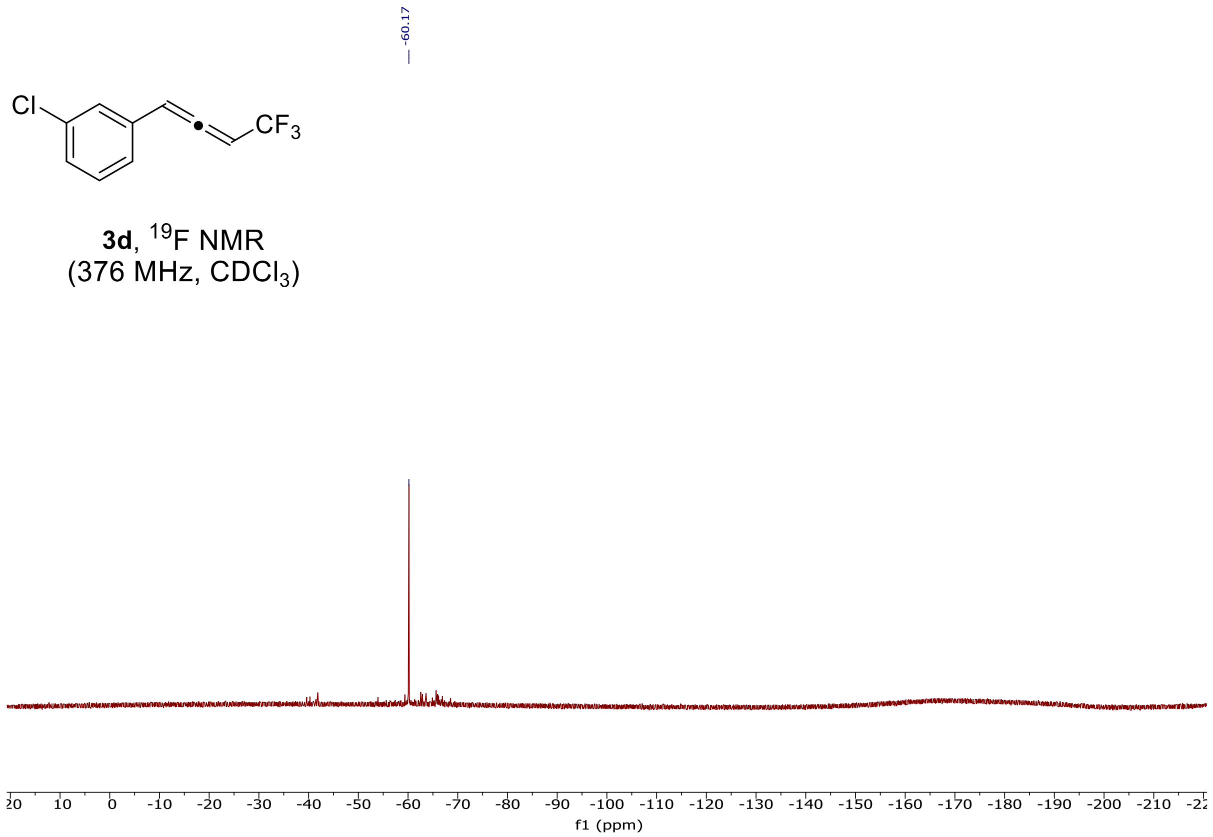
-60.17
Cl
CF3
3d, 19F NMR
(376 MHz, CDCl3)
f1 (ppm)

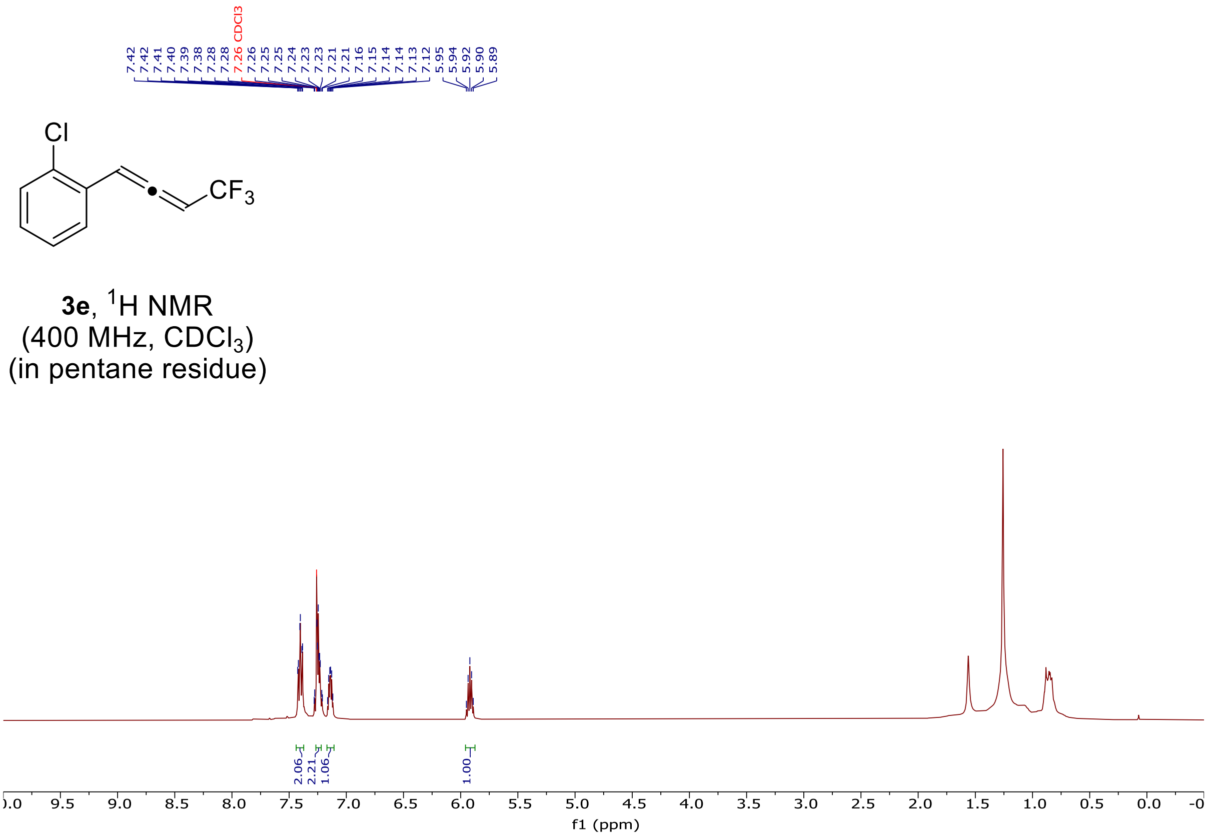

3e, ^{1}H NMR
(400 MHz, $CDCl_3$)
(in pentane residue)

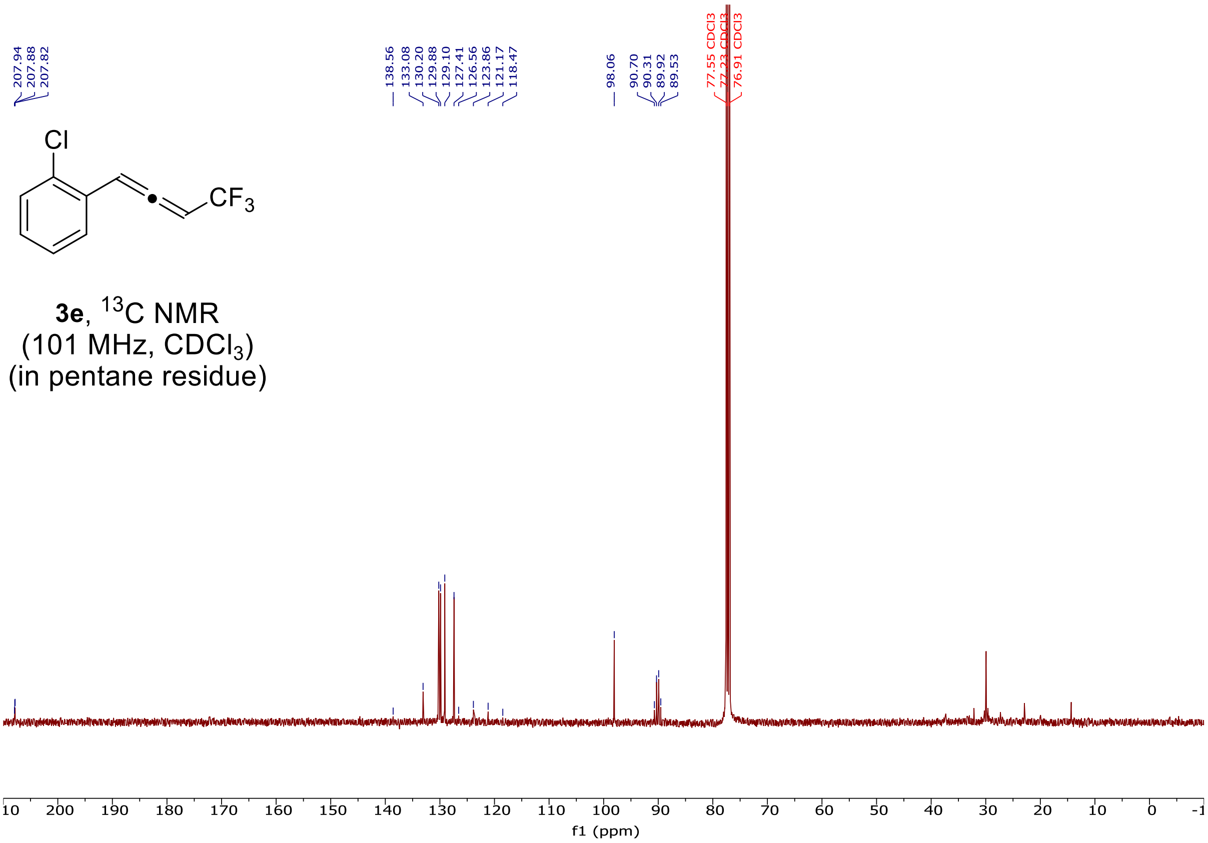

3e, ^{13}C NMR
(101 MHz, $CDCl_3$)
(in pentane residue)

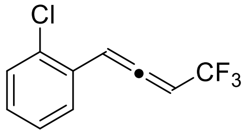

3e, ^{19}F NMR
(376 MHz, $CDCl_3$)

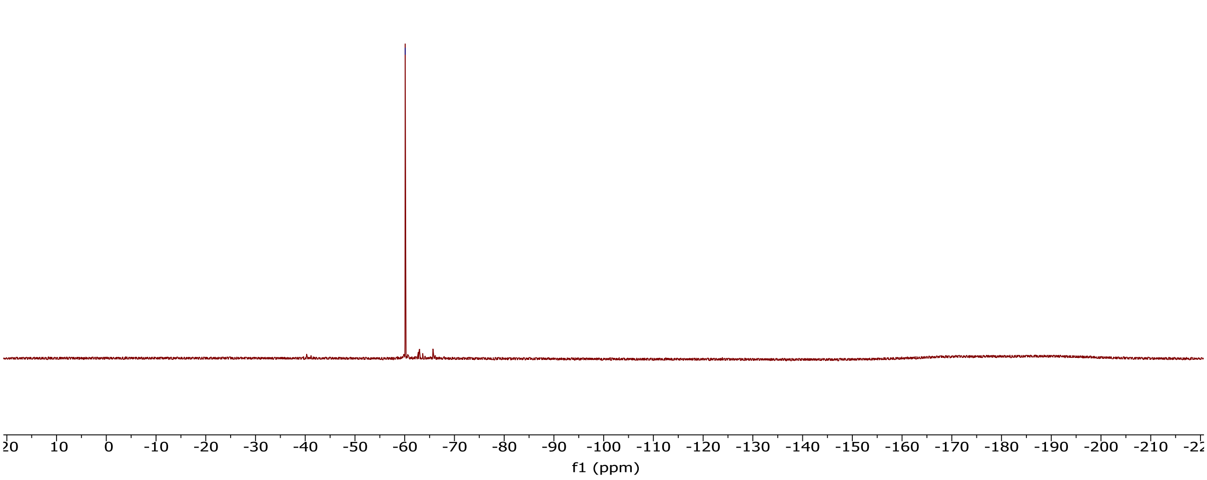

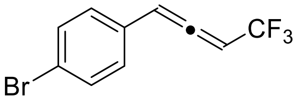

3f, ^{1}H NMR
(400 MHz, $CDCl_3$)
(in pentane residue)

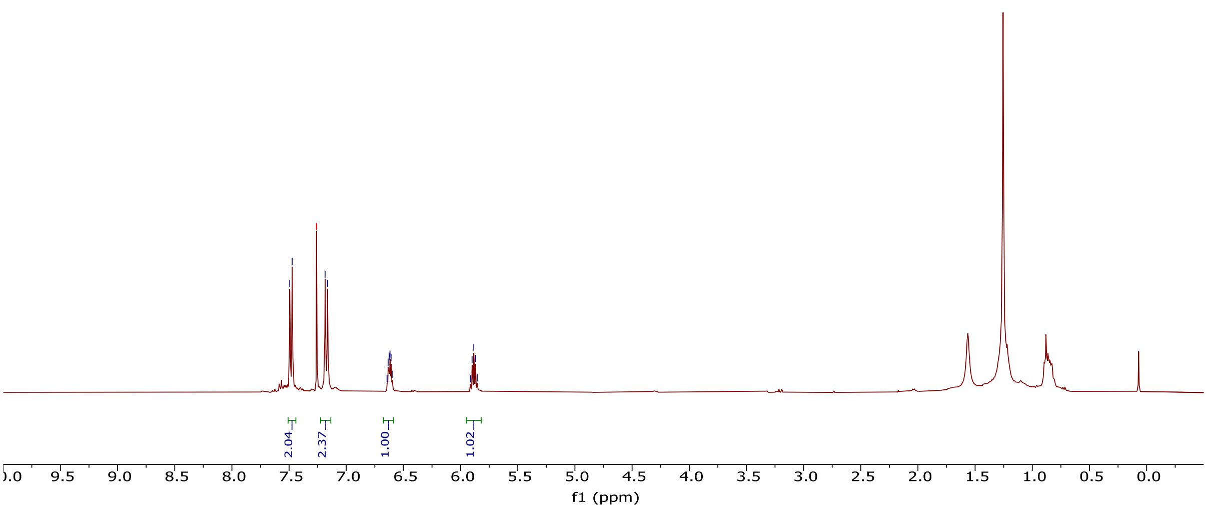

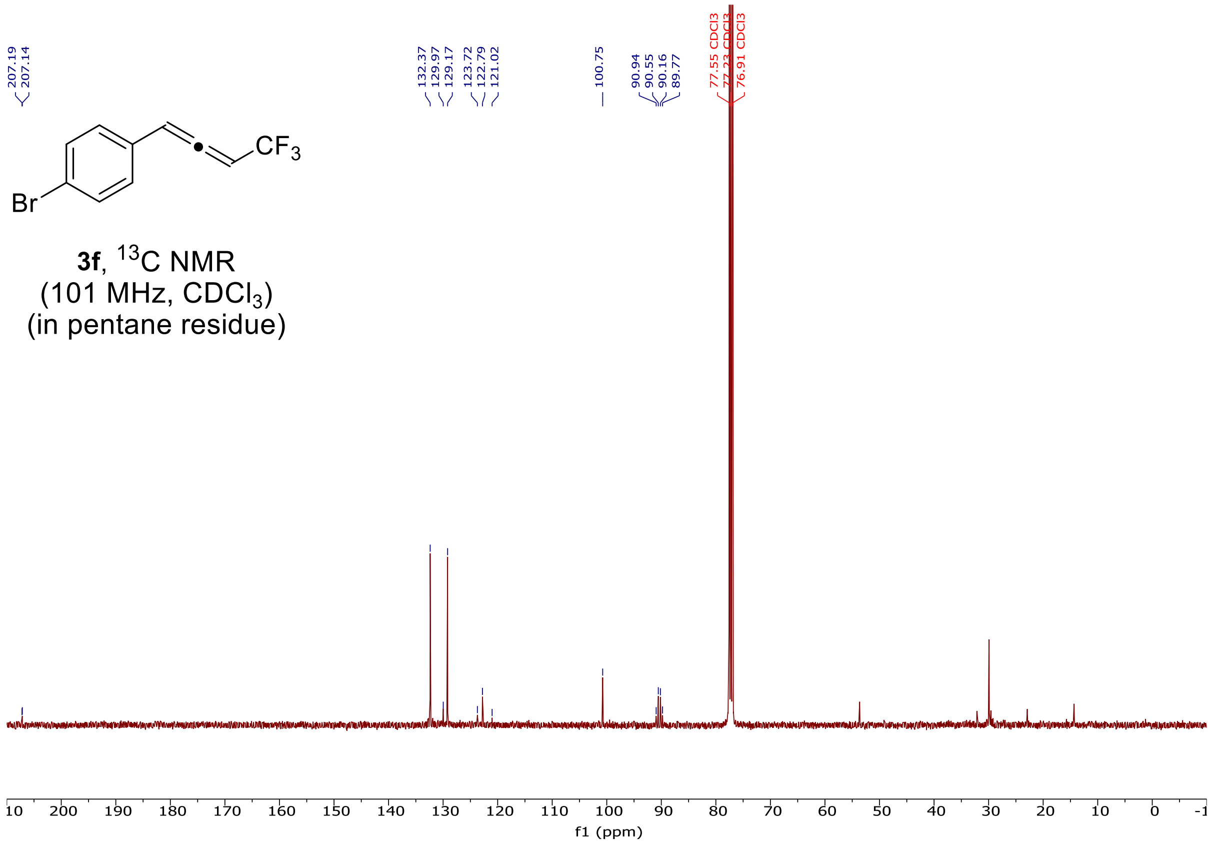
207.19
207.14
132.37
129.97
129.17
123.72
122.79
121.02
100.75
90.94
90.55
90.16
89.77
77.55 CDCl3
77.23 CDCl3
76.91 CDCl3
CF3
Br
3f, 13C NMR
(101 MHz, CDCl3)
(in pentane residue)
f1 (ppm)

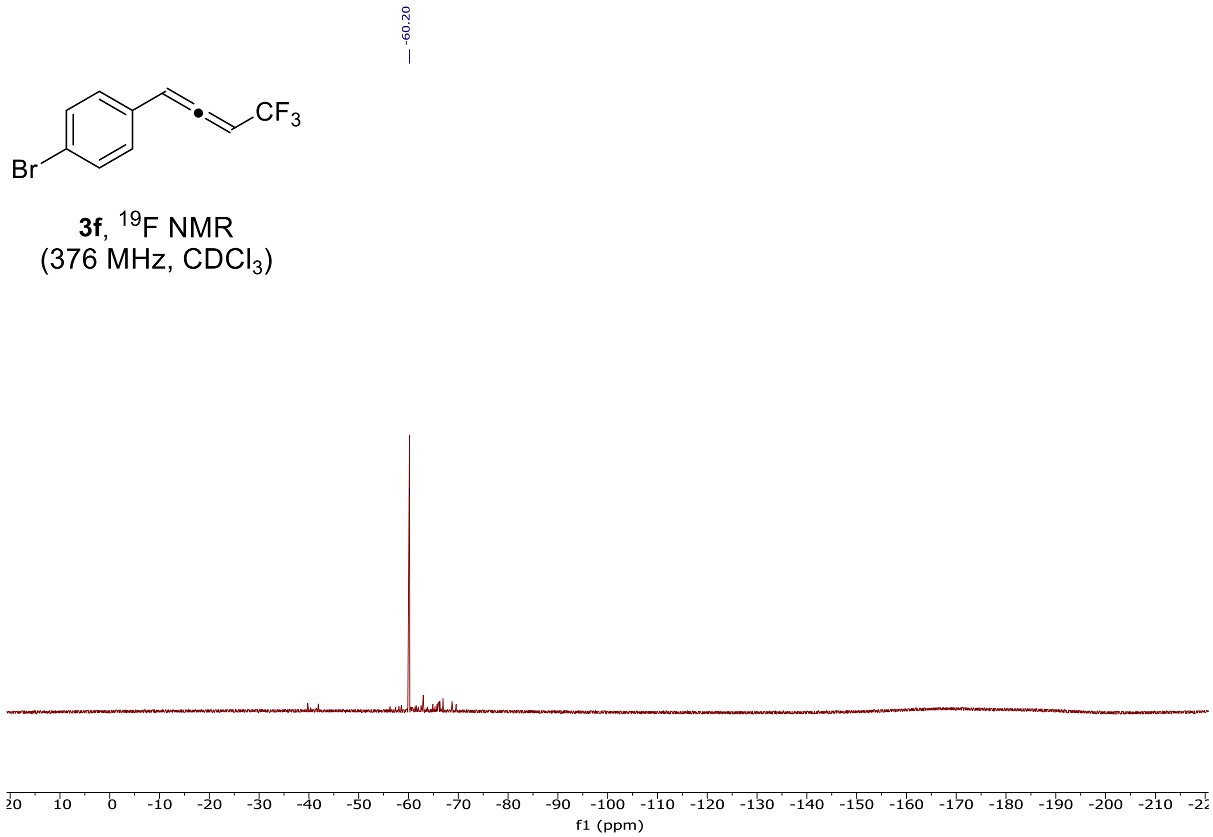
-60.20
CF3
Br
3f, 19F NMR
(376 MHz, CDCl3)
f1 (ppm)

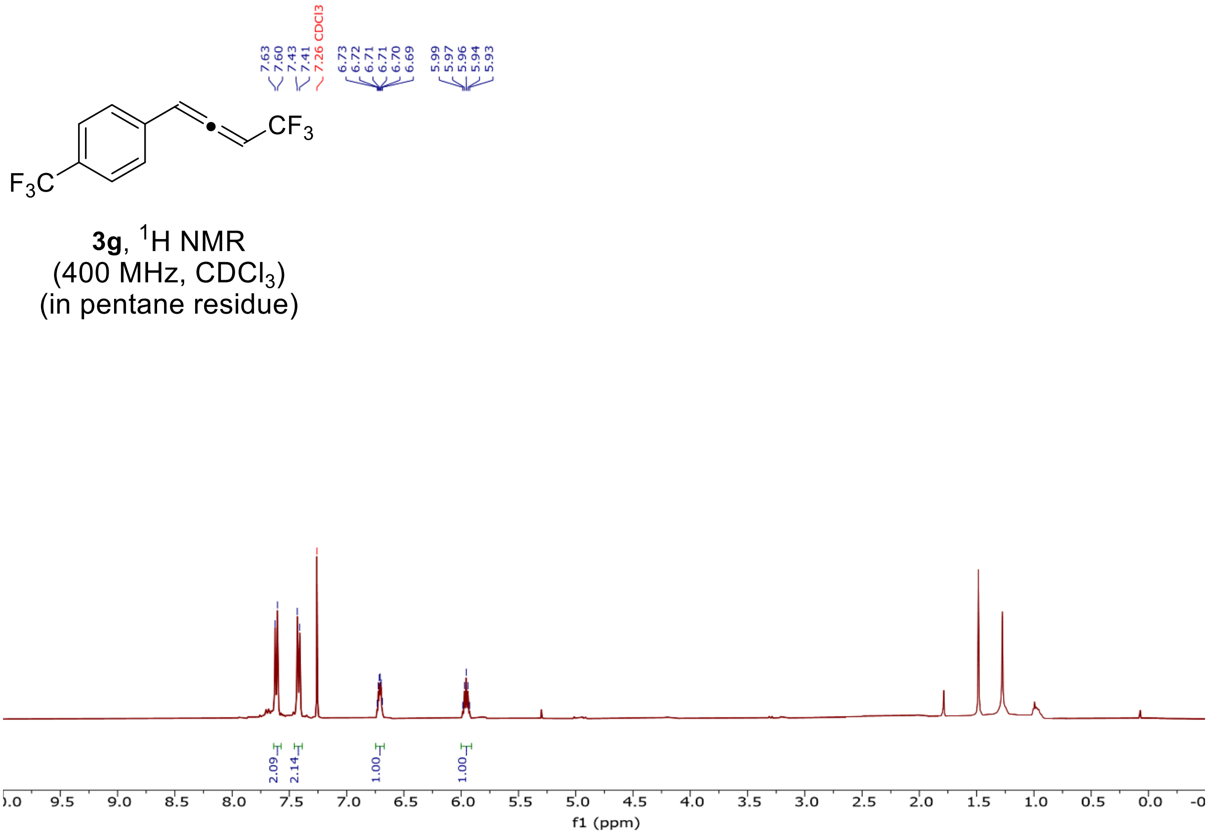

3g, ^{1}H NMR
(400 MHz, $CDCl_3$)
(in pentane residue)

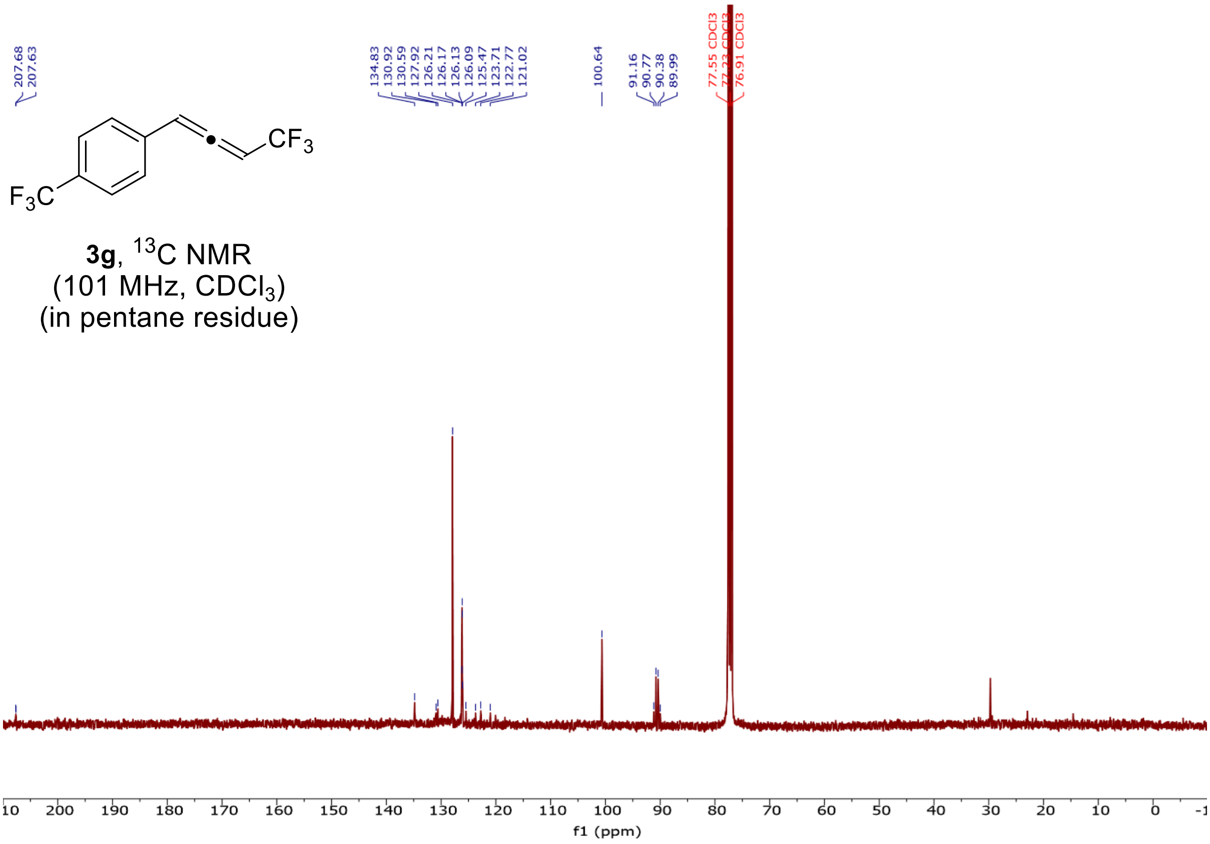

3g, ^{13}C NMR
(101 MHz, $CDCl_3$)
(in pentane residue)

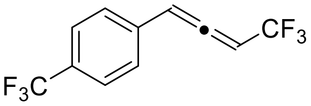

3g, ^{19}F NMR
(376 MHz, $CDCl_3$)

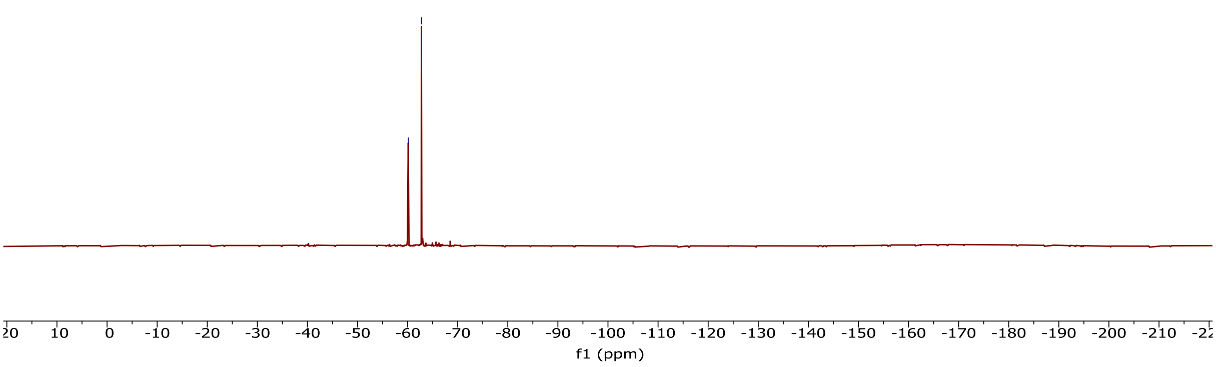

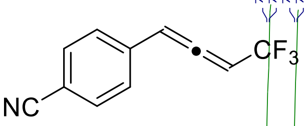

3h, ^{1}H NMR
(400 MHz, $CDCl_3$)
(in pentane residue)

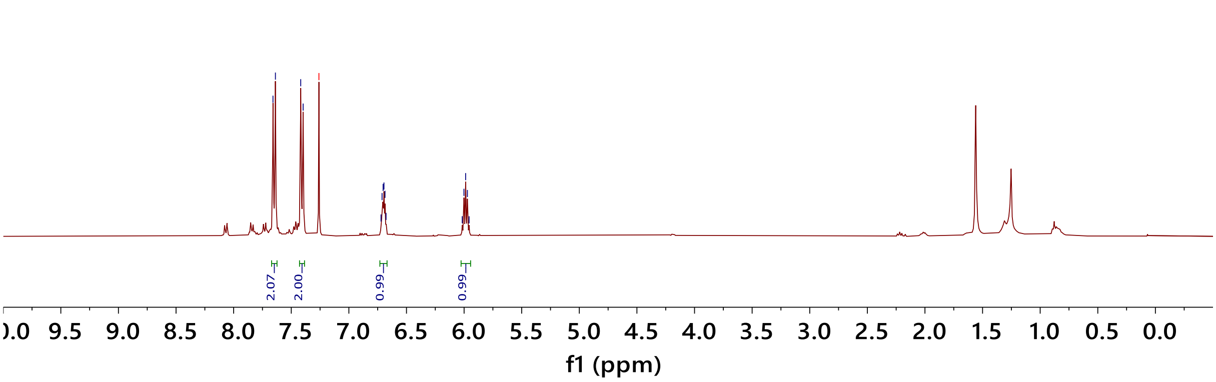

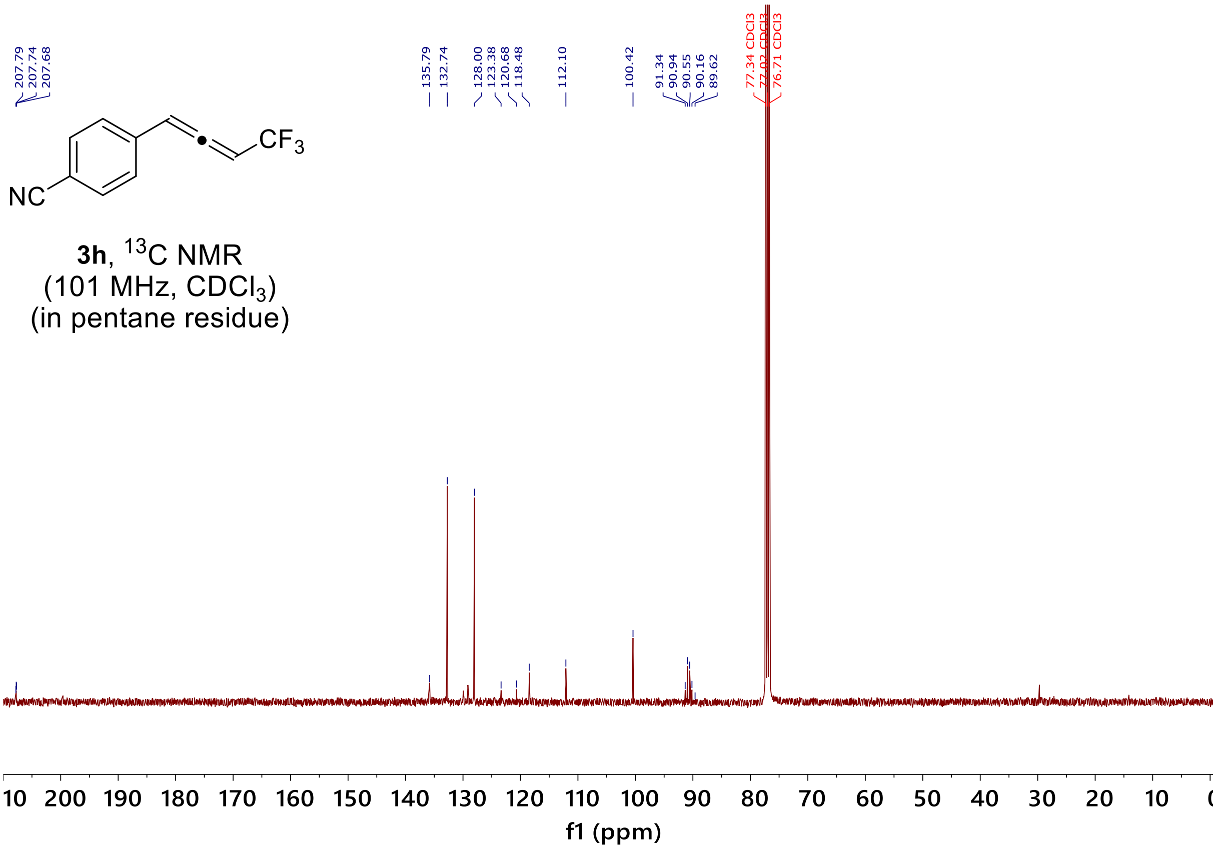

3h, ^{13}C NMR
(101 MHz, $CDCl_3$)
(in pentane residue)

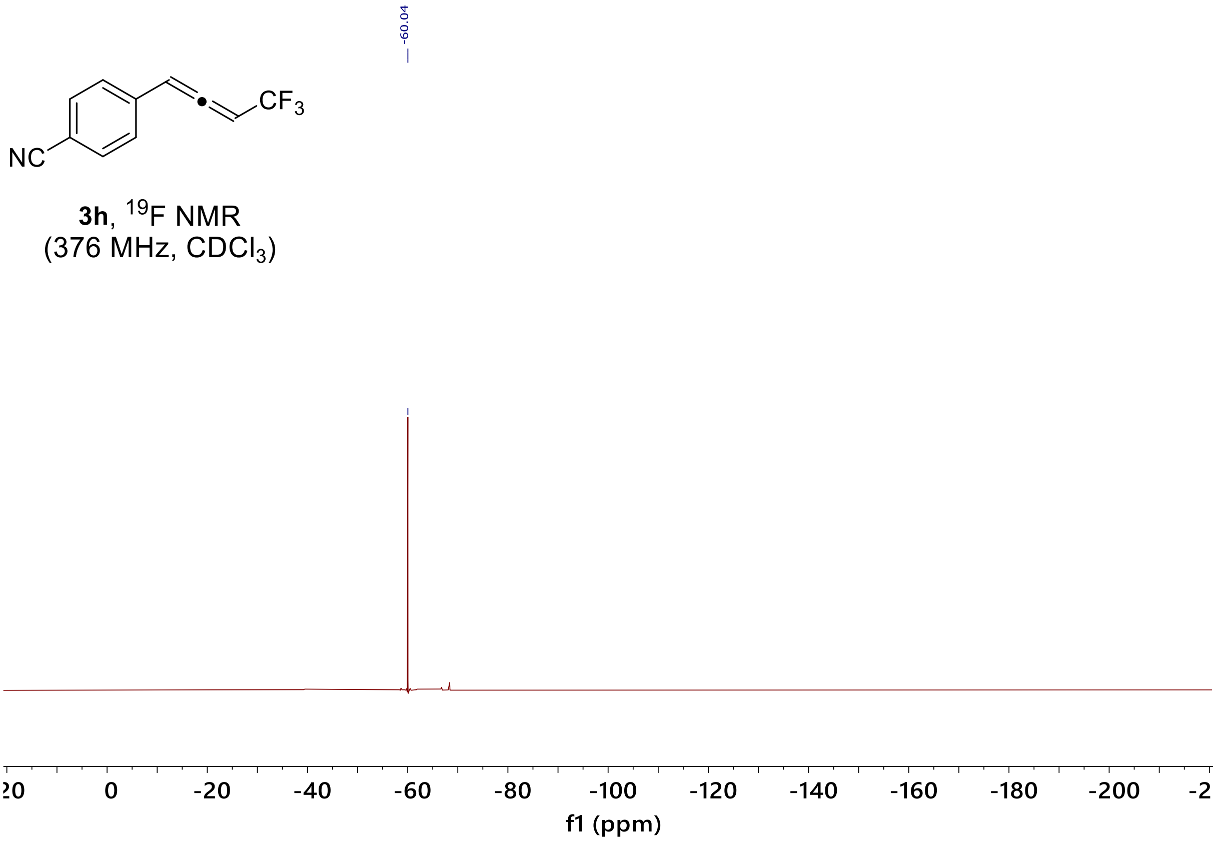

3h, ^{19}F NMR
(376 MHz, $CDCl_3$)

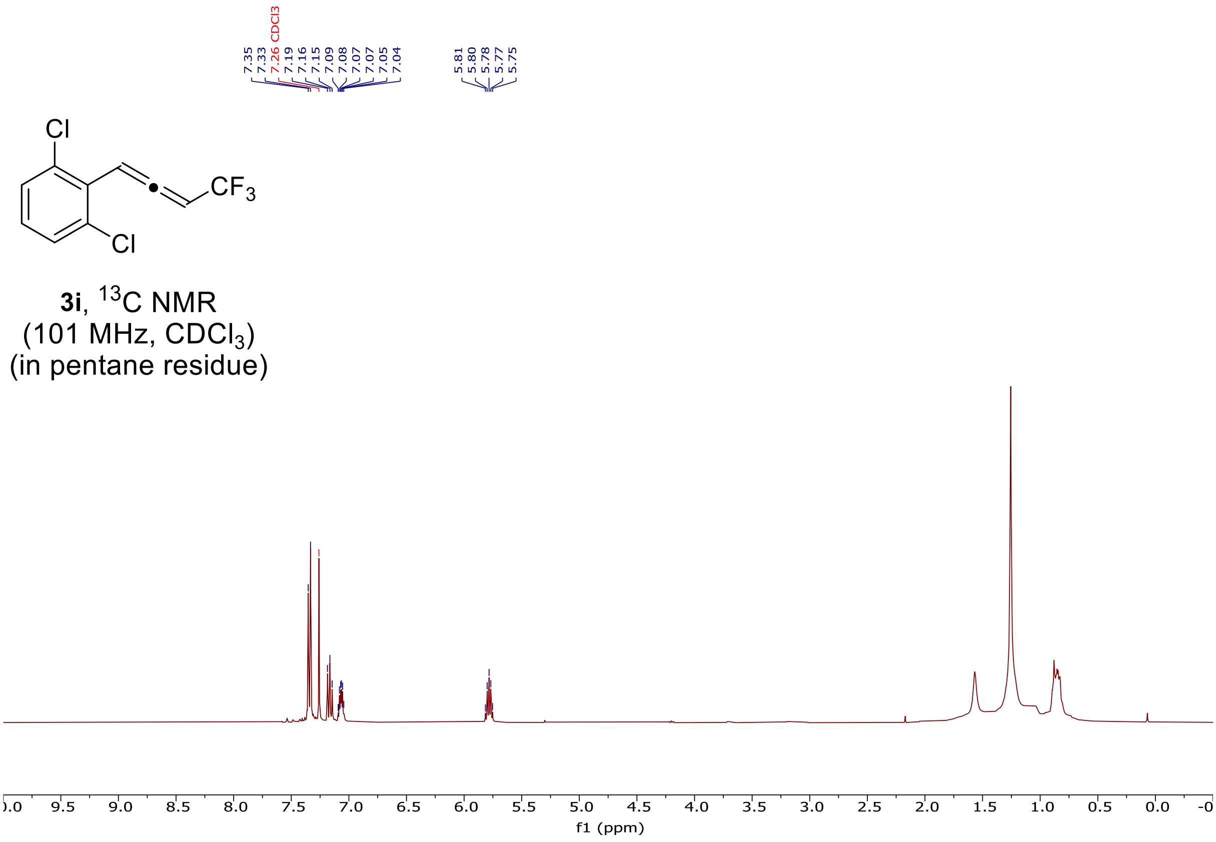

3i, ^{13}C NMR
(101 MHz, $CDCl_3$)
(in pentane residue)

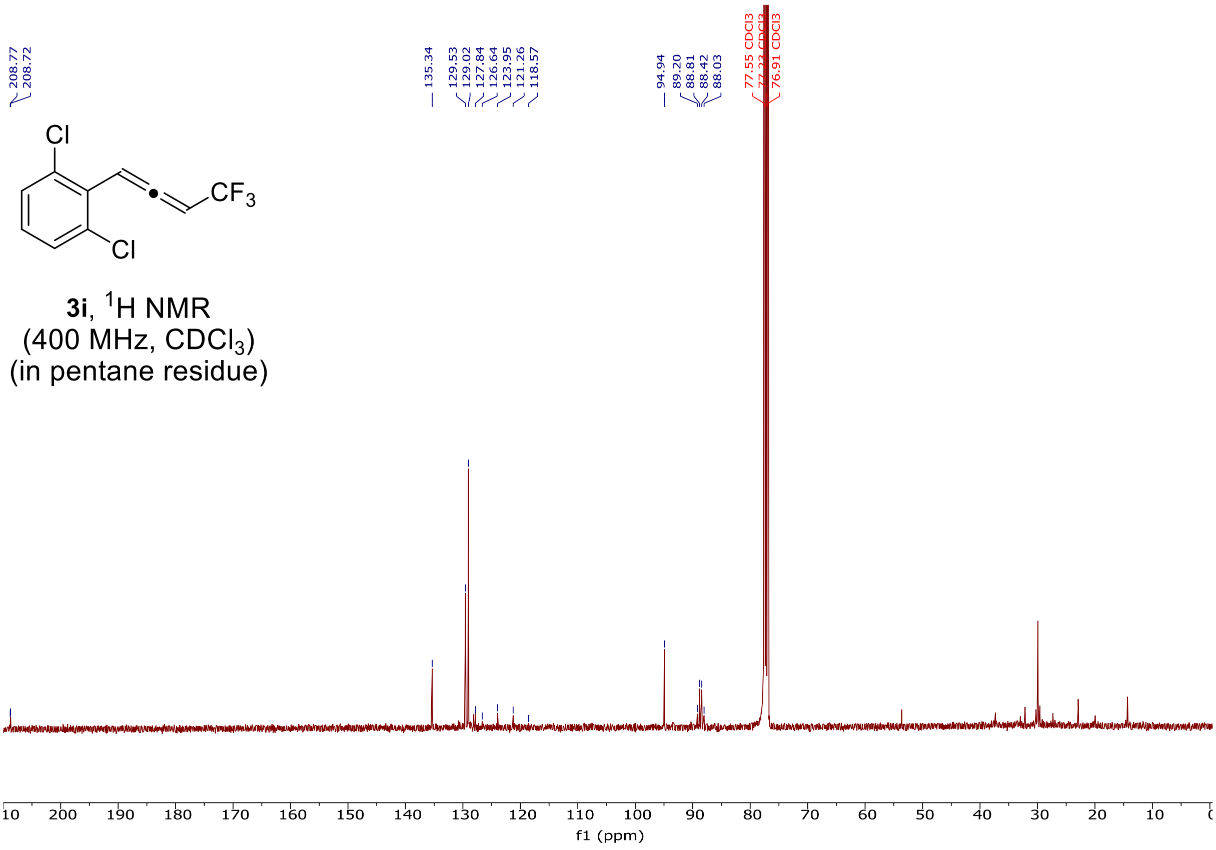

3i, ^{1}H NMR
(400 MHz, $CDCl_3$)
(in pentane residue)

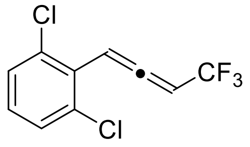

3i, ^{19}F NMR
(376 MHz, $CDCl_3$)

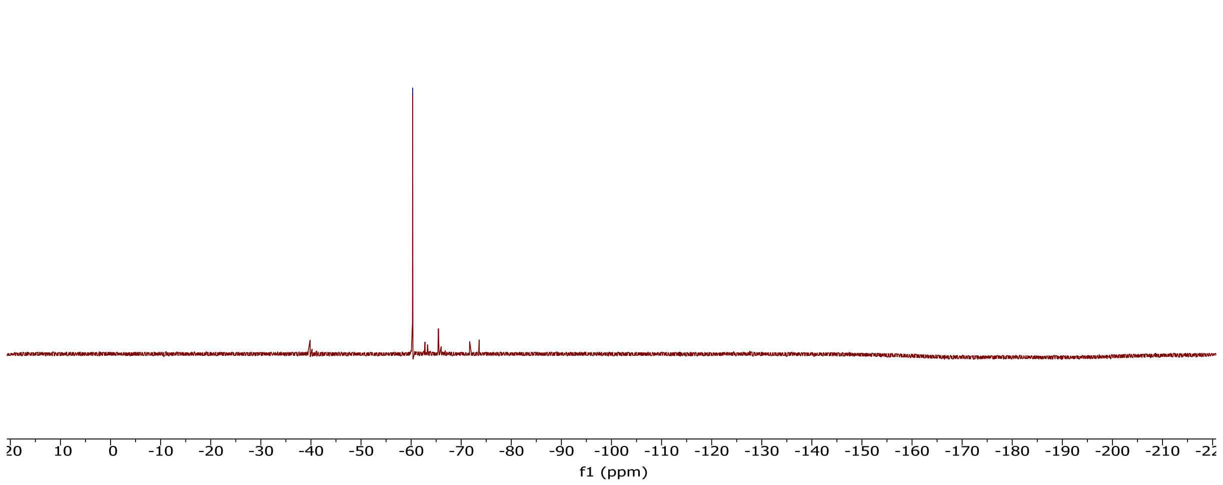

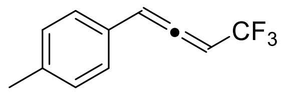

3j, ^{1}H NMR
(400 MHz, $CDCl_3$)
(in pentane residue)

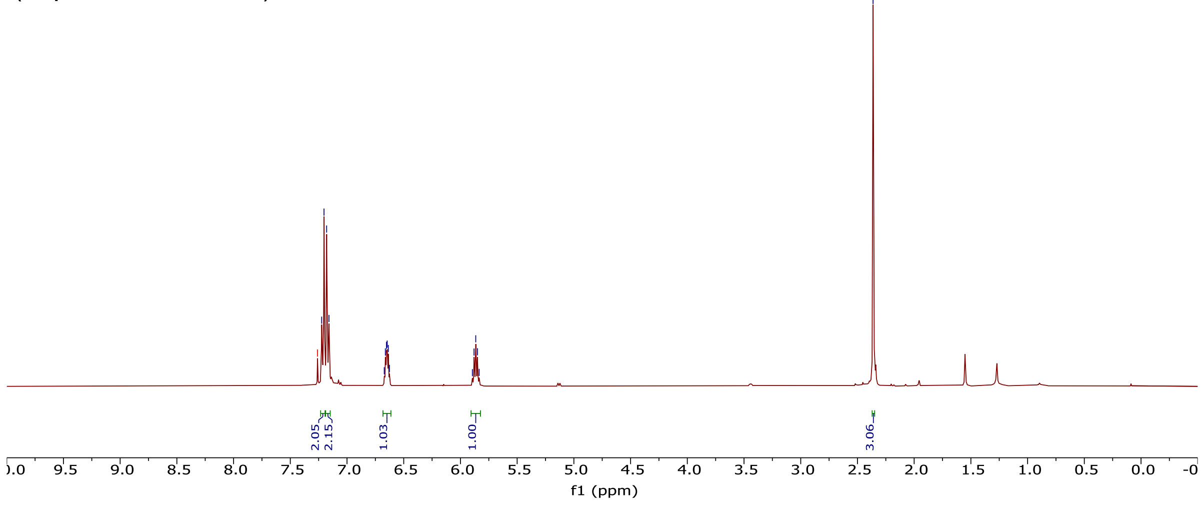

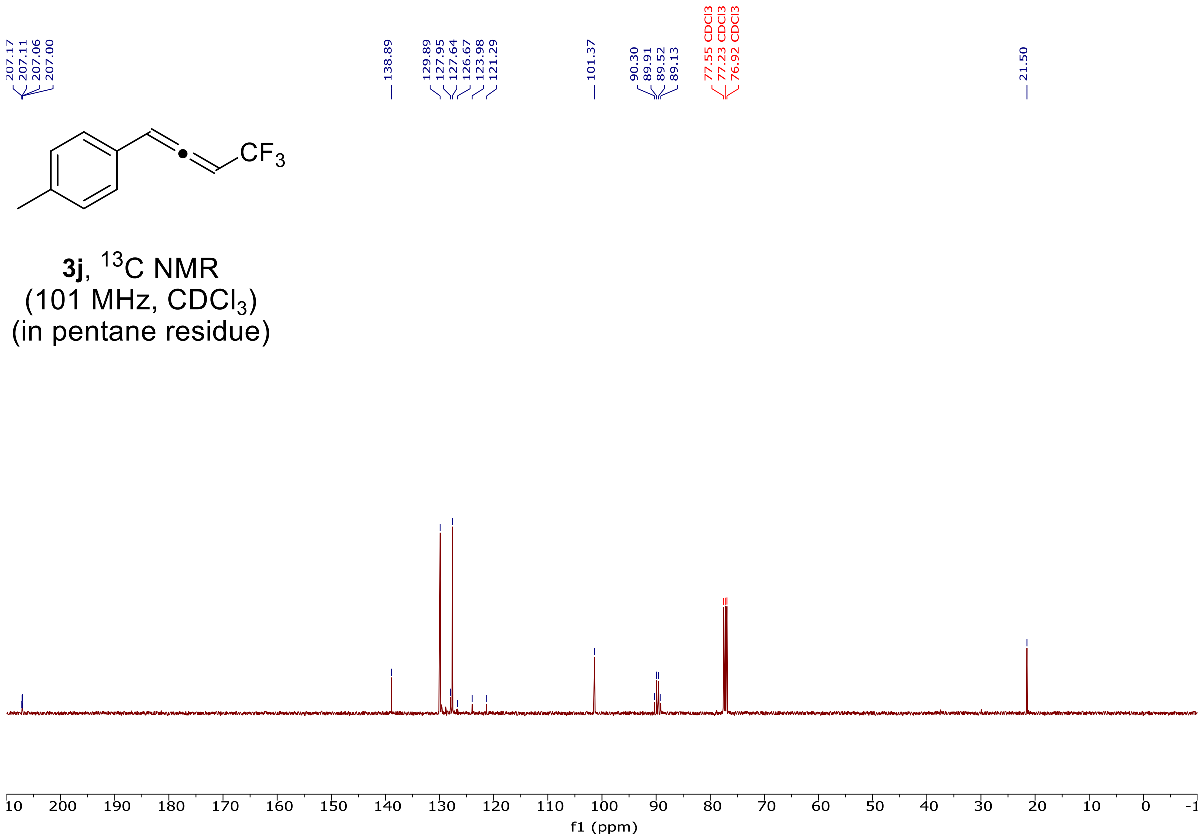
207.17
207.11
207.06
207.00
138.89
129.89
127.95
127.64
126.67
123.98
121.29
101.37
90.30
89.91
89.52
89.13
77.55 CDCl3
77.23 CDCl3
76.92 CDCl3
21.50
CF3
3j, 13C NMR
(101 MHz, CDCl3)
(in pentane residue)
10 200 190 180 170 160 150 140 130 120 110 100 90 80 70 60 50 40 30 20 10 0 -1
f1 (ppm)

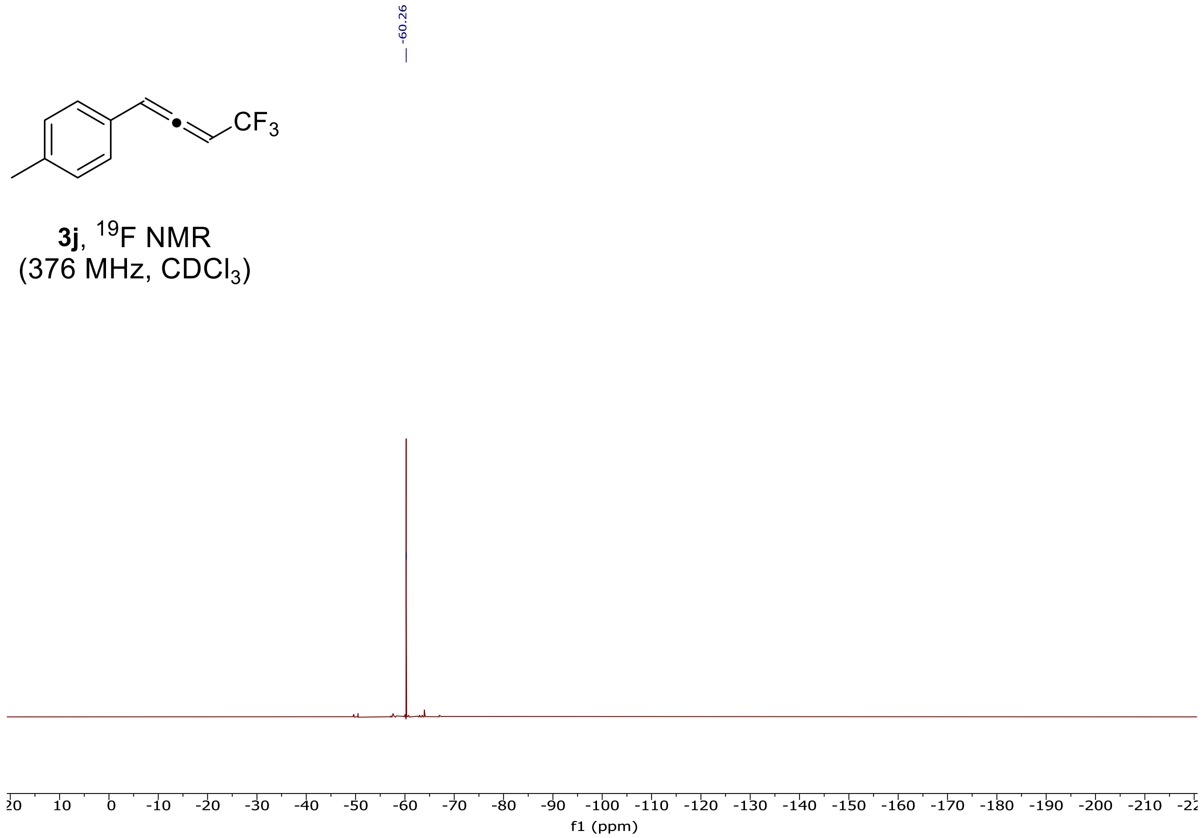
-60.26
CF3
3j, 19F NMR
(376 MHz, CDCl3)
20 10 0 -10 -20 -30 -40 -50 -60 -70 -80 -90 -100 -110 -120 -130 -140 -150 -160 -170 -180 -190 -200 -210 -2
f1 (ppm)

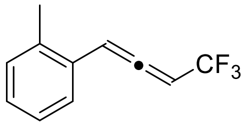

3k, ^{13}C NMR
(101 MHz, $CDCl_3$)
(in pentane residue)

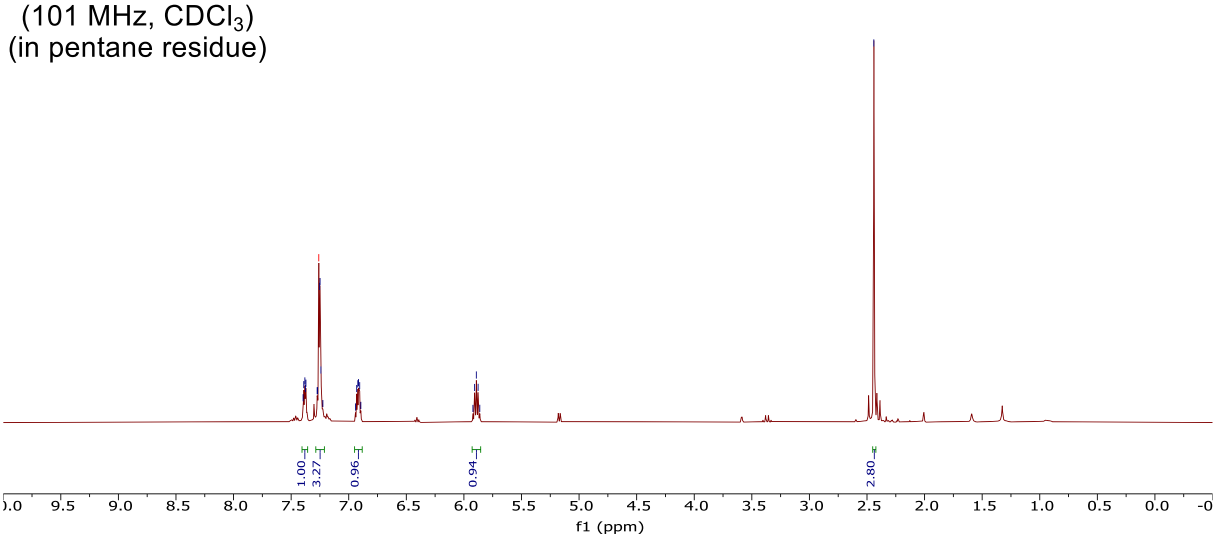

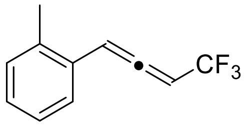

3k, ^{1}H NMR
(400 MHz, $CDCl_3$)
(in pentane residue)

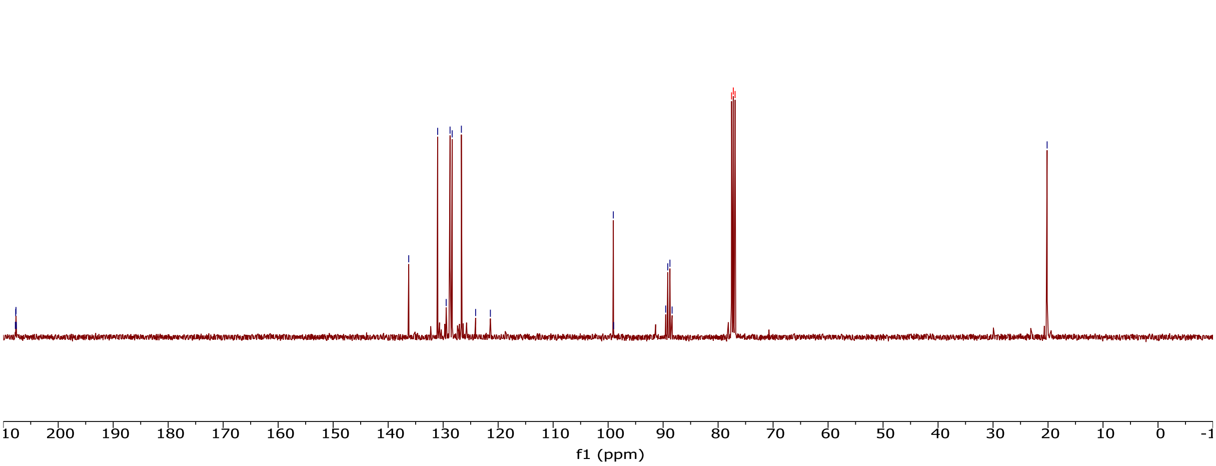

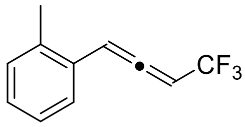

3k, ^{19}F NMR
(376 MHz, $CDCl_3$)

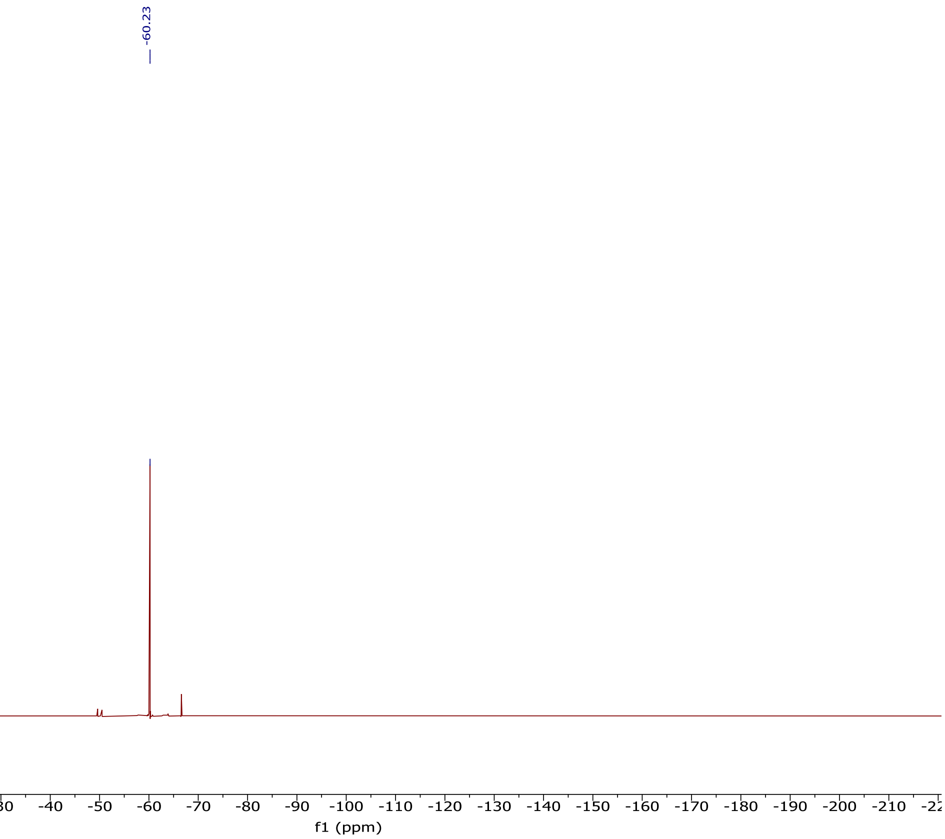

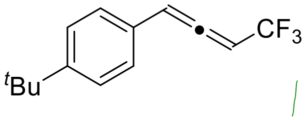

3l, ^{1}H NMR
(400 MHz, $CDCl_3$)
(in pentane residue)

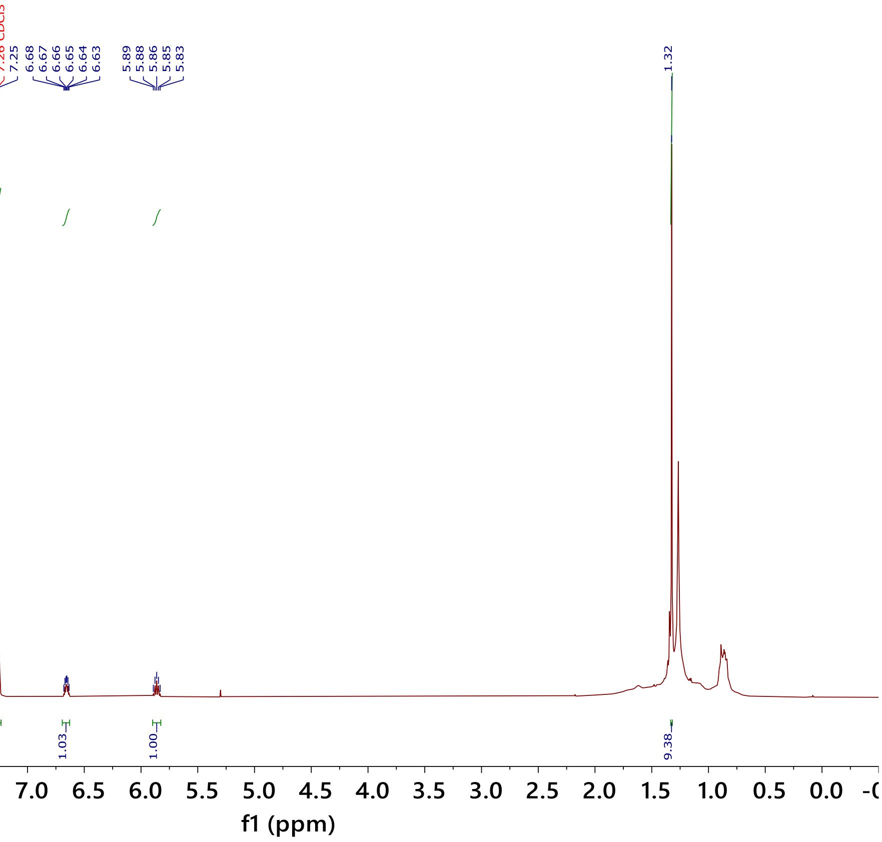

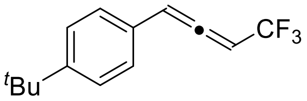

3l, ¹³C NMR
(101 MHz, $CDCl_3$)
(in pentane residue)

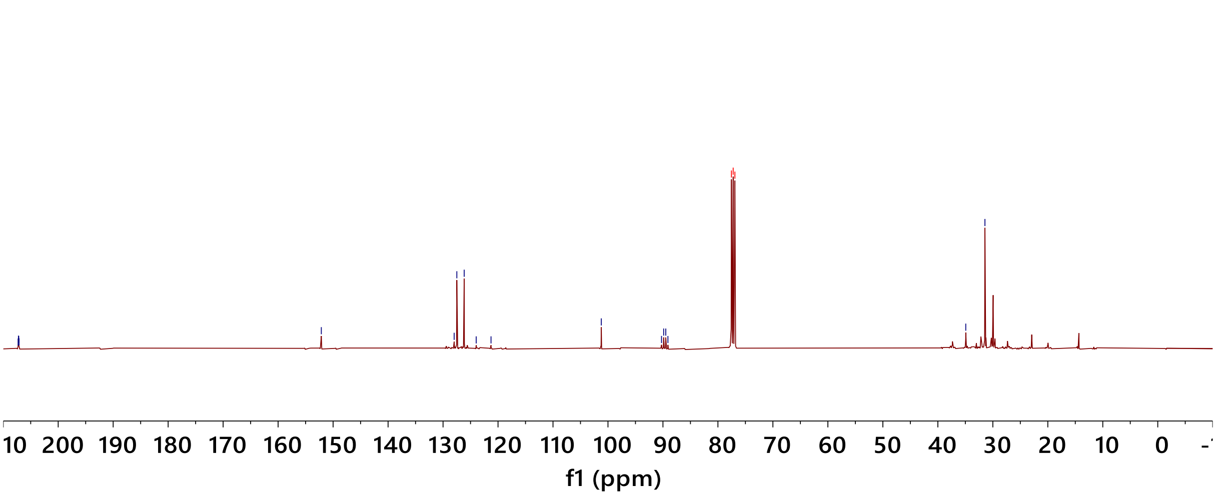

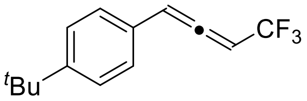

3l, ¹⁹F NMR
(376 MHz, $CDCl_3$)

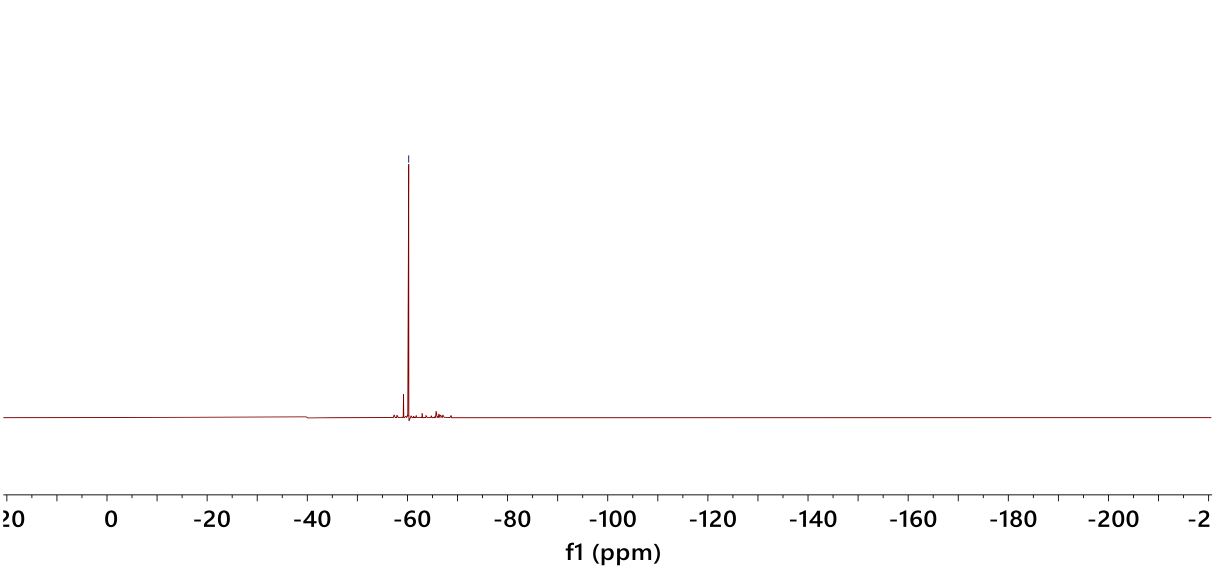

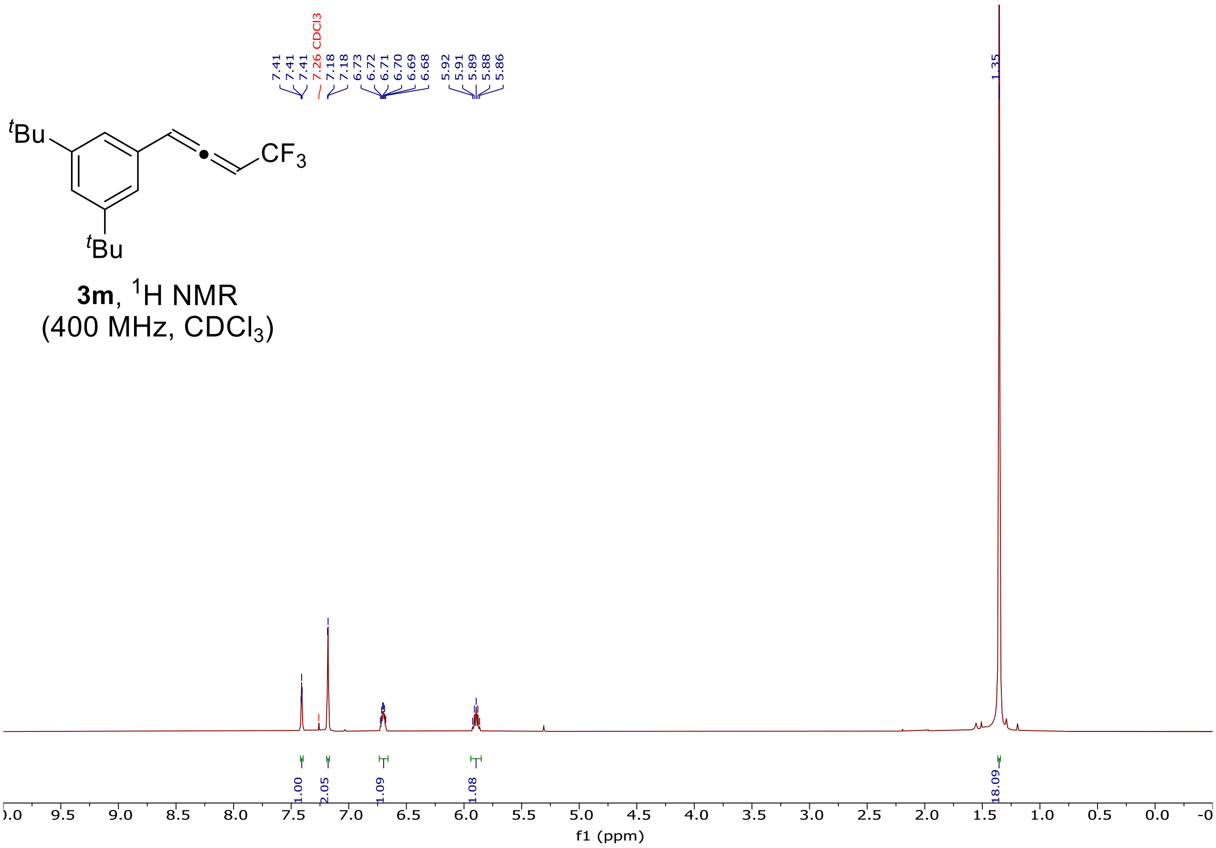

3m, ^{1}H NMR
(400 MHz, $CDCl_3$)

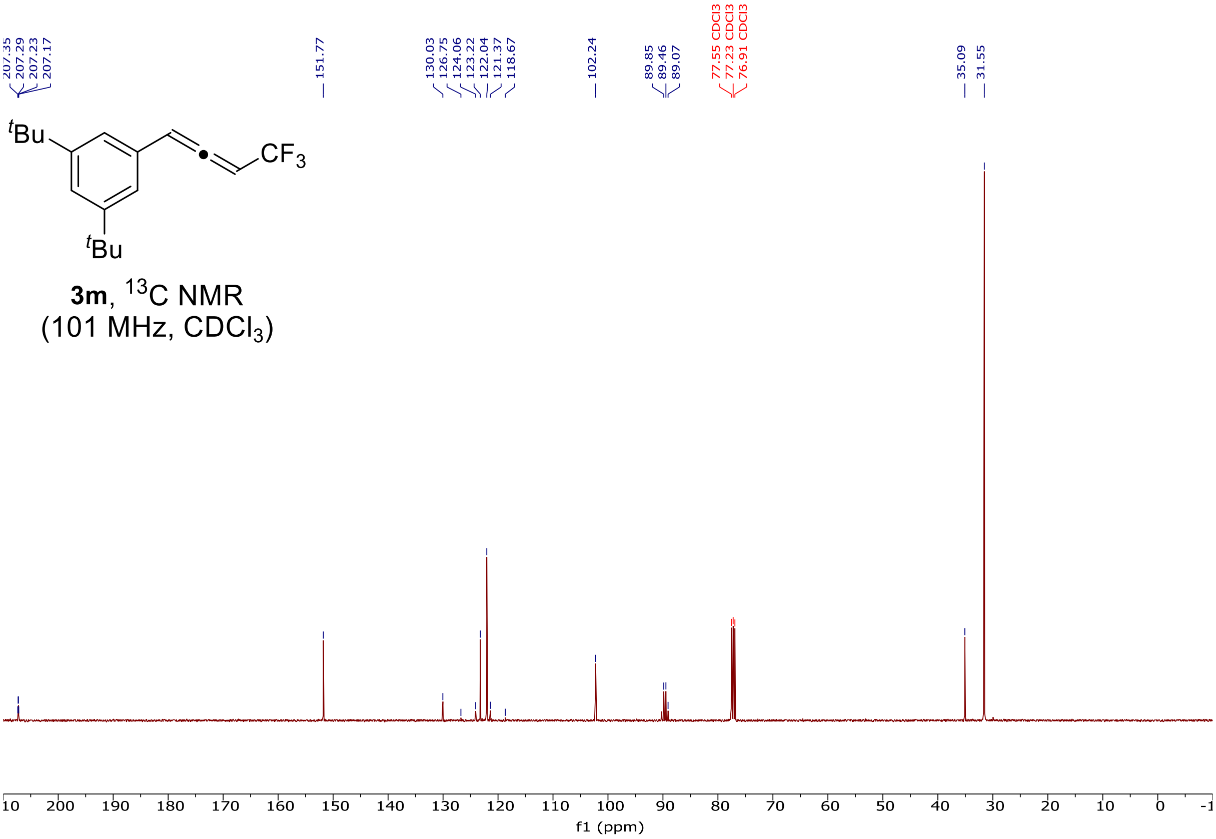

3m, ^{13}C NMR
(101 MHz, $CDCl_3$)

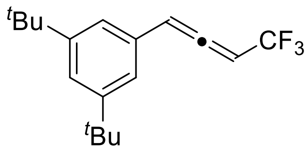

3m, ^{19}F NMR
(376 MHz, $CDCl_3$)

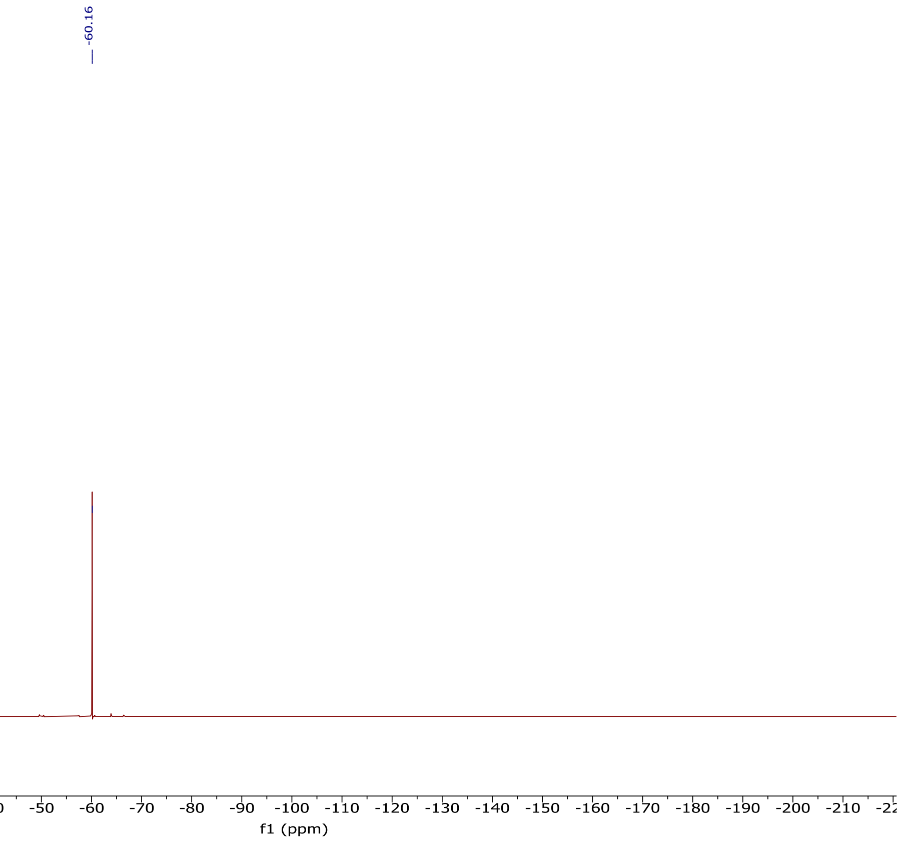

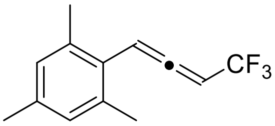

3n, ^{1}H NMR
(400 MHz, $CDCl_3$)
(in pentane residue)

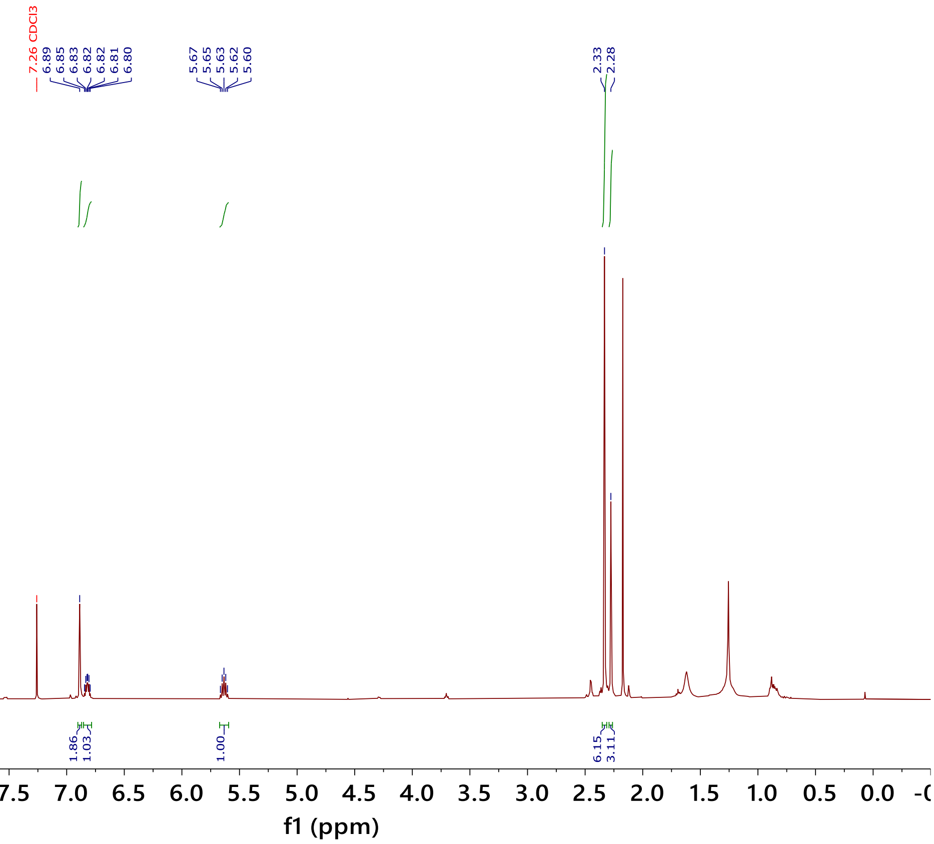

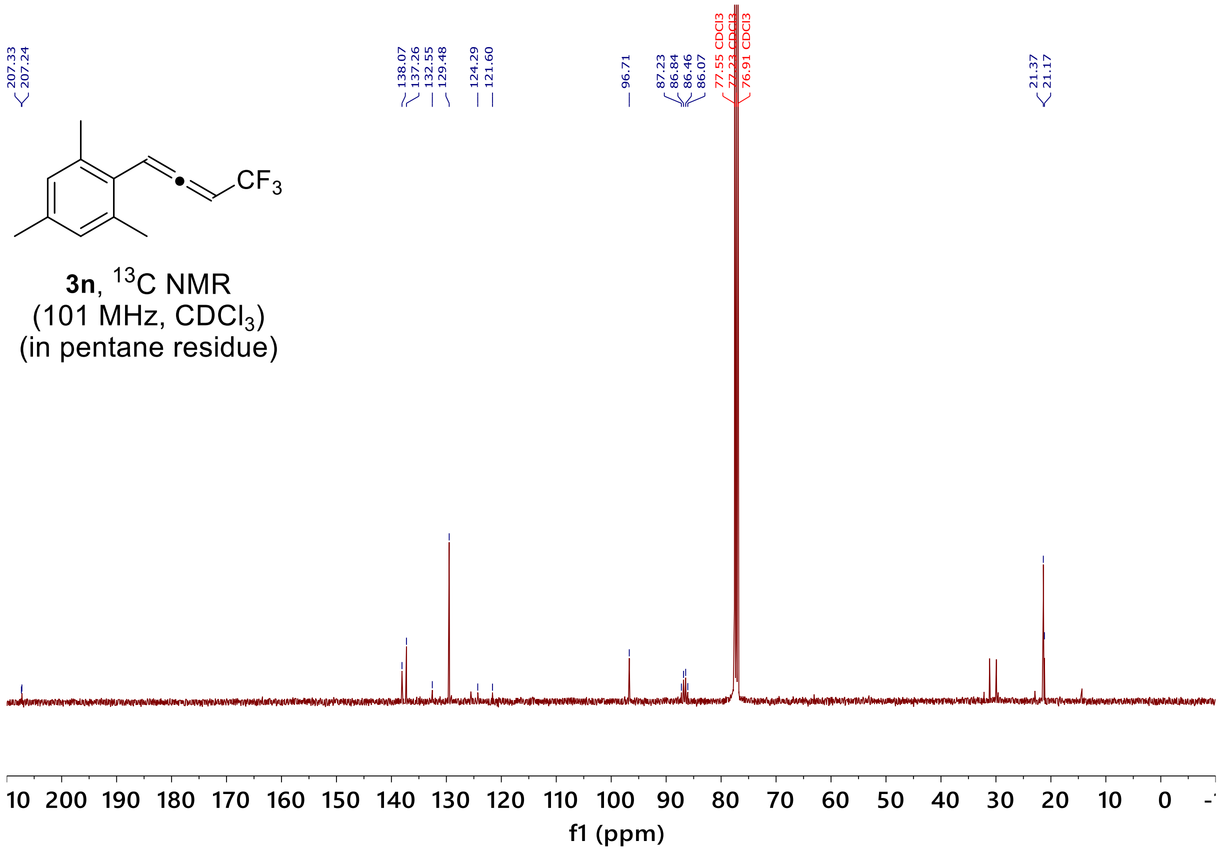
207.33
207.24
138.07
137.26
132.55
129.48
124.29
121.60
96.71
87.23
86.84
86.46
86.07
77.55 CDCl3
77.23 CDCl3
76.91 CDCl3
21.37
21.17
CF3
3n, 13C NMR
(101 MHz, CDCl3)
(in pentane residue)
f1 (ppm)

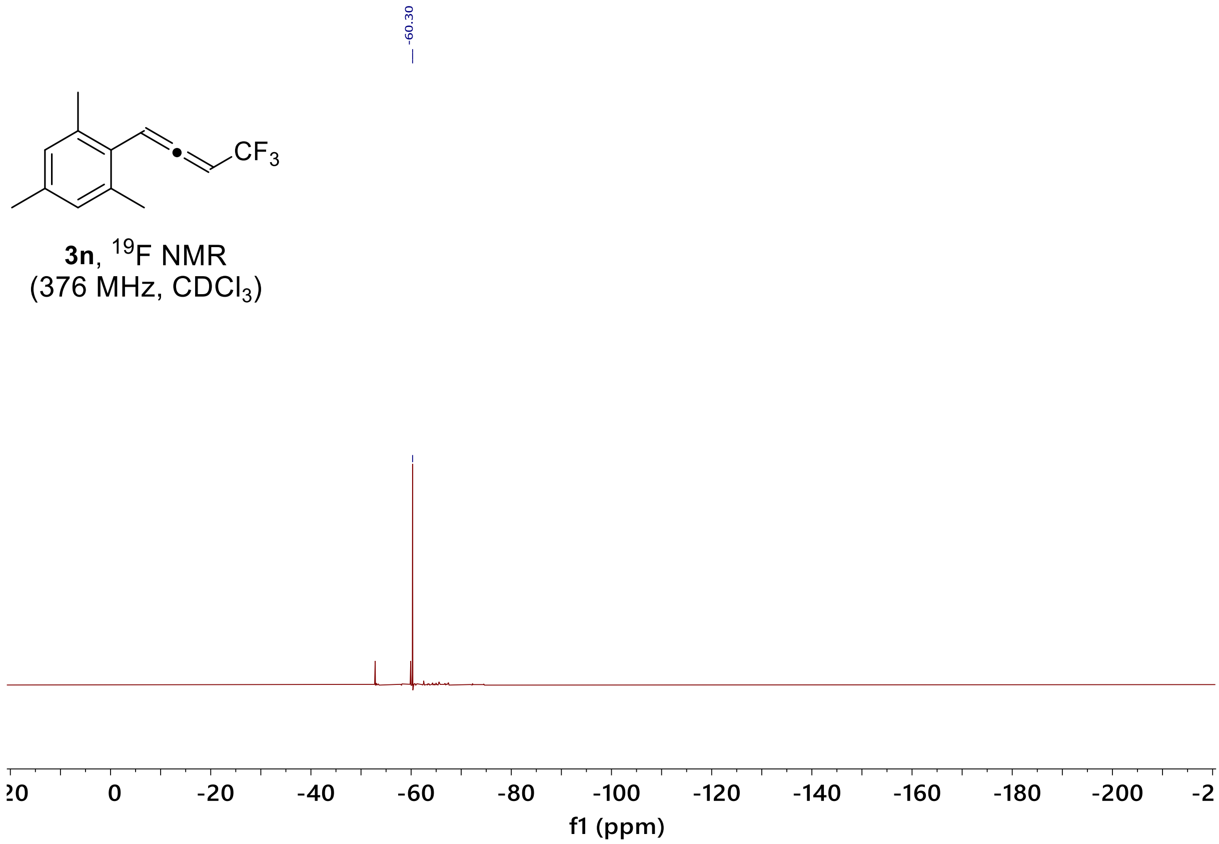
-60.30
CF3
3n, 19F NMR
(376 MHz, CDCl3)
f1 (ppm)

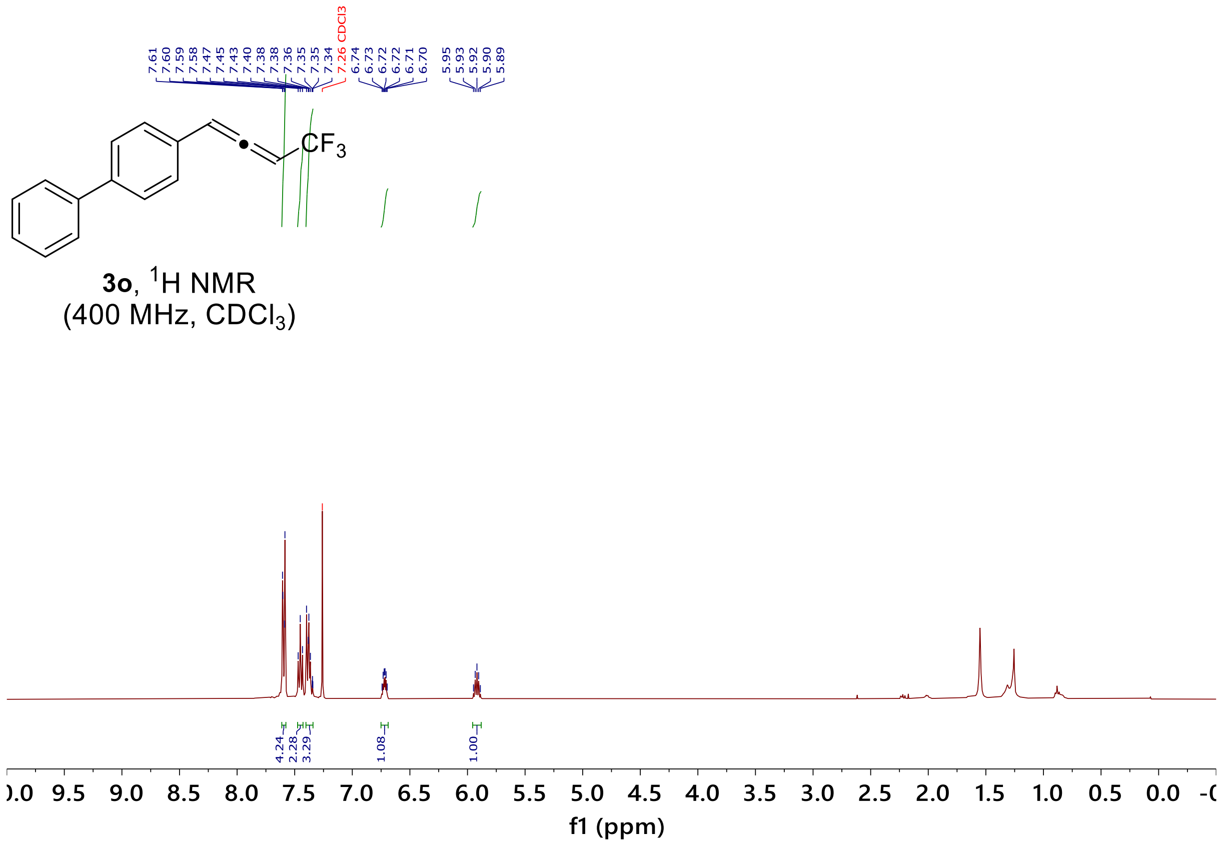

3o, ^{1}H NMR
(400 MHz, $CDCl_3$)

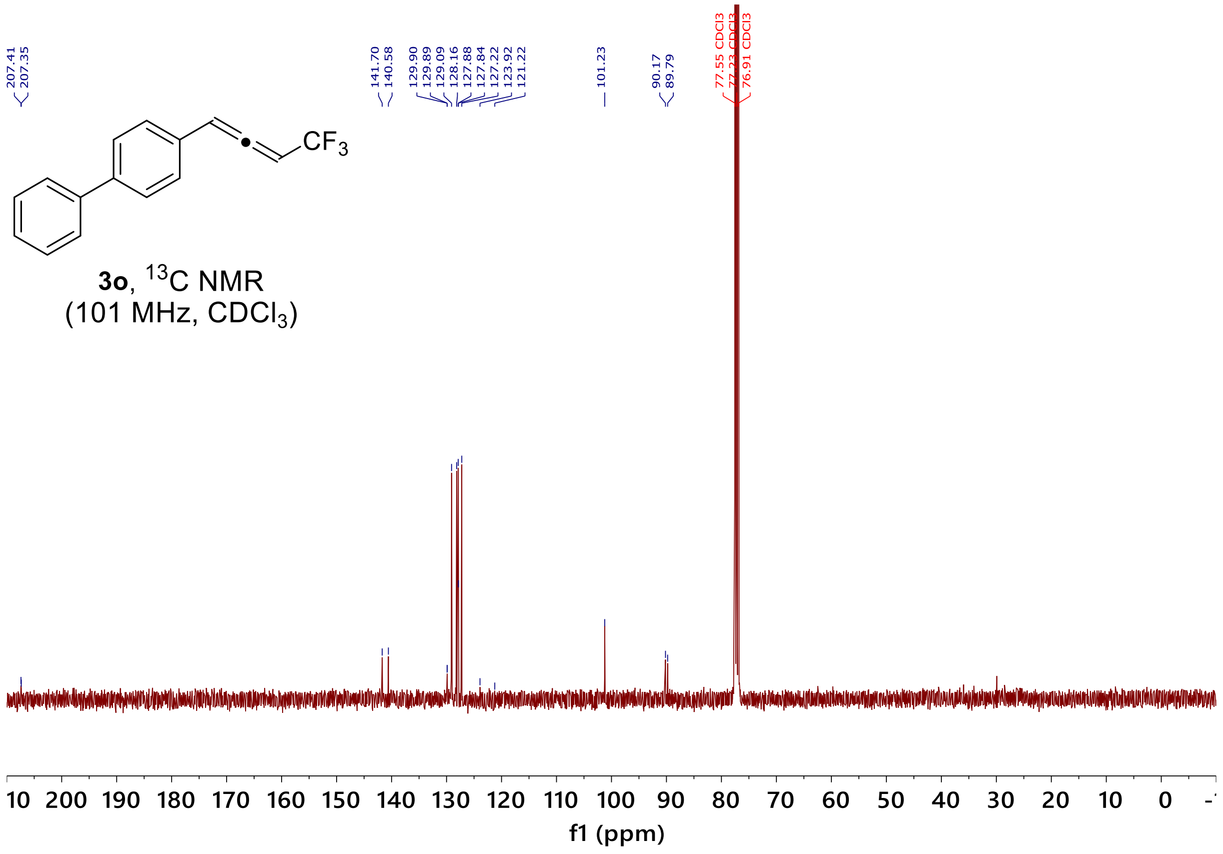

3o, ^{13}C NMR
(101 MHz, $CDCl_3$)

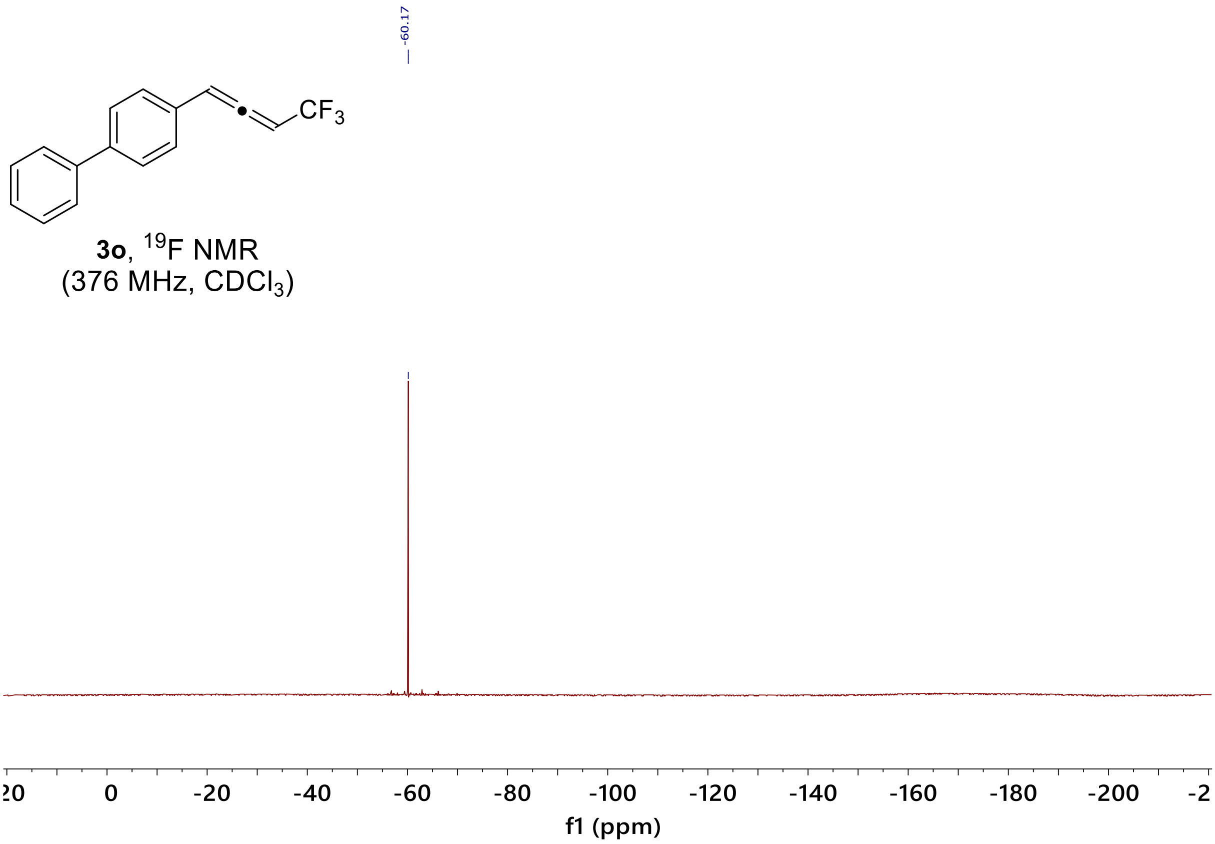

3o, ^{19}F NMR
(376 MHz, $CDCl_3$)

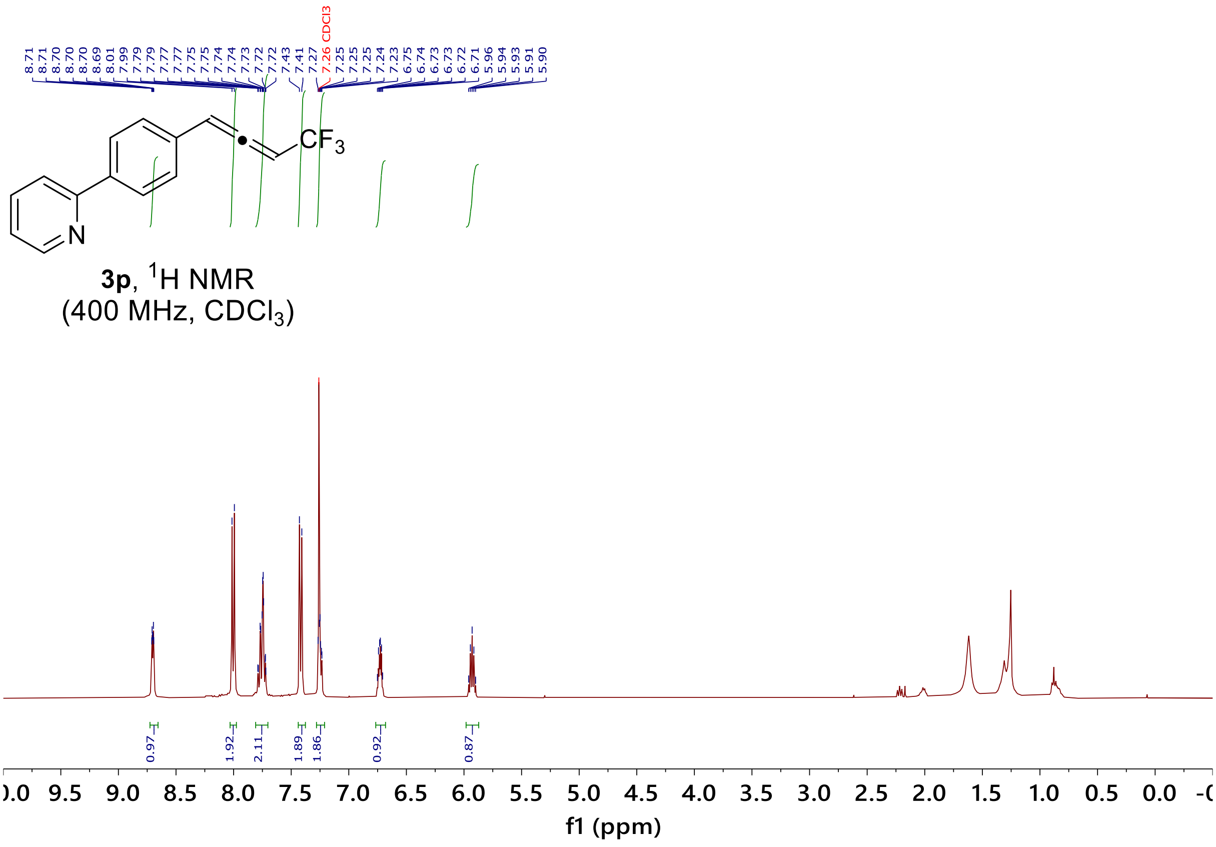

3p, ^{1}H NMR
(400 MHz, $CDCl_3$)

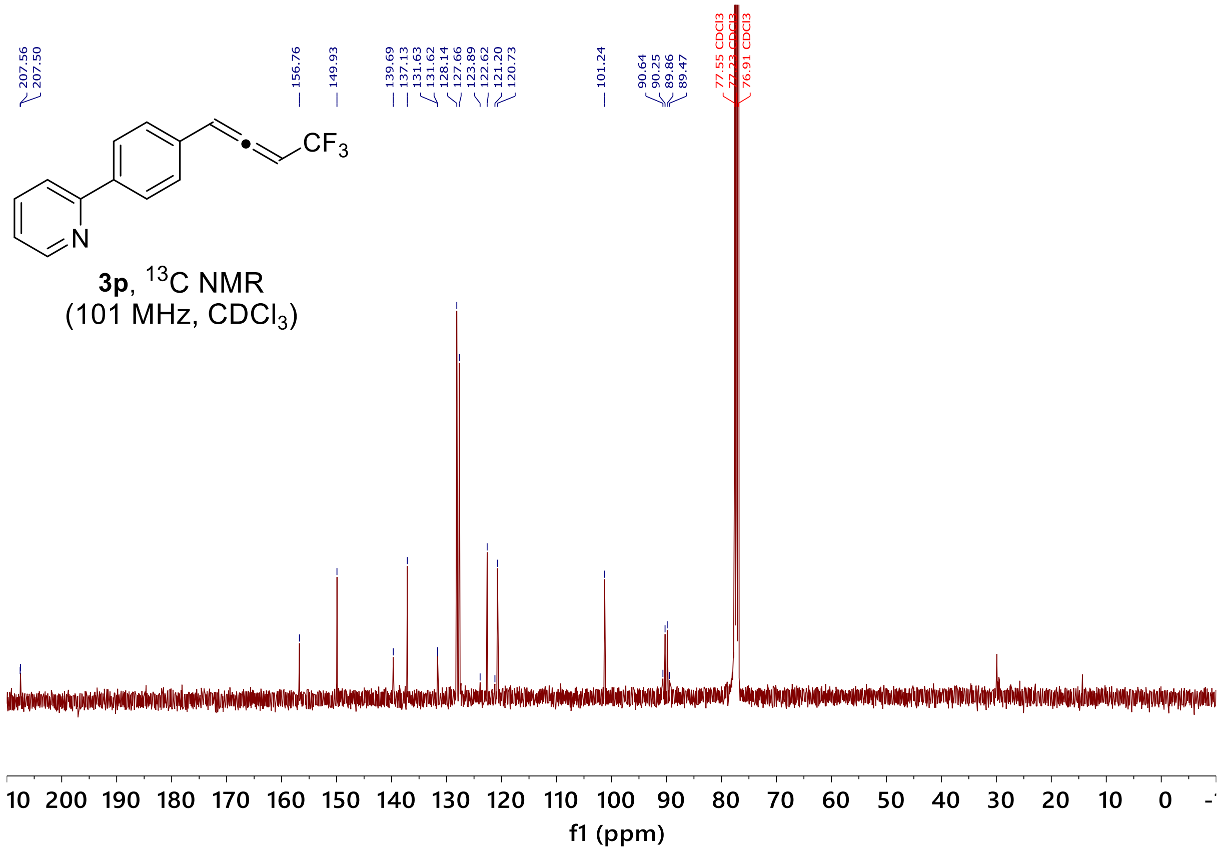

207.56
207.50
156.76
149.93
139.69
137.13
131.63
131.62
128.14
127.66
123.89
122.62
121.20
120.73
101.24
90.64
90.25
89.86
89.47
77.55 CDCl3
77.23 CDCl3
76.91 CDCl3
CF3
N
3p, 13C NMR
(101 MHz, CDCl3)
f1 (ppm)

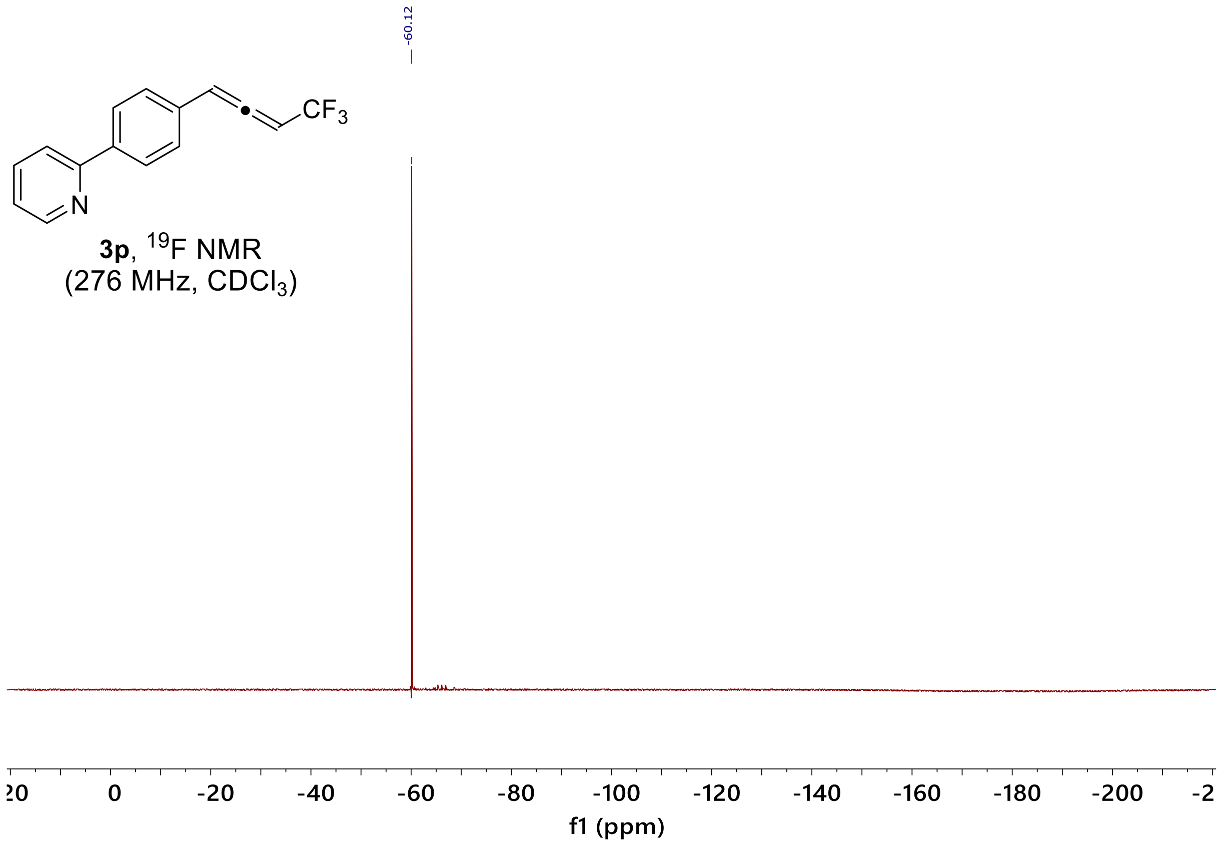

-60.12
CF3
N
3p, 19F NMR
(276 MHz, CDCl3)
f1 (ppm)

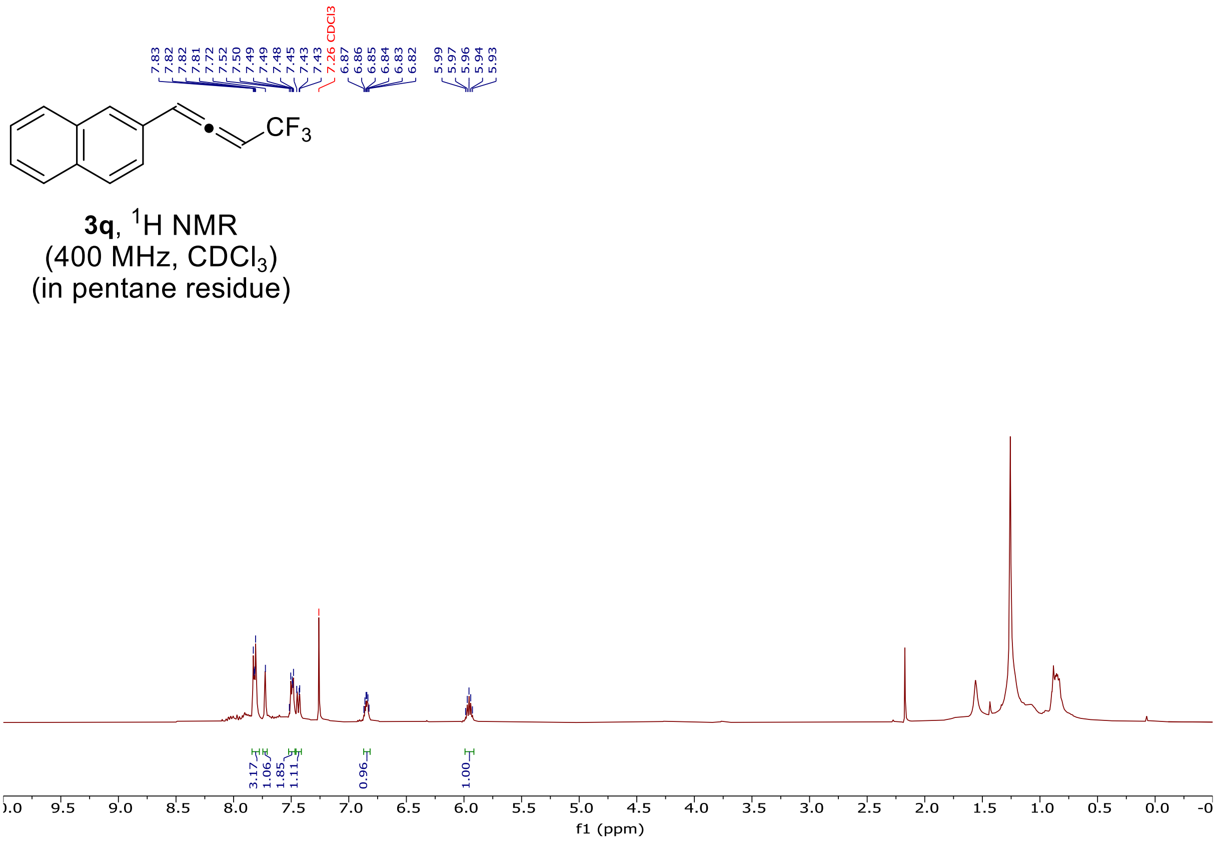

3q, ^{1}H NMR
(400 MHz, $CDCl_3$)
(in pentane residue)

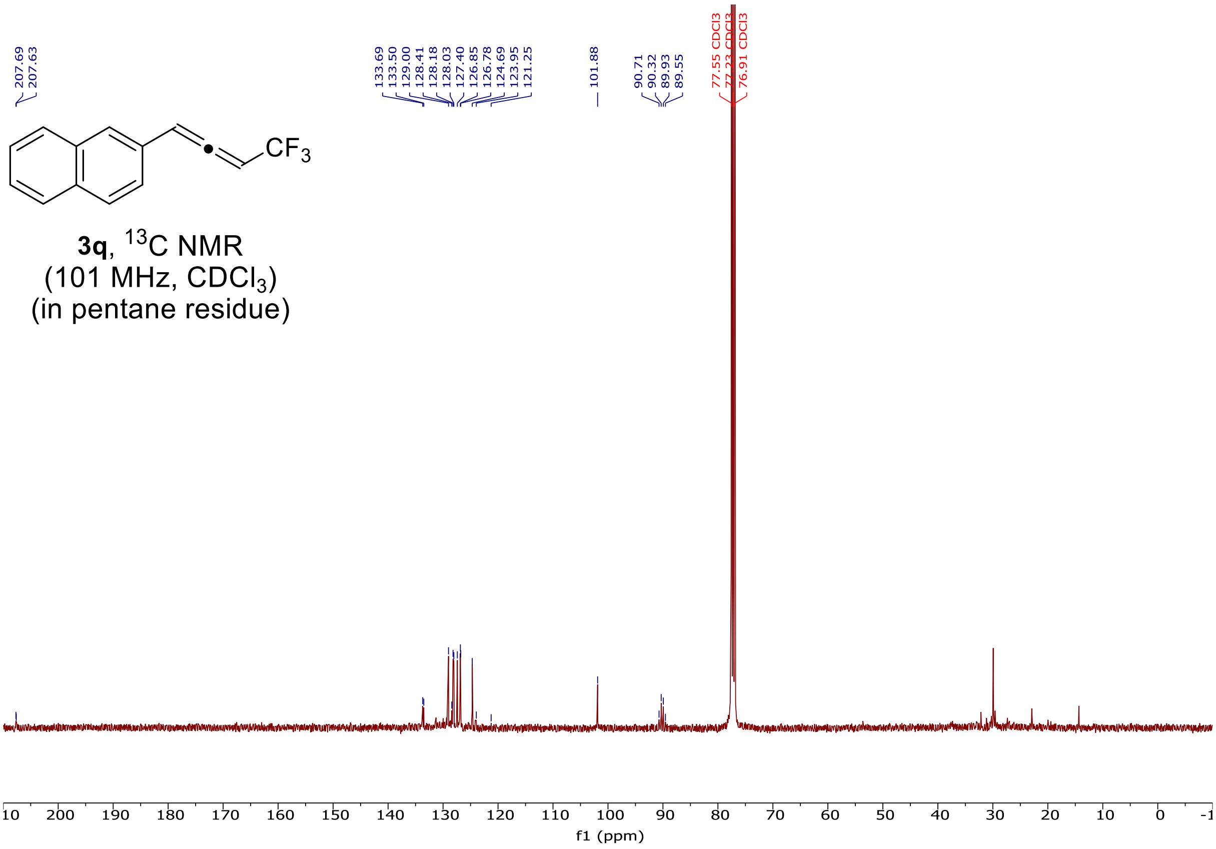

3q, ^{13}C NMR
(101 MHz, $CDCl_3$)
(in pentane residue)

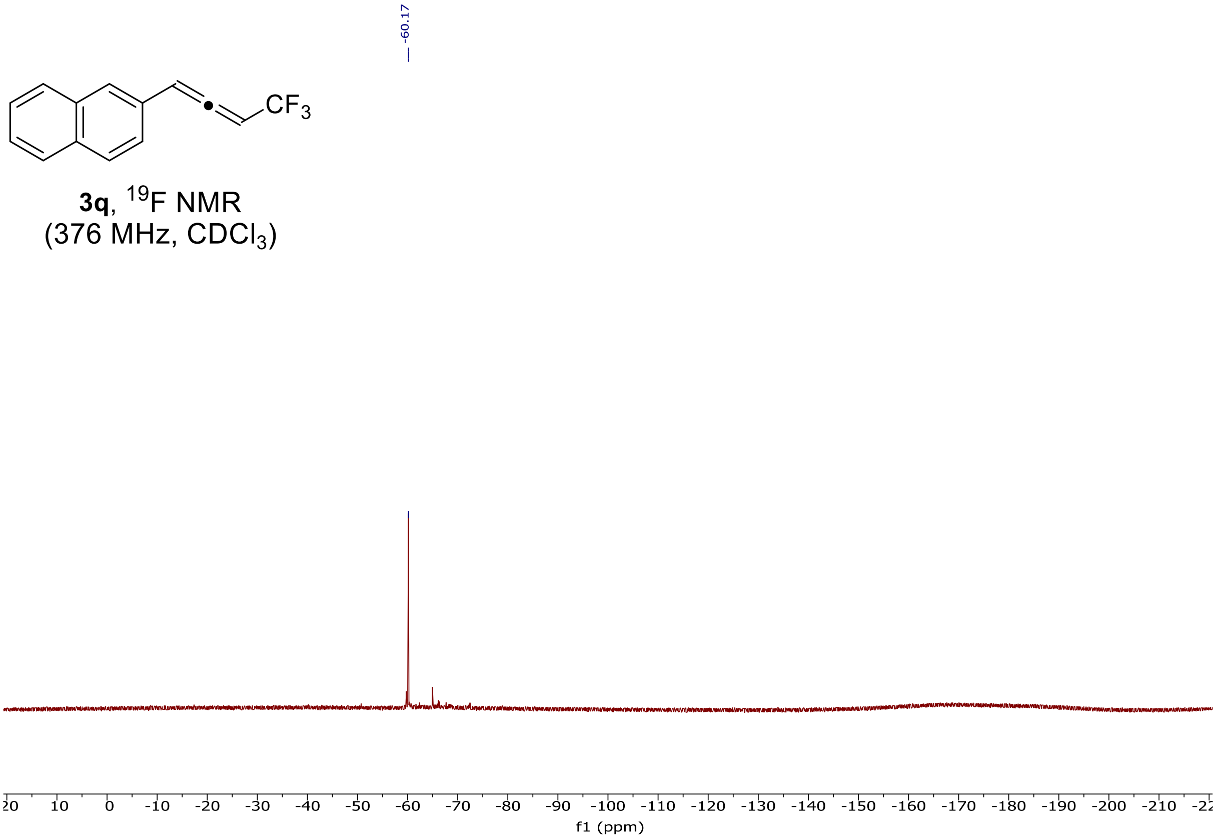

3q, ^{19}F NMR
(376 MHz, $CDCl_3$)

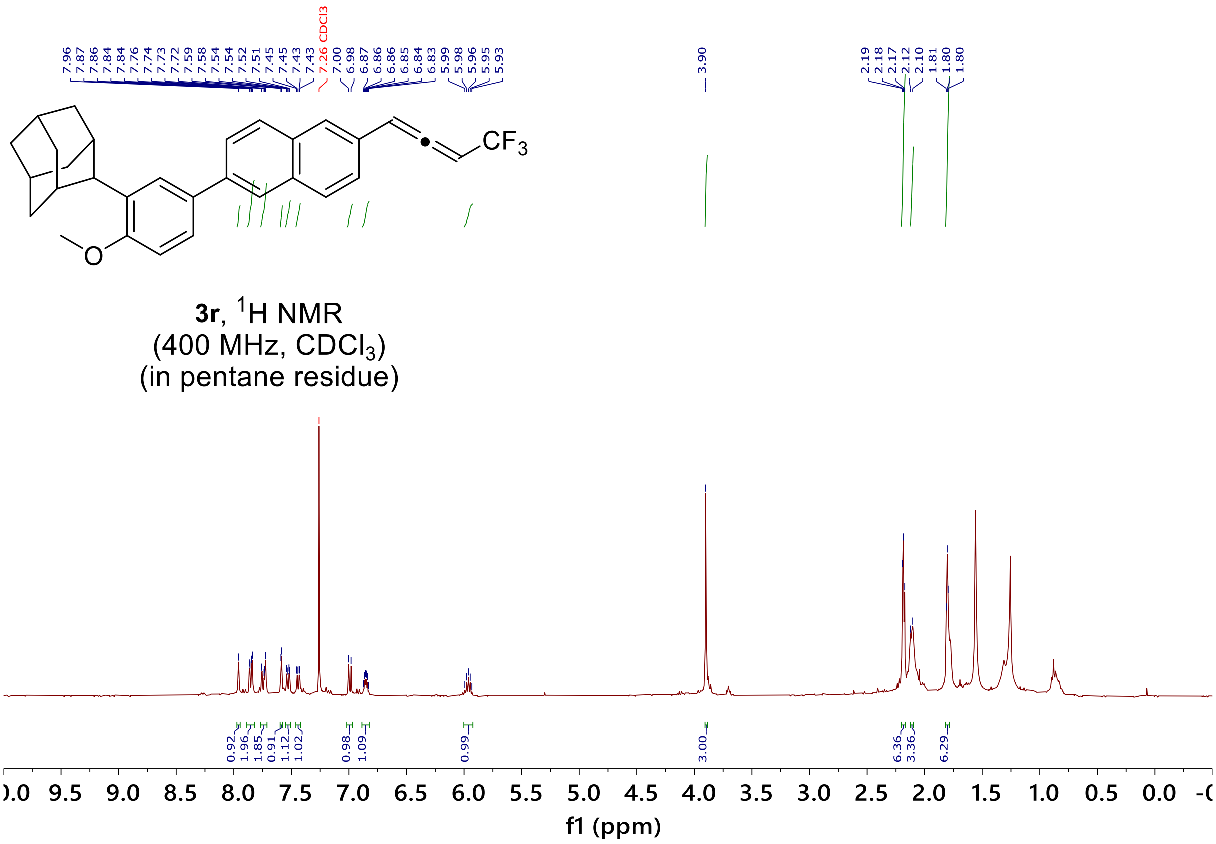

3r, ^{1}H NMR
(400 MHz, $CDCl_3$)
(in pentane residue)

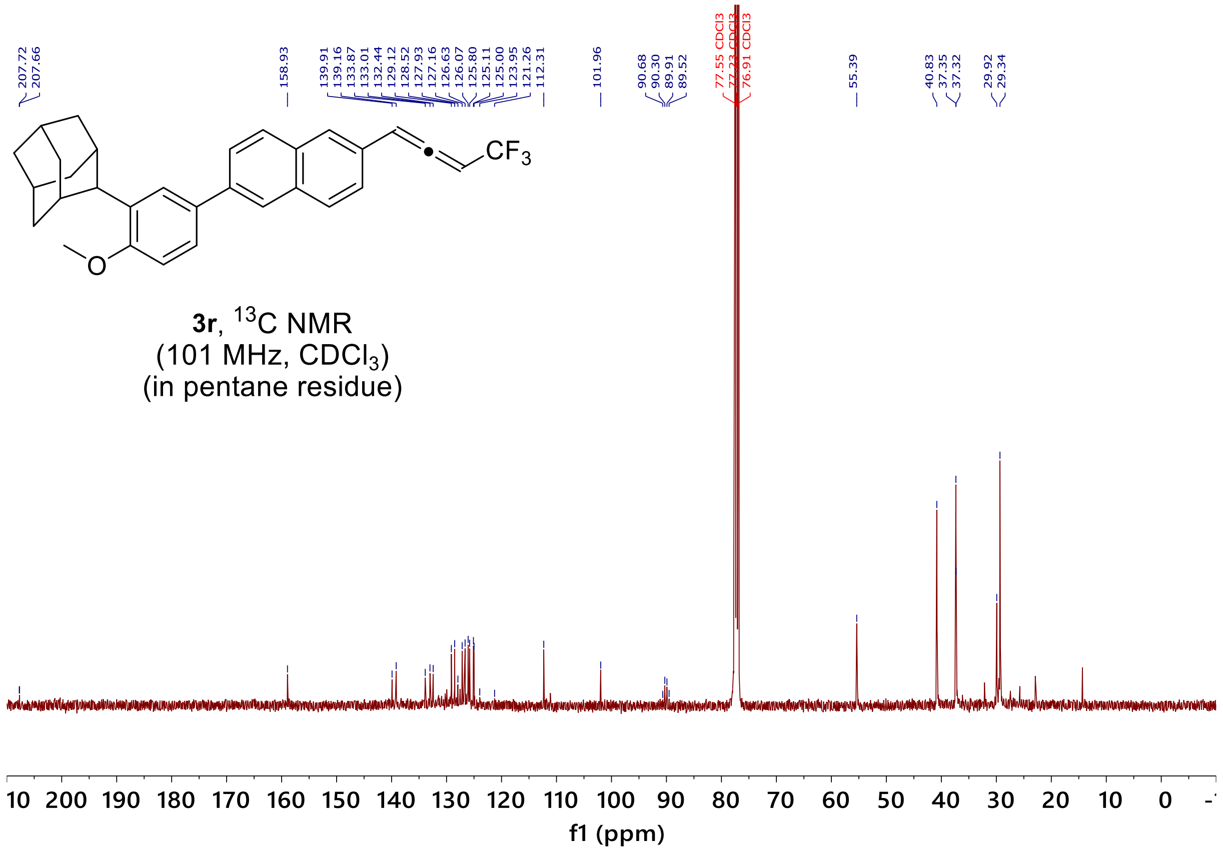

3r, ^{13}C NMR
(101 MHz, $CDCl_3$)
(in pentane residue)

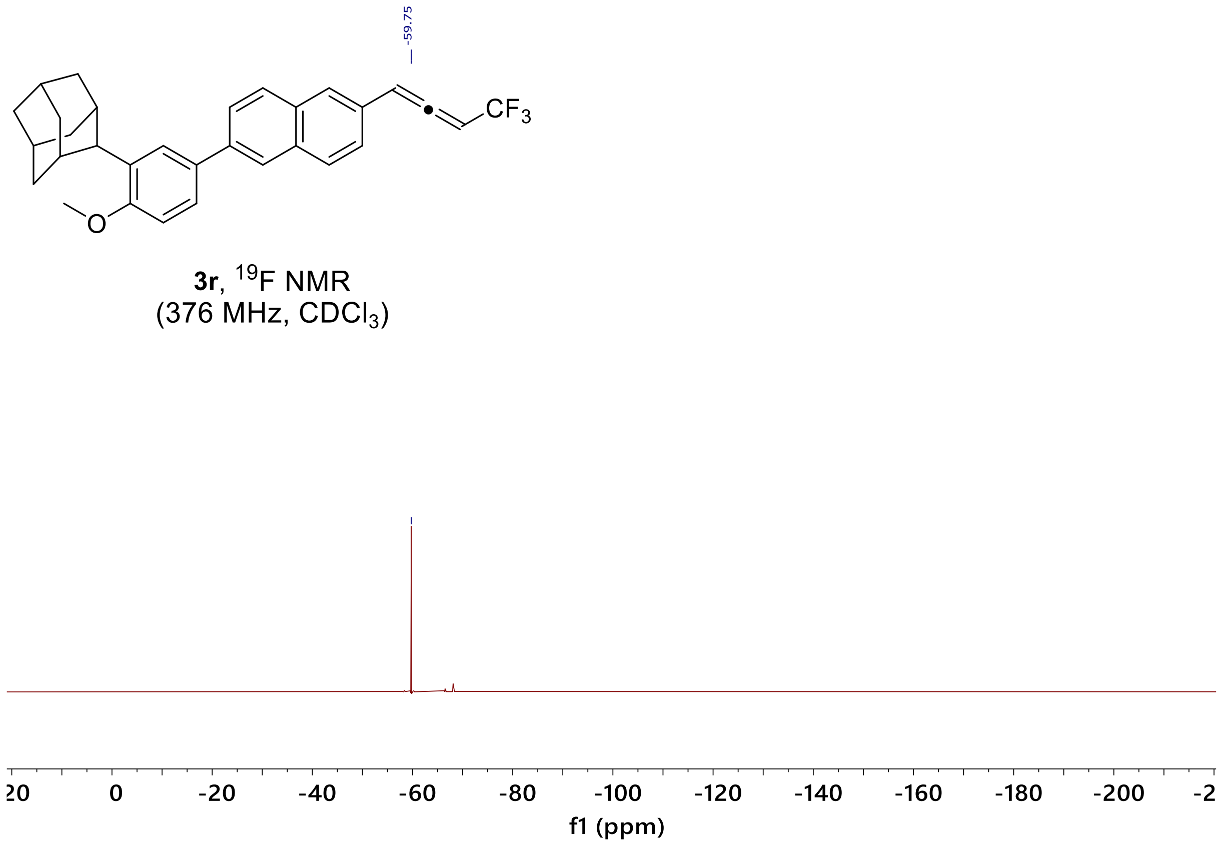

3r, ^{19}F NMR
(376 MHz, $CDCl_3$)

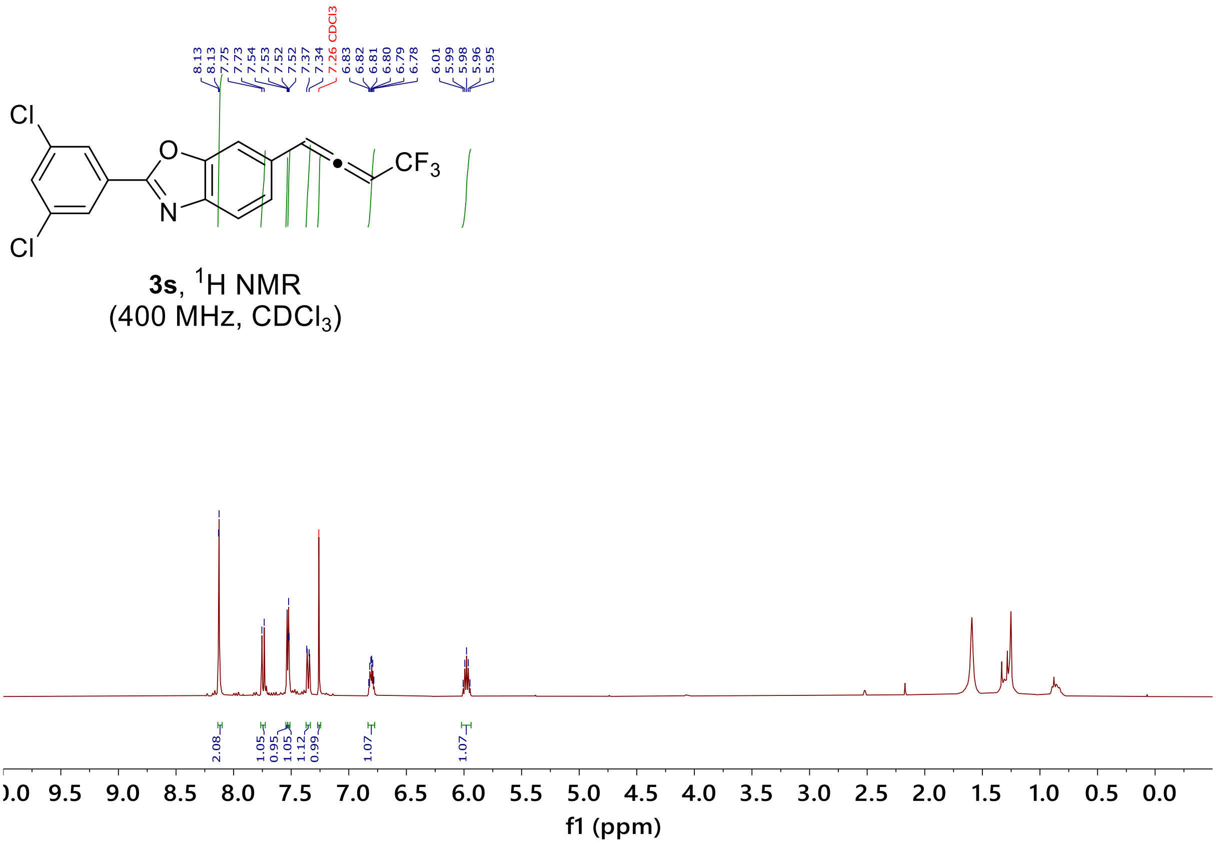

3s, ^{1}H NMR
(400 MHz, $CDCl_3$)

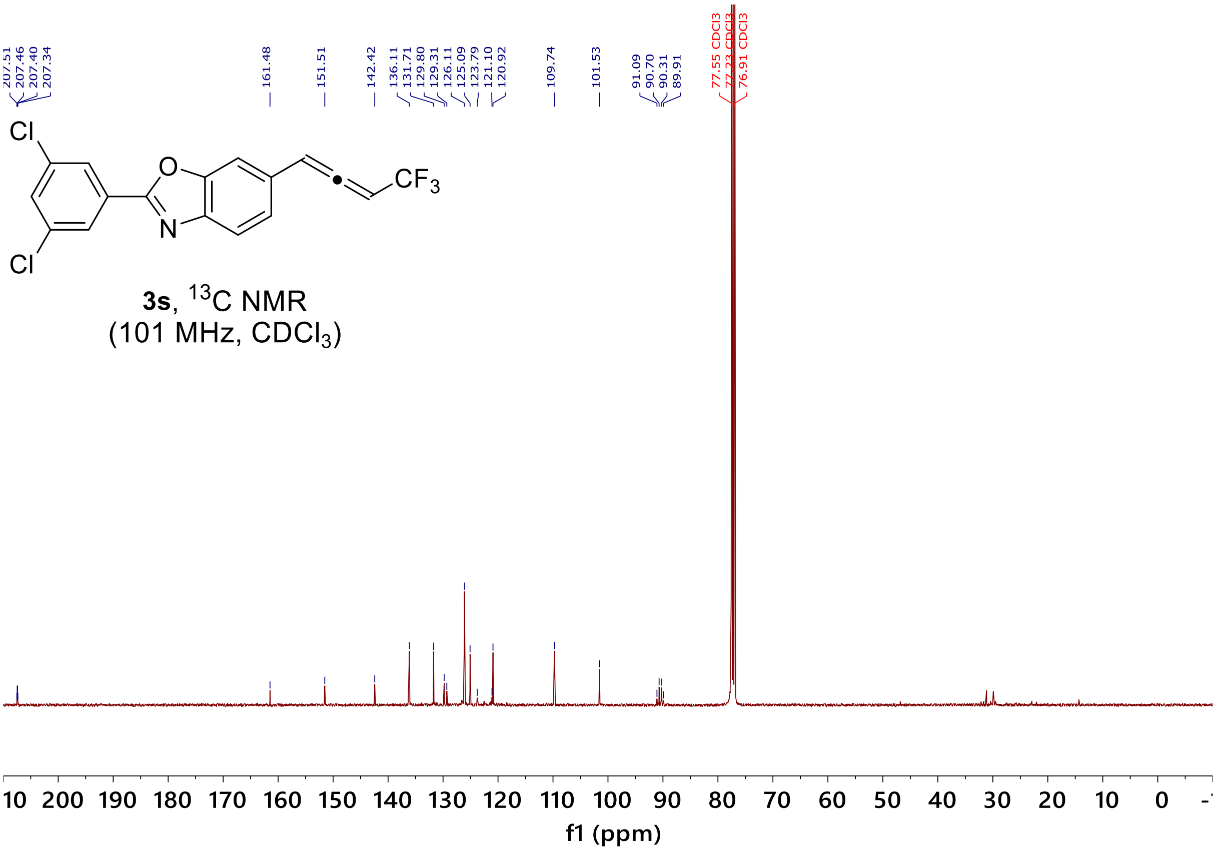

3s, ^{13}C NMR
(101 MHz, $CDCl_3$)

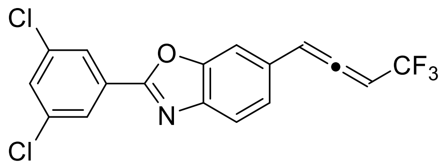

3s, ^{19}F NMR
(376 MHz, $CDCl_3$)

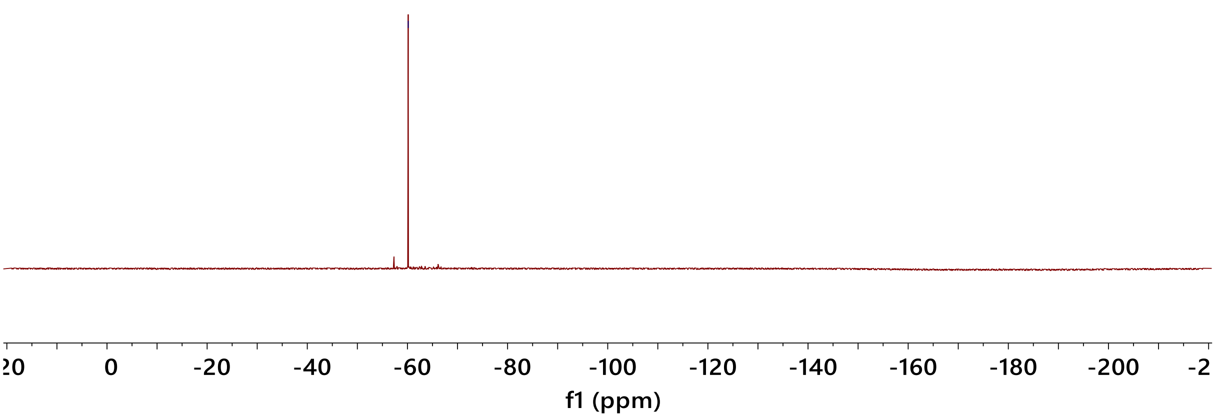

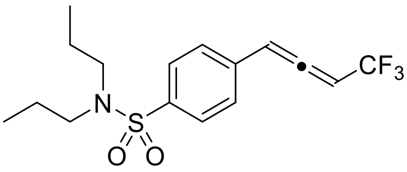

3t, ^{1}H NMR
(400 MHz, $CDCl_3$)

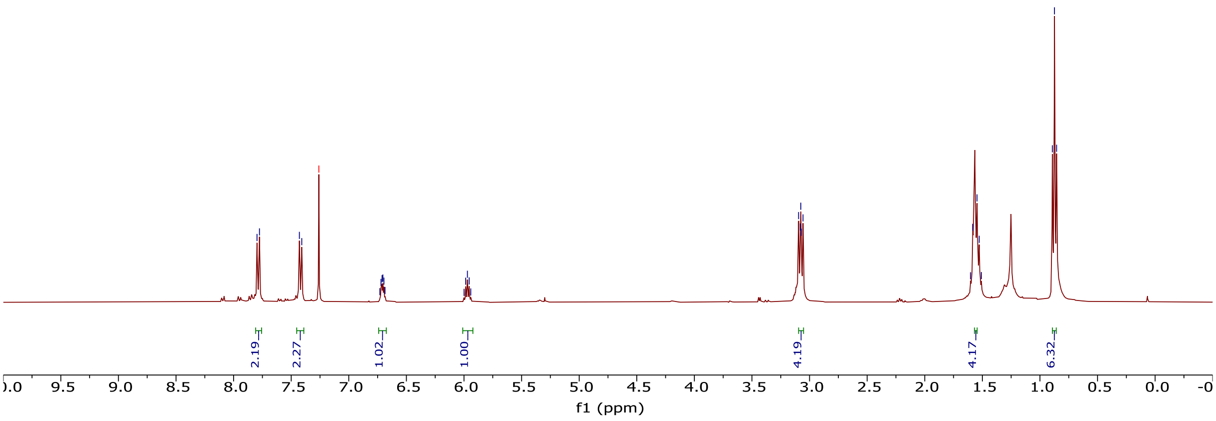

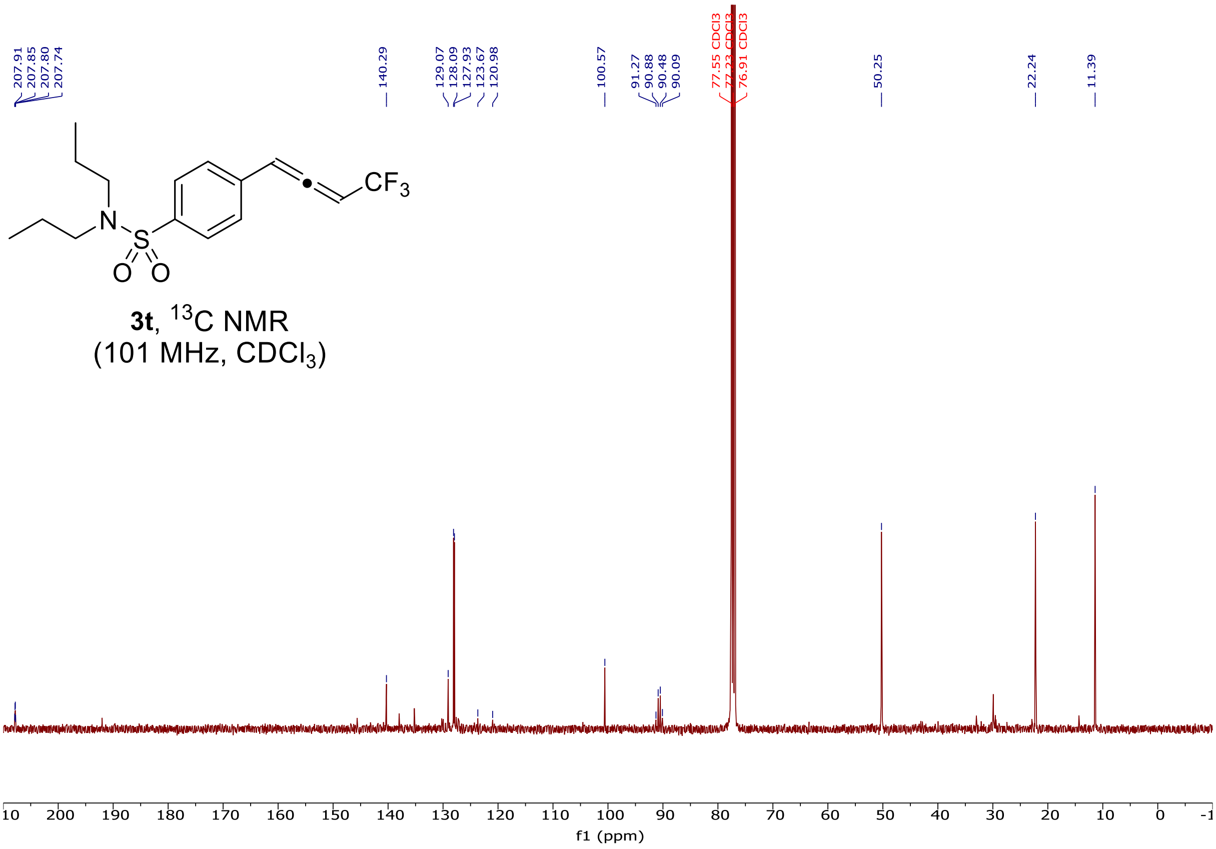

3t, ^{13}C NMR
(101 MHz, $CDCl_3$)

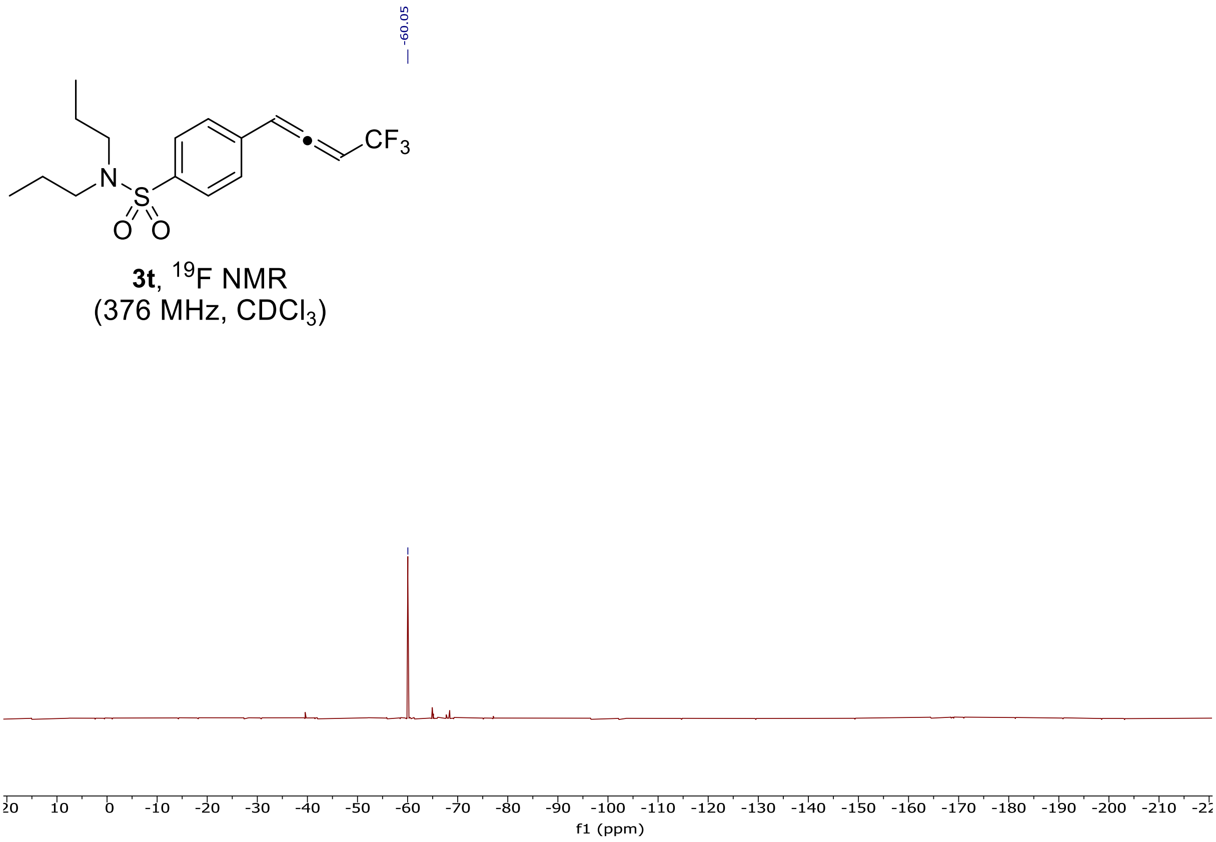

3t, ^{19}F NMR
(376 MHz, $CDCl_3$)

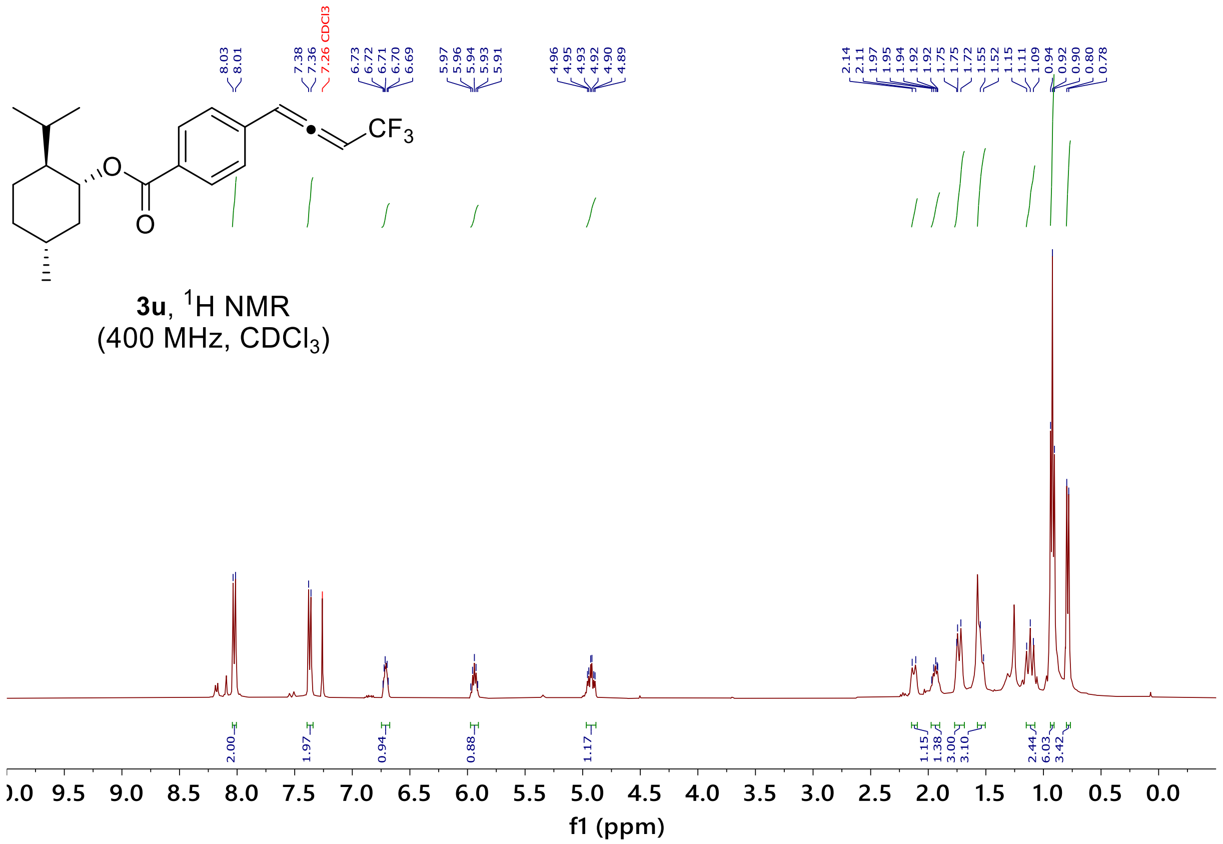
8.03
8.01
7.38
7.36
7.26 CDCl3
6.73
6.72
6.71
6.70
6.69
5.97
5.96
5.94
5.93
5.91
4.96
4.95
4.93
4.92
4.90
4.89
2.14
2.11
1.97
1.95
1.94
1.92
1.92
1.75
1.75
1.72
1.55
1.52
1.15
1.11
1.09
0.94
0.92
0.90
0.80
0.78
CF3
O
O
3u, 1H NMR
(400 MHz, CDCl3)
2.00
1.97
0.94
0.88
1.17
1.15
1.38
3.00
3.10
2.44
6.03
3.42
f1 (ppm)

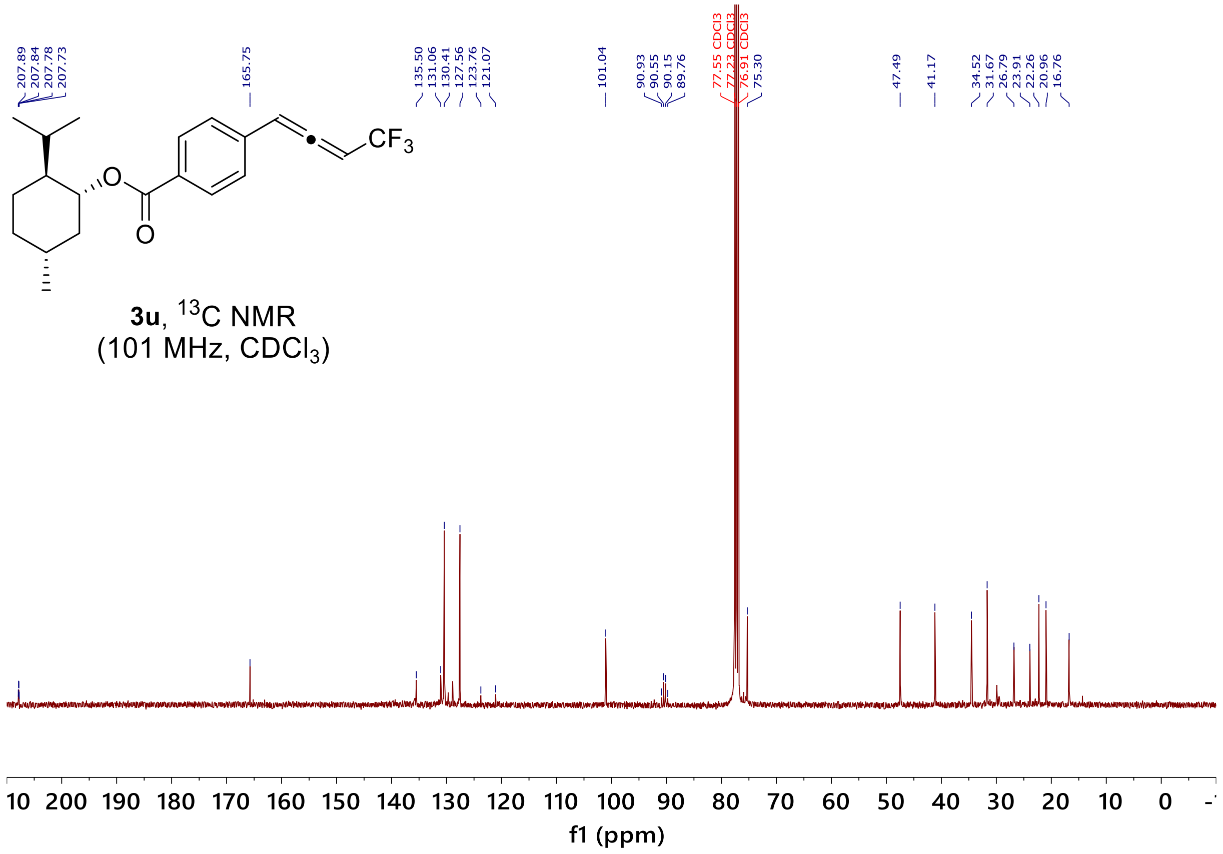
207.89
207.84
207.78
207.73
165.75
135.50
131.06
130.41
127.56
123.76
121.07
101.04
90.93
90.55
90.15
89.76
77.55 CDCl3
77.23 CDCl3
76.91 CDCl3
75.30
47.49
41.17
34.52
31.67
26.79
23.91
22.26
20.96
16.76
CF3
O
O
3u, 13C NMR
(101 MHz, CDCl3)
f1 (ppm)

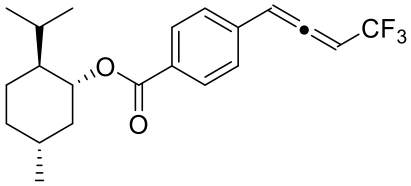

3u, ^{19}F NMR
(376 MHz, $CDCl_3$)

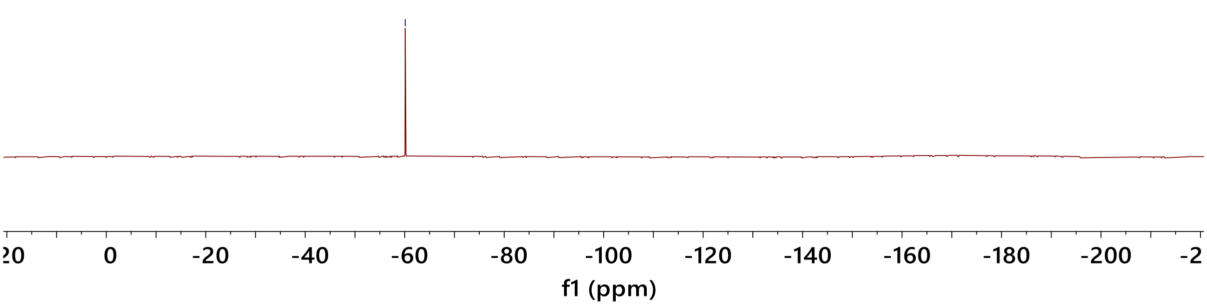

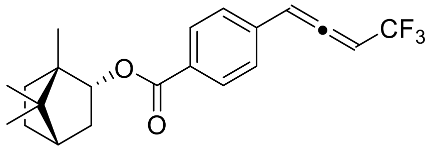

3v, ^{1}H NMR
(400 MHz, $CDCl_3$)

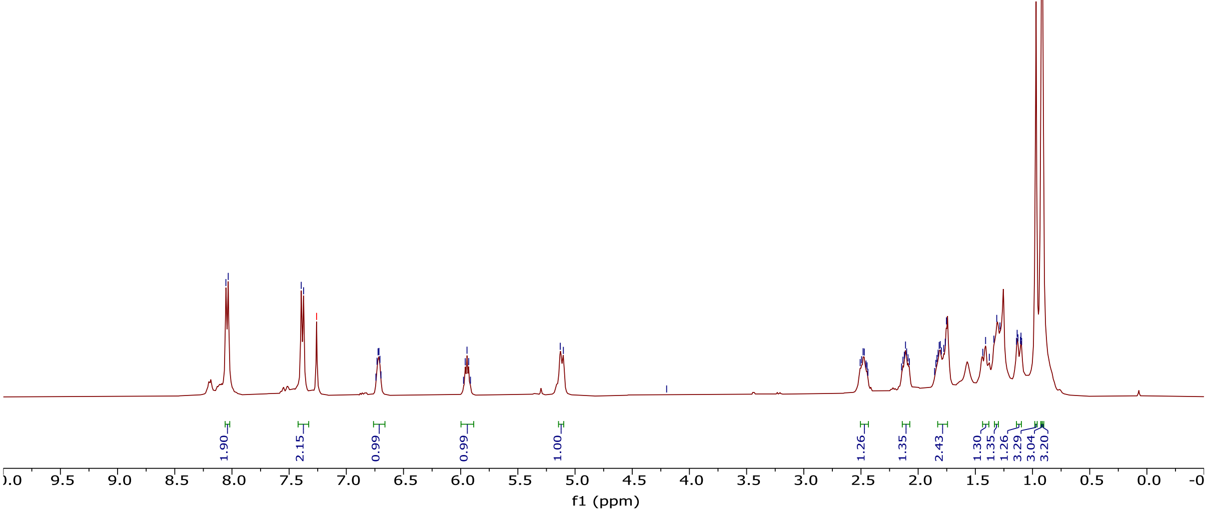

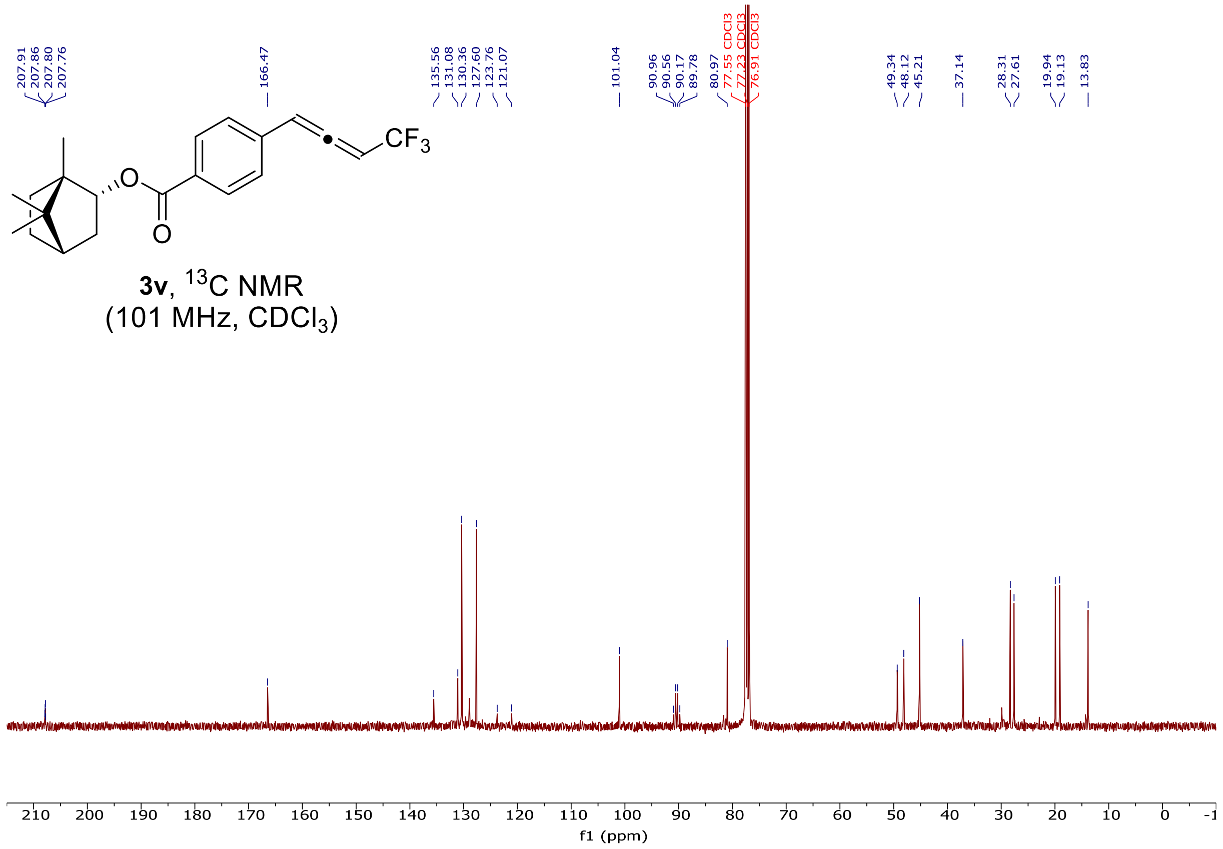

3v, ^{13}C NMR
(101 MHz, $CDCl_3$)

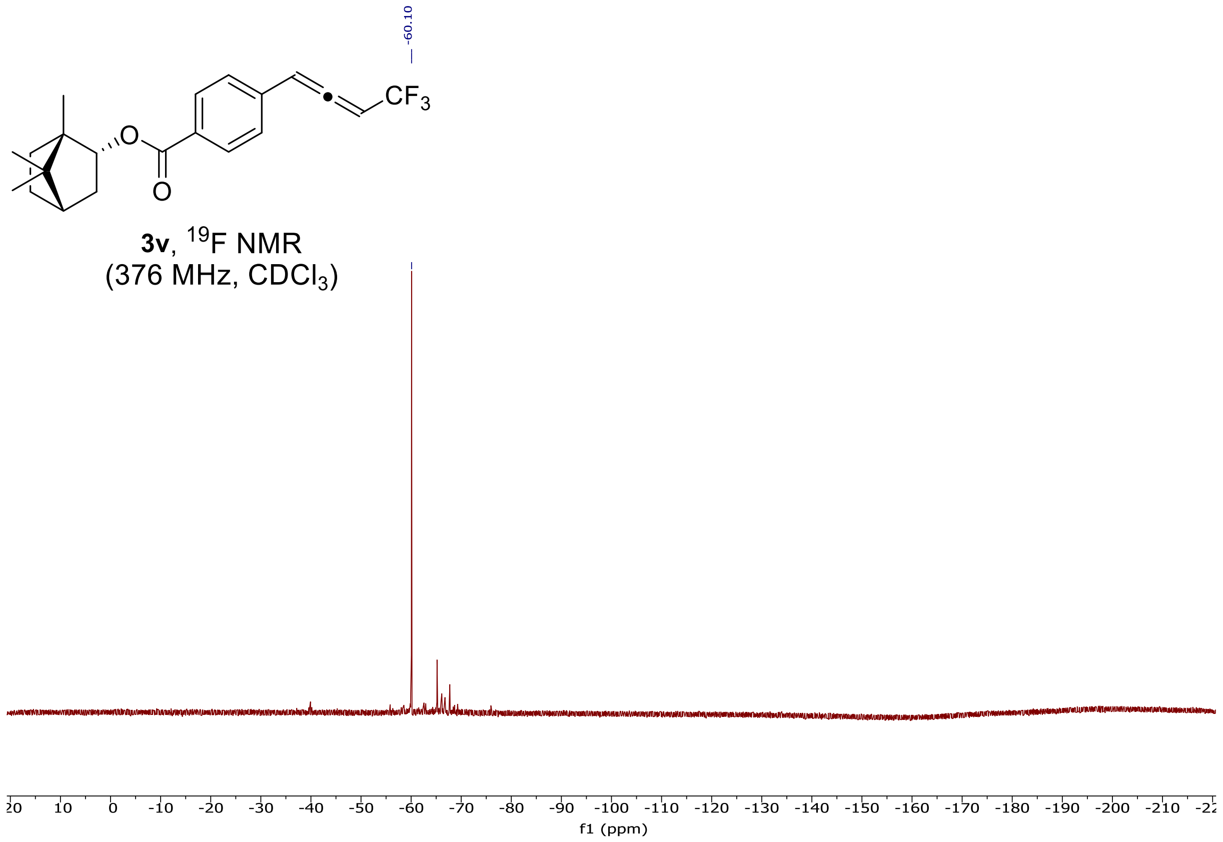

3v, ^{19}F NMR
(376 MHz, $CDCl_3$)

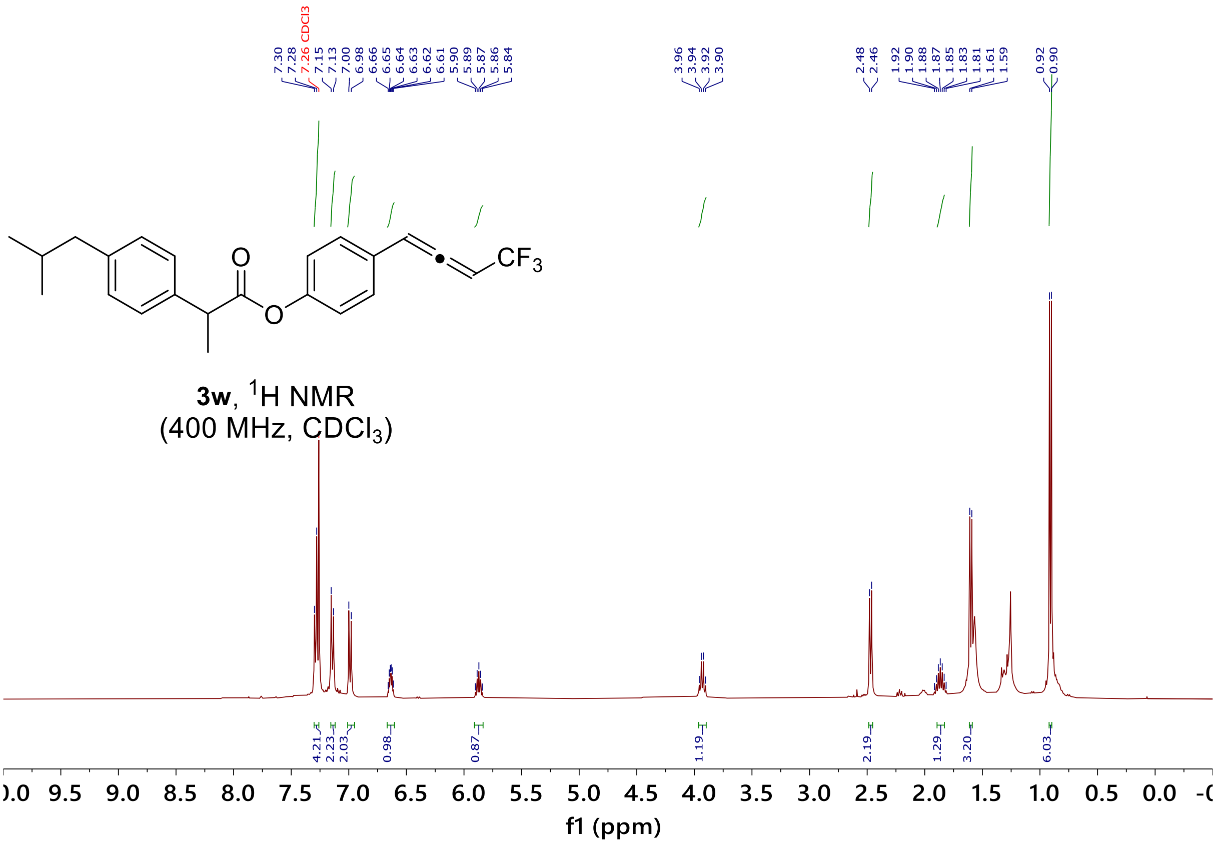
3w, ^{1}H NMR
(400 MHz, $CDCl_3$)
7.30 7.28 7.26 CDCl3 7.15 7.13 7.00 6.98 6.66 6.65 6.64 6.63 6.62 6.61 5.90 5.89 5.87 5.86 5.84
3.96 3.94 3.92 3.90
2.48 2.46 1.92 1.90 1.88 1.87 1.85 1.83 1.81 1.61 1.59
0.92 0.90
4.21 2.23 2.03 0.98 0.87 1.19 2.19 1.29 3.20 6.03
CF3
O
O
f1 (ppm)

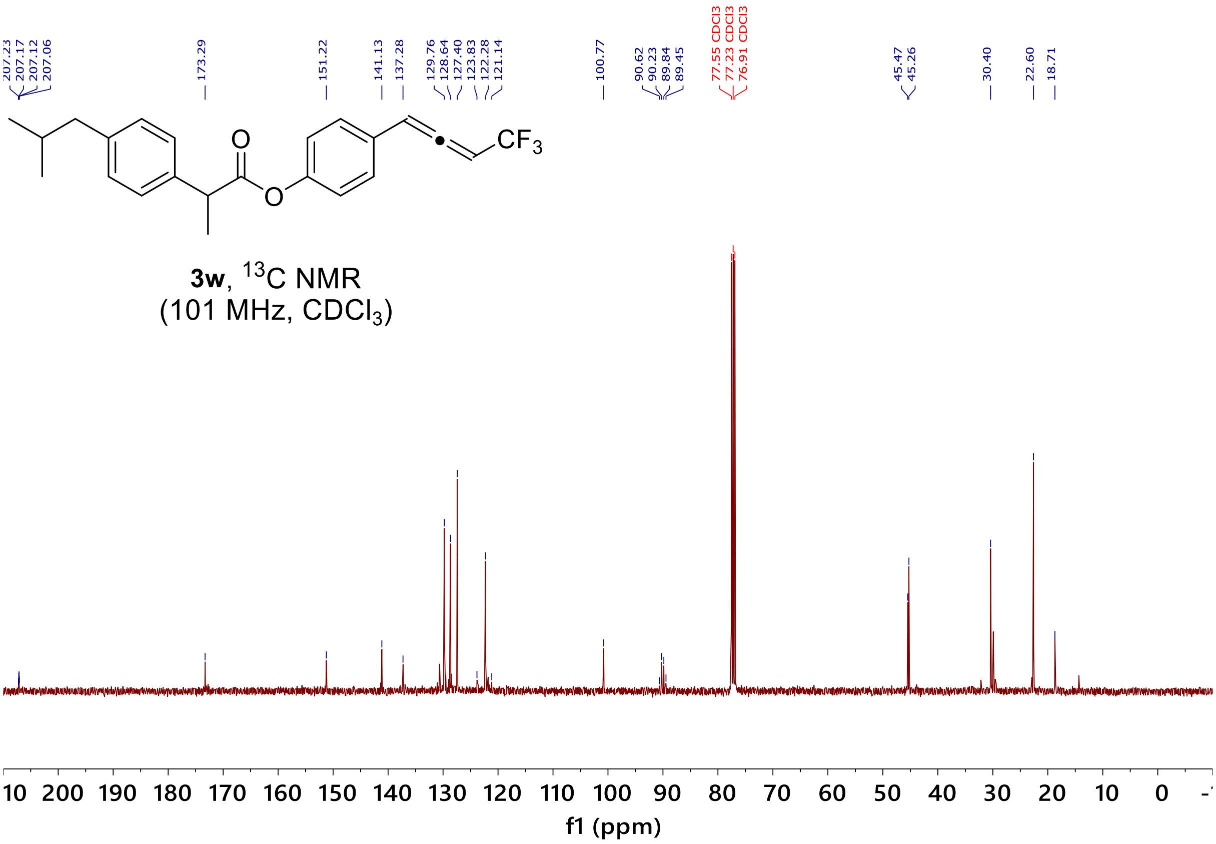
3w, ^{13}C NMR
(101 MHz, $CDCl_3$)
207.23 207.17 207.12 207.06
173.29
151.22
141.13 137.28
129.76 128.64 127.40 123.83 122.28 121.14
100.77
90.62 90.23 89.84 89.45
77.55 CDCl3 77.23 CDCl3 76.91 CDCl3
45.47 45.26
30.40
22.60 18.71
CF3
O
O
f1 (ppm)

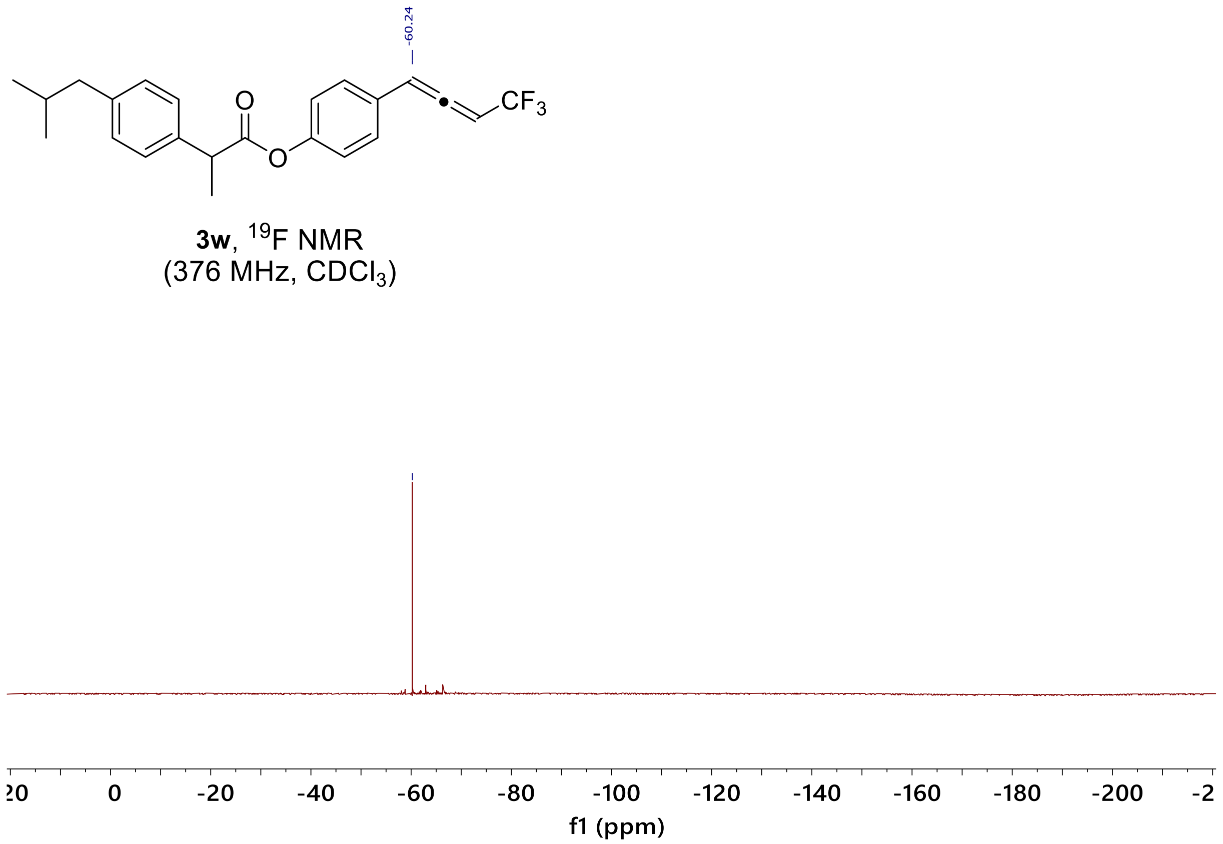

3w, ^{19}F NMR
(376 MHz, $CDCl_3$)

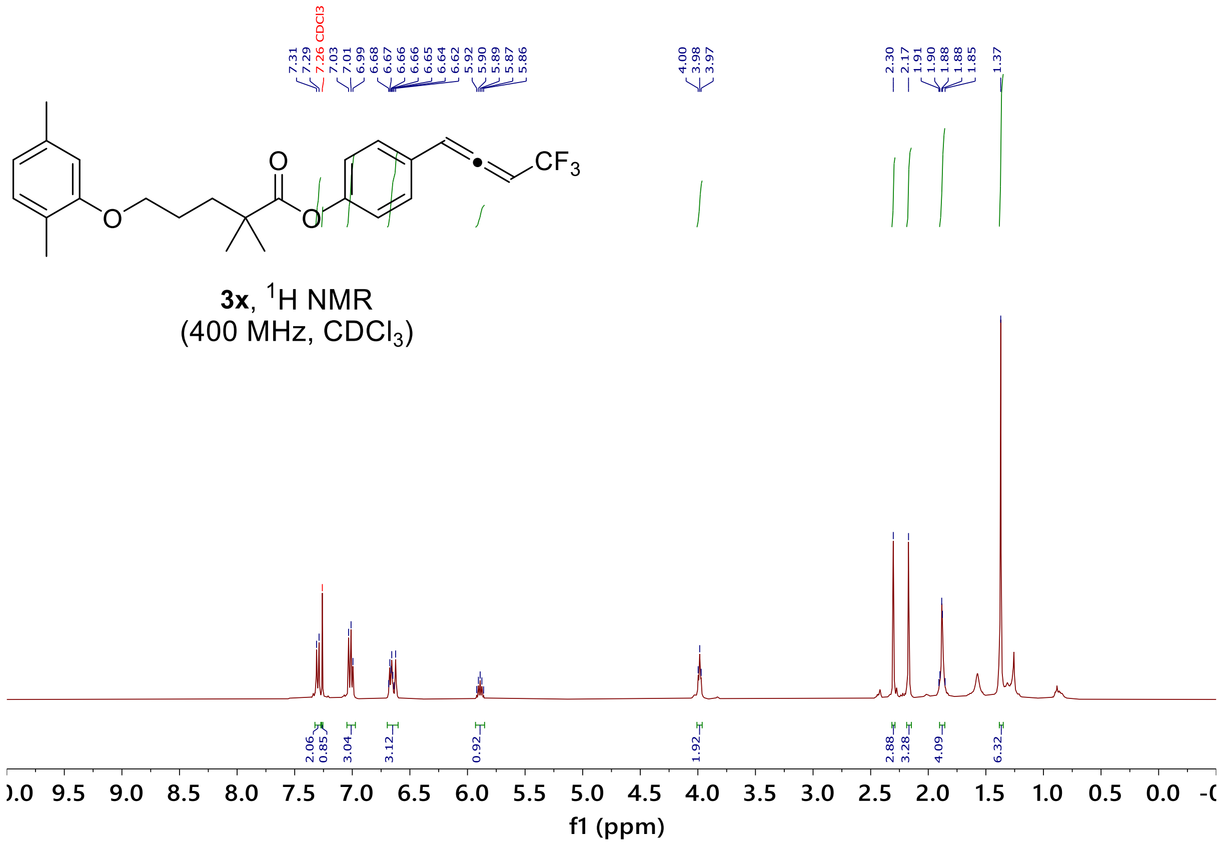

3x, ^{1}H NMR
(400 MHz, $CDCl_3$)

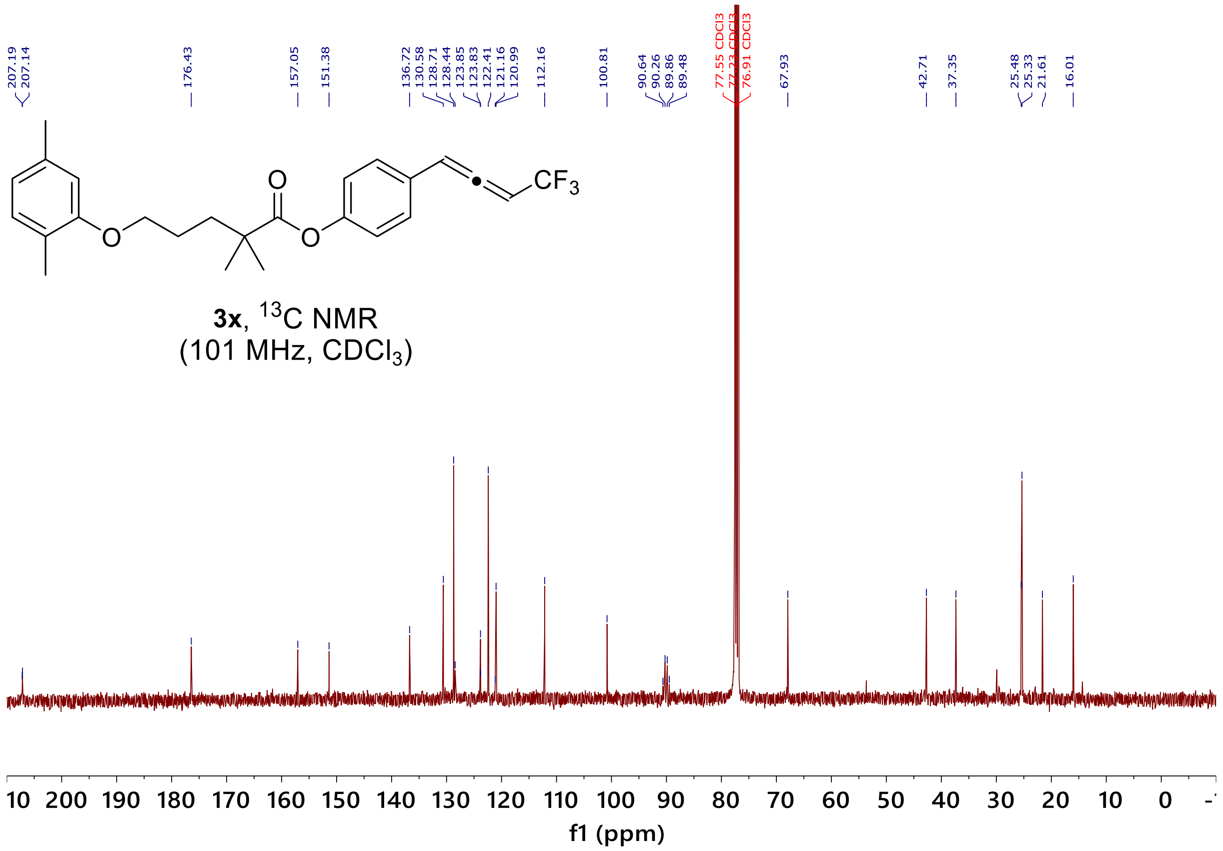

207.19
207.14
176.43
157.05
151.38
136.72
130.58
128.71
128.44
123.85
123.83
122.41
121.16
120.99
112.16
100.81
90.64
90.26
89.86
89.48
77.55 CDCl3
77.23 CDCl3
76.91 CDCl3
67.93
42.71
37.35
25.48
25.33
21.61
16.01
O
O
O
CF3
3x, 13C NMR
(101 MHz, CDCl3)
f1 (ppm)

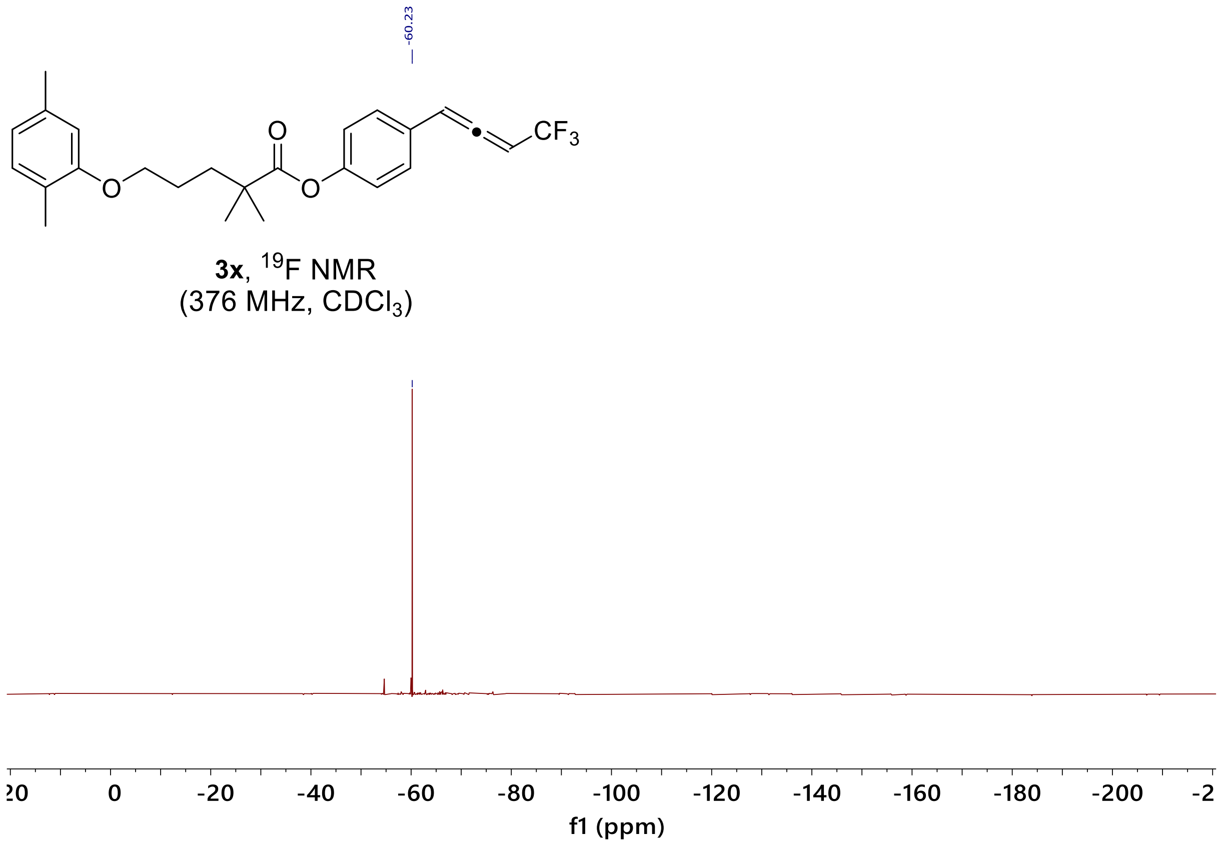

-60.23
O
O
O
CF3
3x, 19F NMR
(376 MHz, CDCl3)
f1 (ppm)

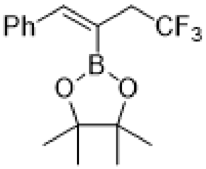

7, ^{1}H NMR
(400 MHz, $CDCl_3$)

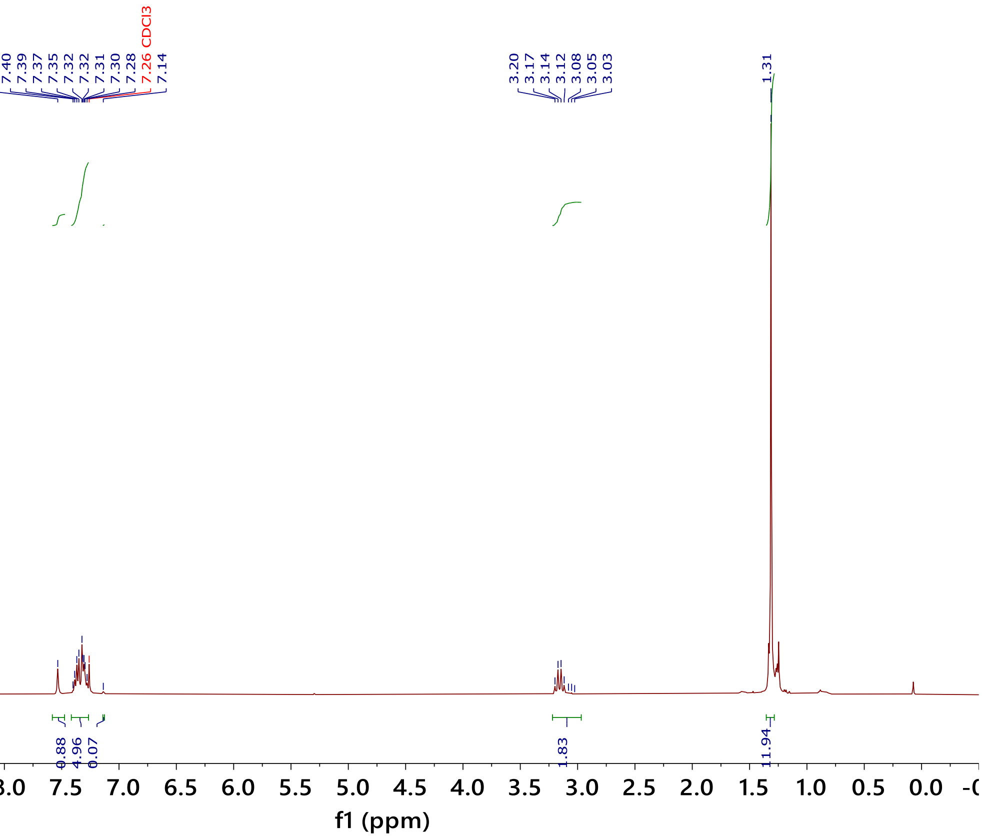

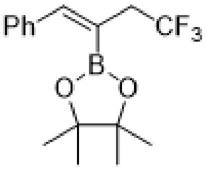

7, ^{13}C NMR
(101 MHz, $CDCl_3$)

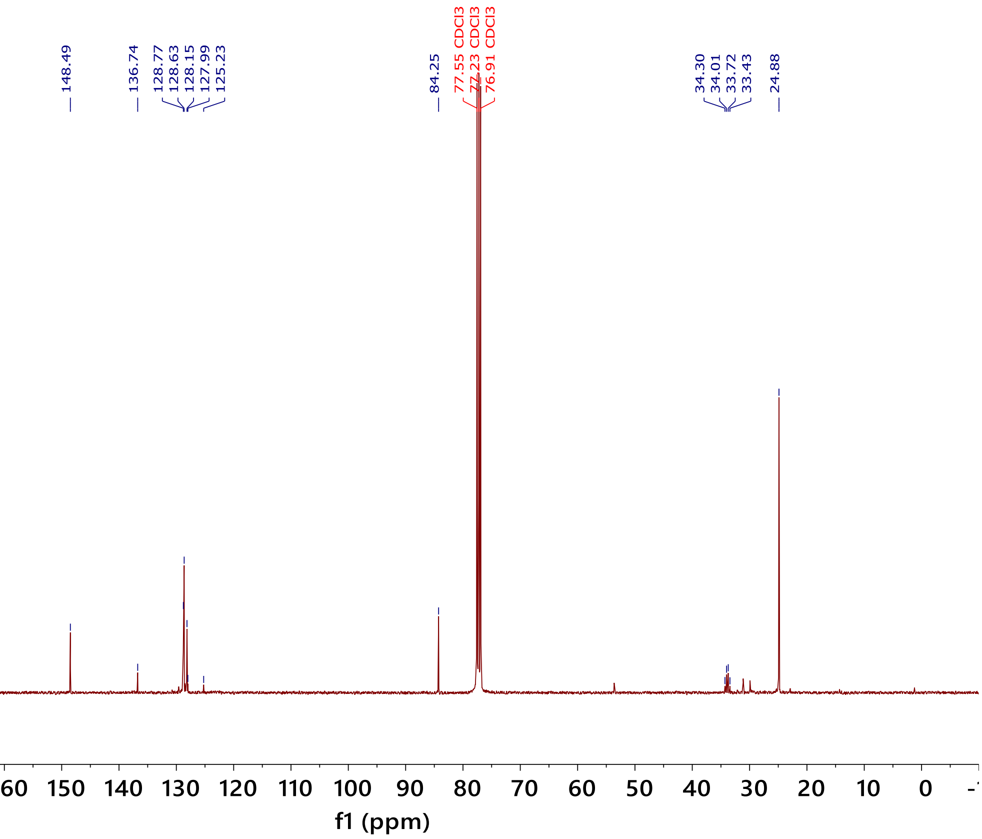

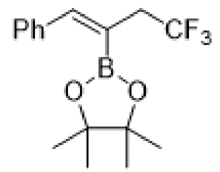

7, ^{19}F NMR
(376 MHz, $CDCl_3$)

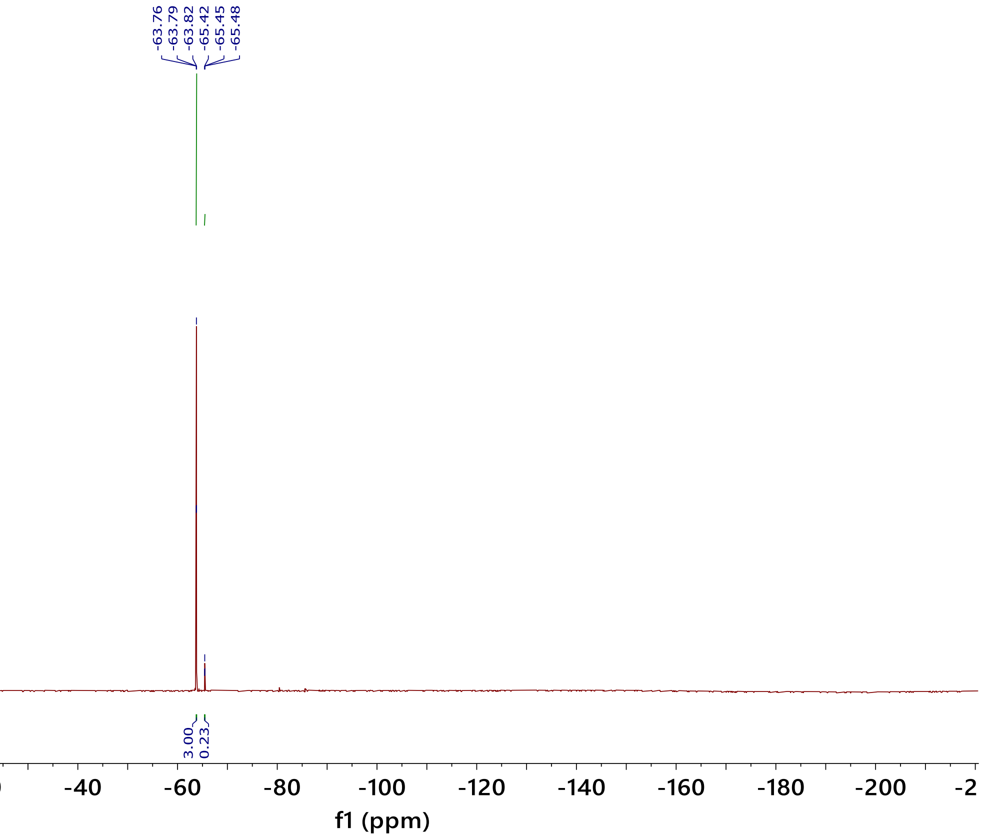

Supplement: SC-017-D6SC02203K-s001 [file SC-017-D6SC02203K-s001.pdf]
